# Electronic effects on the stability of heteroleptic nickel(II) complexes with aromatic and aliphatic ligands

**DOI:** 10.1007/s00894-026-06833-1

**Published:** 2026-07-08

**Authors:** Daniella B. de Miranda, Thalita R. André, Glaucio B. Ferreira

**Affiliations:** Instituto de Química, Universidade Federal Fluminense, Outeiro de S. João Batista S/N, Centro, Niterói, RJ 24210-130 Brazil

**Keywords:** Xanthate, Nickel, Heteroleptic, M06L, NBO, DFT

## Abstract

**Context:**

Heteroleptic metal complexes are attractive systems because their properties can be tuned by combining ligands with distinct donor atoms and electronic characteristics. Xanthates are versatile sulfur-donor ligands that influence the stability and electronic structure of metal complexes. Nickel(II) complexes containing xanthate and nitrogen-donor ligands are therefore suitable models for investigating how sulfur- and nitrogen-based coordination environments affect thermochemical stability and excited-state properties. Here, six Ni(II) complexes containing *n*-butyl xanthate (**L1**) or 2-methoxyethyl xanthate (**L2**), combined with ethylenediamine (en), 2,2′-bipyridine (bpy), or 1,10-phenanthroline (phen), were investigated. The results indicate that the formation of these heteroleptic complexes is thermodynamically favorable and governed by steric and electronic ligand effects.

**Methods:**

DFT calculations were performed using the M06L/def2-TZVP level of theory, chosen for its reliable description of transition metal systems, noncovalent interactions, and thermochemical properties at moderate computational cost. Geometry optimizations were carried out for six heteroleptic Ni(II) complexes ([Ni(en)(L1)_2_], [Ni(en)(L2)_2_], [Ni(bpy)(L1)_2_], [Ni(bpy)(L2)_2_], [Ni(phen)(L1)_2_], and [Ni(phen)(L2)_2_]). Vibrational frequency and thermochemical analyses, including BSSE corrections, were also performed. TD-DFT calculations were performed to simulate the UV–Vis spectra, considering up to 300 vertical excitations to cover the 200–800 nm spectral window. Solvent effects were considered using CPCM, with ethanol for thermochemistry/geometry and acetonitrile for TD-DFT UV–Vis simulations. Electronic transitions were analyzed using NBO/CMO analysis with Gaussian 09 and GaussSum.

**Graphical Abstract:**

**Figure:**
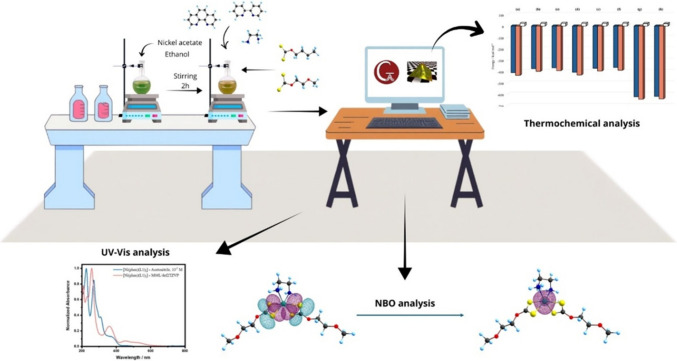

**Supplementary Information:**

The online version contains supplementary material available at 10.1007/s00894-026-06833-1.

## Introduction

Xanthates (ROCS_2_^−^) are dithiocarbonate ligands widely used in coordination chemistry. They are well known for their role as collecting agents in the ore flotation process and in biological applications, such as antifungal, antimicrobial, antioxidant, and antitumor [1–18]. Due to the presence of electron-donating atoms in their structure, xanthates can coordinate with different metal centers, exhibiting a high chelating capacity. This has led to the synthesis of numerous xanthate complexes and the study of their properties. Among these, heteroleptic complexes have been extensively investigated due to their applications in different areas [19–30]. Although these systems are promising, there are still few studies involving heteroleptic xanthate complexes with nitrogen-donor ligands. In addition, for new applications and biological activities to be explored, it is essential to understand the chemical nature of these compounds and to establish correlations between structure and the properties relevant to their development. Spectroscopic techniques are essential for structural characterization and for understanding the vibrational and electronic properties of these systems. In addition to experimental spectroscopic techniques, theoretical methods based on Density Functional Theory (DFT) have been widely employed for the calculation and interpretation of spectroscopic data, supporting structural assignment. Although still relatively scarce, studies in the literature demonstrate that xanthates have been investigated through spectroscopic approaches combining experimental data and DFT calculations [15, 31, 32]. Despite these advances, a detailed understanding of the structural, vibrational, and electronic properties of heteroleptic nickel(II) xanthate complexes remains limited. Therefore, this work presents a computational study supported by experimental spectroscopic data for six heteroleptic Ni(II) complexes containing xanthate and nitrogen-donor ligands. This study aims to elucidate how the electronic nature of sulfur and nitrogen-donor ligands governs metal–ligand interactions, thermodynamic stability, and excited-state properties. The complexes were synthesized and characterized by UV–Vis spectroscopy in solution. The electronic transitions were assigned using TD-DFT calculations, while NBO analysis was employed to visualize the orbitals involved. In addition, second-order perturbation theory (E^2^) analysis was used to evaluate donor–acceptor interactions and to assess the covalent character of the Ni–S and Ni–N bonds, using homoleptic xanthate complexes as reference systems within the NBO E^2^ analysis.

## Methods

### Synthesis

Initially, the n-butyl xanthate (**L1**) and 2-methoxyethyl xanthate (**L2**) salts were synthesized according to procedures described in the literature [31]. In both syntheses, commercially available KOH (1.68 g, 30 mmol) and carbon disulfide (CS_2_, 1.8 mL, 45 mmol) were used. *n*-Butanol and 2-methoxyethanol (20 mL) were used both as solvents and reagents and were converted into the corresponding alkoxides. Subsequent reaction with CS_2_ led to the formation of *n*-butyl xanthate and 2-methoxyethyl xanthate, respectively.

Using the xanthates obtained in the previous step, six heteroleptic nickel(II) complexes were synthesized with the ligands ethylenediamine (en), 2,2′-bipyridine (bpy), and 1,10-phenanthroline (phen): [Ni(en)(L1)_2_], [Ni(en)(L2)_2_], [Ni(bpy)(L1)_2_], [Ni(bpy)(L2)_2_], [Ni(phen)(L1)_2_], and [Ni(phen)(L2)_2_]. The complexes were synthesized according to procedures described in the literature [30]. An ethanolic solution of nickel(II) acetate was prepared, followed by ligand addition using a 1:1:2 molar ratio of Ni(II) precursor, N-donor ligand, and xanthate.

#### [Ni(en)(L1)_2_]

IR (ATR) $\widetilde{\nu }$/cm^−1^ 3274, 3163, 2952, 2917, 2872, 1581, 1452, 1404, 1390, 1337, 1330, 1272, 1108, 1021, 993, 868, 684, 651, 615; Raman $\widetilde{\nu }$/cm^−1^ 2944, 2879, 1460, 1390, 1332, 1276, 1110, 996, 983, 920, 880, 656, 447; UV–Vis λ/nm (acetonitrile–10^–5^ M): 310, 274, 244, 228, 215, 180; m.p. = 173–176 °C; Yield = 70%.

#### [Ni(en)(L2)_2_]

IR (ATR) $\widetilde{\nu }$/cm^−1^ 3274, 3232, 3161, 3111, 2924, 2876, 1581, 1451, 1395, 1367, 1329, 1271, 1235, 1148, 1111, 1020, 993, 867, 829, 687, 649; Raman $\widetilde{\nu }$/cm^−1^ 2936, 2884, 1582, 1470, 1275, 1105, 1028, 881, 843, 671, 533, 476, 441, 371, 306, 214; UV–Vis λ/nm (acetonitrile–10^–5^ M): 364, 310, 282, 246, 229, 189; m.p. = 184–188 °C; Yield = 85%.

#### [Ni(bpy)(L1)_2_]

IR (ATR) $\widetilde{\nu }$/cm^−1^ 2957, 2931, 2868, 1599, 1571, 1563, 1489, 1470, 1441, 1381, 1308, 1254, 1228, 1184, 1133, 1055, 1042, 1018, 990, 960, 928, 902, 841, 812, 768, 733, 651, 632; Raman $\widetilde{\nu }$/cm^−1^ 1653, 1598, 1556, 1527, 1483, 1318, 1272, 1197, 1166, 1024; UV–Vis λ/nm (acetonitrile–10^–5^ M): 343, 307, 290, 273, 258, 242, 224, 185; m.p. = 121–123 °C; Yield = 58%.

#### [Ni(bpy)(L2)_2_]

IR (ATR) $\widetilde{\nu }$/cm^−1^ 2983, 2942, 2928, 2888, 2856, 2835, 2821, 1596, 1571, 1560, 1488, 1467, 1432, 1399, 1368, 1291, 1242, 1194, 1155, 1120, 1097, 1053, 1040, 1007, 898, 867, 837, 767, 732, 672, 651, 630, 568; Raman $\widetilde{\nu }$/cm^−1^ 1598, 1556, 1485, 1317, 1271, 1165, 1023, 765; UV–Vis λ/nm (acetonitrile–10^–5^ M): 307, 305, 292, 275, 266, 251, 238, 219, 197; m.p. = 135–138 °C; Yield = 79%.

#### [Ni(phen)(L1)_2_]

IR (ATR) $\widetilde{\nu }$/cm^−1^ 2952, 2931, 2868, 1622, 1602, 1580, 1510, 1495, 1461, 1421, 1382, 1341, 1315, 1302, 1237, 1217, 1190, 1173, 1142, 1125, 1105, 1043, 993, 847, 842, 810, 781, 768, 746, 724, 661, 641; Raman $\widetilde{\nu }$/cm^−1^ 1575, 1504, 1447, 1295, 1205, 735; UV–Vis λ/nm (acetonitrile–10^–5^ M): 463, 366, 303, 248, 268, 225, 196; m.p. = 130–132 °C; Yield = 26%.

#### [Ni(phen)(L2)_2_]

IR (ATR) $\widetilde{\nu }$/cm^−1^ 3055, 2935, 2890, 2828, 1624, 1582, 1514, 1494, 1457, 1433, 1421, 1369, 1339, 1292, 1246, 1156, 1119, 1099, 1051, 1015, 903, 849, 778, 724, 669, 642, 616, 559; Raman $\widetilde{\nu }$/cm^−1^ 1511, 1422, 1338, 1300, 1277, 1501, 1056; UV–Vis λ/nm (acetonitrile–10^–5^ M): 333, 309, 291, 267, 225, 195; m.p. = 167–169 °C; Yield = 73%.

### Computational details

In this work, density functional theory (DFT) was used, with the M06L functional and def2-TZVP basis set [33, 34]. DFT is a well-established and widely applied approach for the investigation of complex molecular systems, particularly in spectroscopic studies [35]. The M06L functional was selected based on its documented performance for transition-metal systems and noncovalent interactions [33]. Furthermore, it offers moderate computational costs, making it suitable for calculations involving complexes and large molecular systems. This functional also provides good accuracy for describing thermochemical trends, as well as geometric and vibrational frequency parameters [33]. However, although M06L is an appropriate choice for calculations involving transition metal systems, certain limitations may affect the electronic analysis. As a local meta-GGA functional that lacks Hartree–Fock exchange, M06L can be less reliable for systems exhibiting strong multireference character. Furthermore, it may not accurately describe long-range charge-transfer states, as the correct behavior of the exchange–correlation potential is not fully reproduced. Therefore, the excitation energies obtained from TD-DFT using M06L are interpreted with the expected uncertainties in mind, depending on the nature of the excited states [36]. The def2-TZVP basis set was used due to the balanced description it provides for various elements of the periodic table. In addition, the triple-zeta set was chosen because it exhibits lower errors in atomization energies per atom at the DFT level compared to other smaller basis sets from the same family [34]. Once the level of theory was chosen, geometry optimization and vibrational frequency calculations were performed in ethanol (CPCM) for both the heteroleptic complexes and the reagents [37]. Based on the energies obtained, a thermochemical analysis of the reactions was performed to investigate the formation trends of the systems. For this purpose, corrections for basis set superposition errors (BSSE) were applied using the counterpoise procedure; and the BSSE-corrected thermochemical values are provided in the Supplementary Information. Using the optimized structures, TD-DFT calculations were performed to compare the calculated UV–Vis spectra with the experimental data. Triplet states were considered for heteroleptic complexes and singlet states for homoleptic references. TD-DFT calculations included up to 300 vertical excitations to ensure full coverage of the 200–800 nm spectral window. The inclusion of many excited states was necessary to provide a better description of the absorption profile in the ultraviolet and visible regions. This high number of states is essential for the description of transition metals due to the high density of states (DOS) derived from the metal d-orbitals. Increasing the configuration interaction space in the linear response reduces truncation errors, improving the accuracy of electronic transitions [38]. Band assignments were based on transitions within this range with non-negligible oscillator strengths and dominant orbital contributions. For this purpose, the structures were also optimized using the conductor-like polarizable continuum model (CPCM), with acetonitrile as the solvent [39]. The assignment of the orbitals involved in the main electronic transitions observed was carried out using natural bond orbitals (NBO) through canonical molecular orbitals (CMO). All these calculations were performed using the Gaussian 09 and GaussSum software packages [39, 40].

## Results

This section presents the main results, organized into synthesis and spectroscopic characterization, thermochemical analysis, UV–Vis electronic analysis, NBO analysis, and acid–base hardness/softness descriptors. In addition, to verify the formation of the synthesized compounds, characterizations were performed using infrared absorption vibrational spectroscopy and Raman scattering techniques. The melting points of the compounds are provided in the Methods section and are consistent with good sample quality, with melting ranges varying between 2 and 3 °C. The experimental spectra of the xanthates, as well as those of the complexes, are provided in the synthesis section of the supplementary material (Figures S1–S5 and Table S1). In addition, all compounds were previously optimized before the computational analyses performed in this work. Figures S6 and S7, as well as Tables S2 and S3, present data obtained from the optimized structures, in ethanol (CPCM).

The Ni–N and Ni–S bond lengths observed in the optimized structures were compared with experimental data reported in the literature, as well as the S–Ni–S, N–Ni–N, and N–Ni–S bond angles [30]. Thus, the experimental Ni–N bond lengths range from 2.164 to 2.177 Å for a heteroleptic xanthate complex with the en ligand. In the calculated structures, values ranging from 2.140 to 2.147 Å were observed for the analogous complexes. The Ni–S bonds also show good agreement, exhibiting experimental values of 2.449–2.467 Å, while the optimized structures present a range of 2.459–2.485 Å, for all the heteroleptic complexes investigated (Table S2). For the complexes with aromatic ligands, no crystallographic data were found in the literature in which both a nitrogen-containing ligand and a xanthate are coordinated to the Ni(II) center, as observed for en. However, other heteroleptic complexes in the literature exhibit Ni–N bond lengths of 2.09–2.10 Å and 1.96–2.27 Å for complexes with bpy and phen, respectively [41, 42]. For the aromatic complexes, the optimized Ni–N bond lengths are 2.097–2.100 Å (bpy) and 2.109–2.121 Å (phen), while the Ni–S distances fall in the 2.436–2.476 Å range (Table S2). In the case of the homoleptic complexes, good theoretical–experimental correlations were also observed. Literature data report Ni–S bond lengths of 2.23–2.24 Å [43], which is also the range observed for the optimized structures (Table S2). In addition to the main bond lengths of the Ni(II) complexes, the angles formed between nickel, nitrogen, and sulfur atoms also showed good theoretical–experimental agreement. According to experimental data for heteroleptic complexes, the S–Ni–S, N–Ni–N, and N–Ni–S angles are in the ranges of 72.48–97.80°, 83.60–83.78°, and 92.80–97.80°, respectively. Table S3 shows that, for the optimized structures, these angles present ranges of 72.89–99.55°, 77.65–81.45°, and 90.97–96.93°, respectively. For the homoleptic complexes, the S–Ni–S angles have a value of 79.50° in the literature [43], while the calculated structures exhibit a range of 78.74–101.37°. These results indicate that the level of theory used for the optimization calculations is appropriate for describing both homoleptic and heteroleptic xanthate complexes.

### Syntheses and spectroscopic characterization

**Fig. 1:**
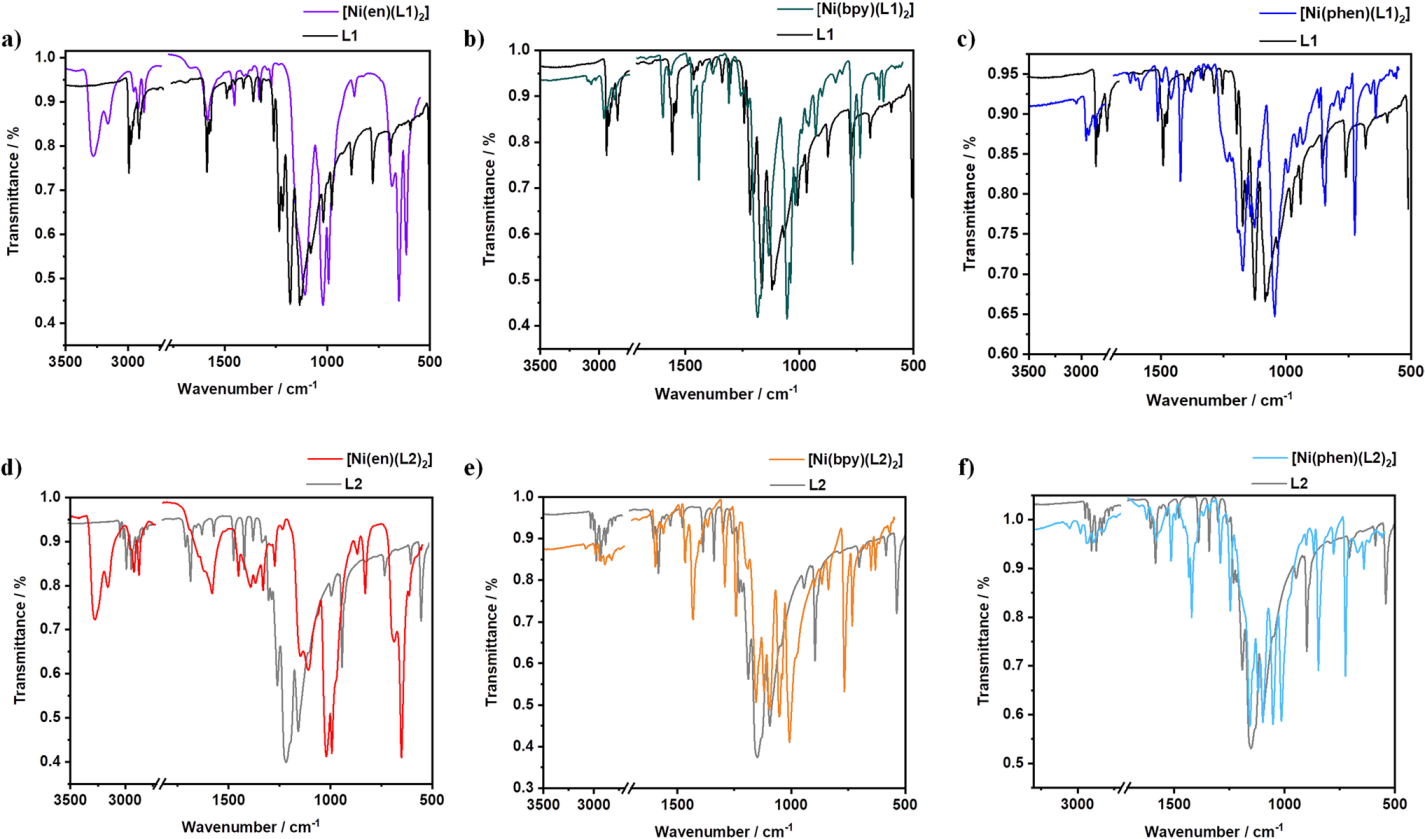
Mid-IR spectra of the following: [Ni(en)(L1)_2_] (**a**); [Ni(bpy)(L1)_2_] (**b**); [Ni(phen)(L1)_2_] (**c**); [Ni(en)(L2)_2_] (**d**); [Ni(bpy)(L2)_2_] (**e**); [Ni(phen)(L2)_2_] (**f**), compared with the corresponding xanthate salts (L1 and L2)

In this section, the experimental data and observations related to the synthesis of the heteroleptic complexes are presented and discussed. The formation of the complexes and the coordination of the ligands were evaluated through comparison of the IR, Raman, and UV–Vis spectra with those of the corresponding xanthate salts. Figure 1 presents the IR spectra of the complexes in comparison with *n*-butyl xanthate (L1) and 2-methoxyethyl xanthate (L2).

For the mid-infrared spectra of the xanthate salts (Figure S1), the characteristic bands of the xanthate group were observed at approximately 1062, 1099, 903, and 672 cm^−1^, assigned to the coupled stretching υ_coupled_C–O, antisymmetric stretching υ_ass_C–S, υC–O stretching, and symmetric stretching υ_s_C–S vibrational modes, respectively [44]. The experimental infrared absorption spectra of the heteroleptic complexes (Figure S2) revealed the presence of these main vibrational modes in the mid-IR region, supporting the presence of xanthate ligands in the complexes. The assignment of these bands was confirmed through comparison with the spectra of the isolated ligands (Fig. 1), while the values associated with each vibrational mode are summarized in Table 1. Compared with the precursor ligands, a slight shift of the bands toward lower wavenumbers was observed, which can be assigned to the increase in reduced mass resulting from complexation. Thus, the υ_coupled_C–O, υ_ass_C–S, υC–O, and υ_s_C–S vibrational modes of the heteroleptic complexes were observed at approximately 1180, 1037, 979, and 651 cm^−1^, respectively. In addition to the bands assigned to xanthates, the spectra of the complexes exhibited bands associated with vibrational modes involving nitrogen atoms, indicating the presence of the nitrogen-containing ligand in the complex structure. For the [Ni(en)(L1)_2_] and [Ni(en)(L2)_2_] complexes, bands were observed at 3274 and 3163 cm^−1^ and at 3232 and 3111 cm^−1^, respectively, assigned to υN–H stretching vibrations [45]. For complexes containing aromatic nitrogen ligands (bpy and phen), the main bands associated with the aromatic rings arise from C = C and C = N stretching vibrations as well as in-plane and out-of-plane ring deformation modes. According to the literature, bpy exhibits characteristic bands around 1600 cm^−1^ (C = C stretching), 1440 cm^−1^ (coupled C = C and C = N stretching), and 1020 cm^−1^ (in-plane ring deformation). For phen, characteristic bands are observed at approximately 1500 cm^−1^ (coupled C = C and C = N stretching), 1100 cm^−1^ (in-plane ring deformation), and 730 cm^−1^ (out-of-plane ring deformation) [46]. Table 1 presents the experimental data of the investigated complexes related to these vibrational modes, whose values are consistent with the ranges reported in the literature.

**Table 1:** Main IR vibrational modes of the investigated complexes (cm^−1^)

	**Vibrational modes of xanthates ligands (cm**^**−1**^**)**						
	**υC**–**O**_**(coupled)**_	**υ**_**ass**_**C**–**S**	**υC**–**O**	**υ**_**s**_**C**–**S**	**δC**–**O**–**C**	**δS**–**C**–**S**	**υNi**–**S**
**[Ni(en)(L1)**_**2**_**]**	1273	1020	992	651	497	402	278
**[Ni(en)(L2)**_**2**_**]**	1145	1019	990	651	481	408	280
**[Ni(bpy)(L1)**_**2**_**]**	1184	1040	990	672	501	382	277
**[Ni(bpy)(L2)**_**2**_**]**	1155	1053	1005	649	501	376	288
**[Ni(phen)(L1)**_**2**_**]**	1173	1043	993	663	499	384	294
**[Ni(phen)(L2)**_**2**_**]**	1157	1051	902	640	508	385	293
**L1**	1060	1101	923	669	537	280	-
**L2**	1064	1098	883	675	532	279	-
	**Vibrational modes of nitrogen ligands (cm**^**−1**^**)**						
	**υNi**–**N**			**υ (N–H) (cm**^**−1**^**)**			
**[Ni(en)(L1)**_**2**_**]**	326			3274, 3163			
**[Ni(en)(L2)**_**2**_**]**	326			3232, 3111			
	**υNi**–**N**	**υC = C**_**(arom. ring)**_		**υ**_**coupled**_**C = C/C = N**		**Ring deformation (in-plane)**	
**[Ni(bpy)(L1)**_**2**_**]**	335	1491		1598, 1469		1017	
**[Ni(bpy)(L2)**_**2**_**]**	336	1488		1596, 1468		1039	
	**υNi**–**N**	**υ**_**coupled**_**C = C/C = N**		**Ring deformation (in-plane)**		**Ring deformation (out-of-plane)**	
**[Ni(phen)(L1)**_**2**_**]**	338	1579, 1459		1021		724	
**[Ni(phen)(L2)**_**2**_**]**	350	1580, 1455		1013		724	

**Fig. 2:**
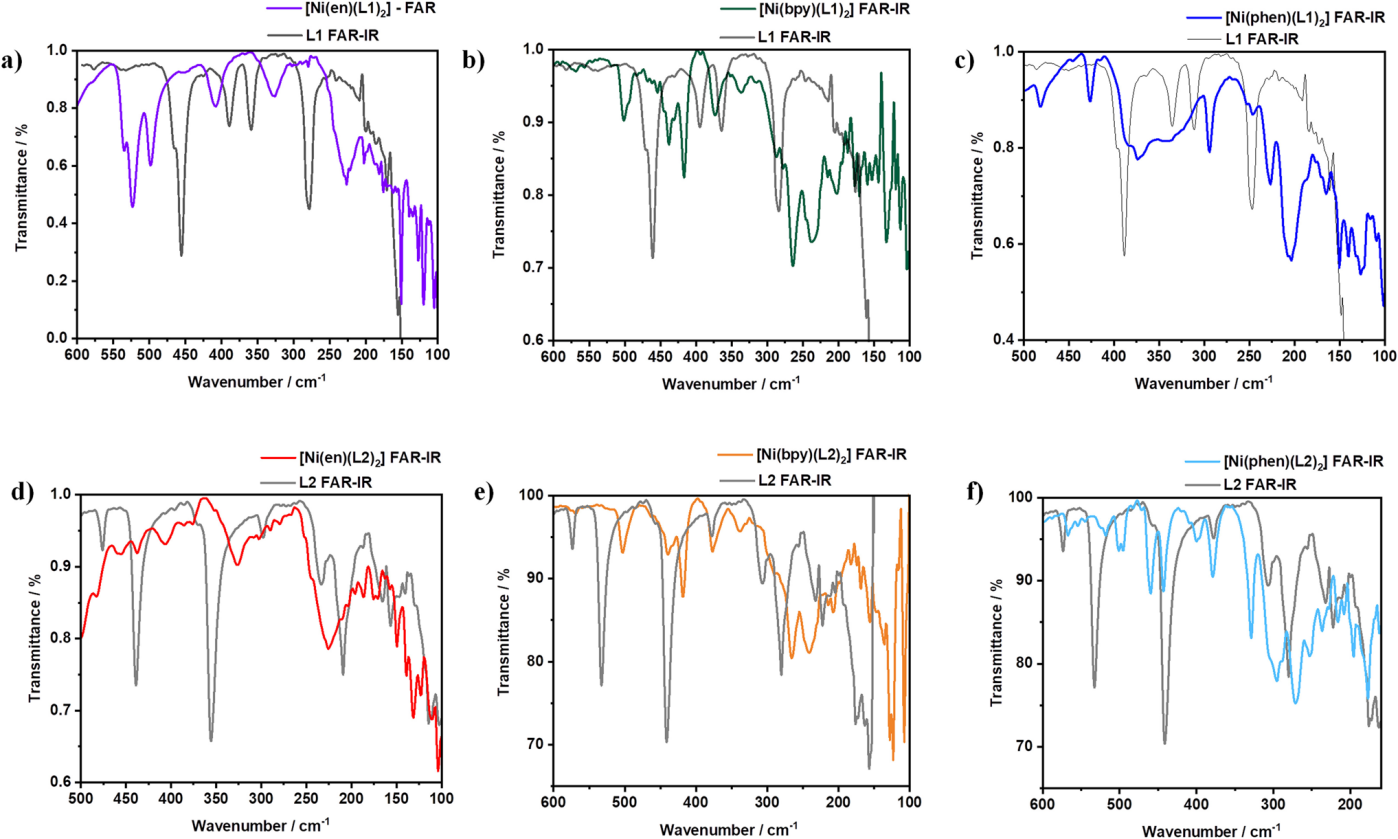
Far-IR spectra of the following: [Ni(en)(L1)_2_] (**a**); [Ni(bpy)(L1)_2_] (**b**); [Ni(phen)(L1)_2_] (**c**); [Ni(en)(L2)_2_] (**d**); [Ni(bpy)(L2)_2_] (**e**); [Ni(phen)(L2)_2_] (**f**), compared with the corresponding xanthate salts (L1 and L2)

In addition to mid-infrared (4000–500 cm^−1^) analysis, the synthesized complexes were characterized by far-infrared spectroscopy (500–100 cm^−1^). The spectra obtained in this range allowed the assignment of signals associated with the angular deformations δC–O–C and δS–C–S. Figure 2 shows the far-IR spectra of the complexes in comparison with those of the xanthate salts. For the ligands, absorptions around 534 and 279 cm^−1^ were assigned to the δC–O–C and δS–C–S vibrational modes, respectively. In the complexes, the corresponding absorptions were observed at approximately 498 and 391 cm^−1^, with a slight shift to lower wavenumbers. In addition to the assignments related to the angular deformations of the xanthate ligands, the far-IR region also exhibits stretching vibrations associated with metal–ligand bonds. Table 1 illustrates the wavenumbers associated with the υNi–S and υNi–N bands, observed in the ranges of 342–414 cm^−1^ and 428–504 cm^−1^, respectively, in agreement with values reported in the literature [15, 47]. The presence of these vibrational modes indicates the coordination of the xanthate and nitrogen-containing ligands to the nickel center, supporting the formation of Ni–S and Ni–N bonds in the complexes.

As a complementary analysis to the infrared spectra, Raman spectra were obtained (Figure S3). However, for the complexes containing the aromatic ligands bpy and phen, it was not possible to obtain spectra covering the entire spectral region (4000–100 cm^−1^) with sufficient resolution for the assignment of all bands. For the [Ni(en)(L1)_2_] and [Ni(en)(L2)_2_] complexes, bands assigned to the stretching vibrations υ_coupled_C–O, υ_ass_C–S, υC–O, υ_s_C–S, υNi–S and υNi–N were observed at approximately 1273, 1020, 992, 651, 278, and 326 cm^−1^, and 1145, 1019, 990, 651, 280, and 326 cm^−1^, respectively. In addition, angular deformation modes δC–O–C and δS–C–S were identified at 497 and 402 cm^−1^ for the complex containing *n*-butyl xanthate, and at 481 and 408 cm^−1^ for the complex containing 2-methoxyethyl xanthate.

**Fig. 3:**
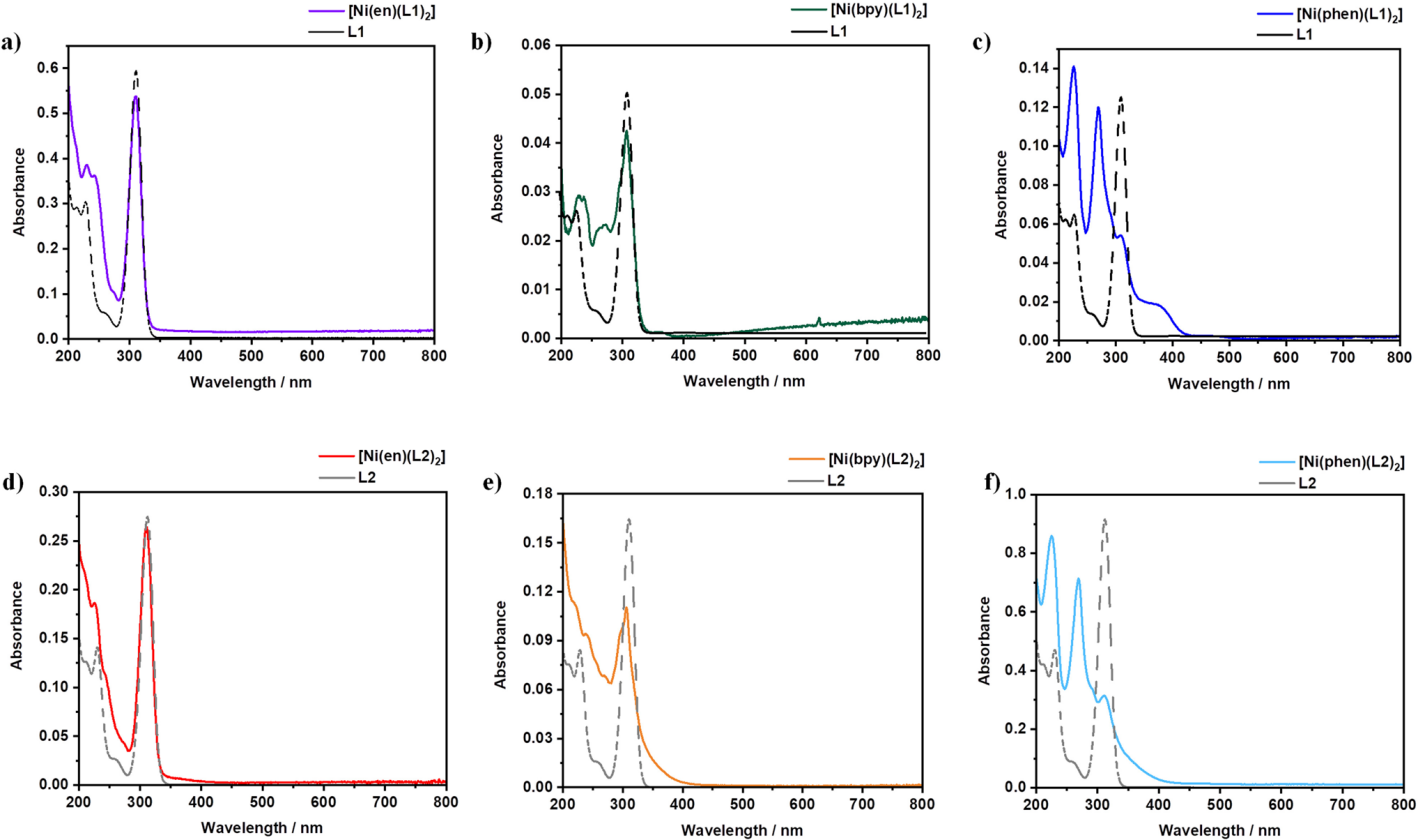
UV–Vis spectra of the following: [Ni(en)(L1)_2_] (**a**); [Ni(bpy)(L1)_2_] (**b**); [Ni(phen)(L1)_2_] (**c**); [Ni(en)(L2)_2_] (**d**); [Ni(bpy)(L2)_2_] (**e**); [Ni(phen)(L2)_2_] (**f**), compared with the corresponding xanthate salts (L1 and L2)

In addition to vibrational spectroscopy, the complexes were characterized by UV–Vis electronic spectroscopy. Figure S4 shows the UV–Vis spectra of the investigated complexes in solution (acetonitrile, 10^–5^ M). Due to band overlap, spectral deconvolution was performed, allowing the identification of bands associated with electronic transitions. The absorbance data and molar absorptivity values for each complex were determined and are presented in Table S1 of the Supporting Information. Furthermore, a comparison between the spectra of the complexes and the xanthate ligands was carried out (Fig. 3). The spectra of the xanthate salts exhibit two characteristic bands assigned to n → π* and π → π* electronic transitions, located at wavelengths of approximately 220 and 310 nm, respectively [13]. Through the deconvolution of the electronic spectra of the complexes (Figure S4), it is possible to observe bands corresponding to these intraligand electronic transitions associated with the xanthate ligands, corroborating the presence of these species in the complexes. However, a comparison of the band profiles of the xanthate salts and the complexes reveals that those containing the ethylenediamine ligand show higher correspondence, suggesting that these systems may exhibit a low contribution from electronic transitions involving the nitrogen ligand. In addition, complexes containing bpy and phen display more pronounced differences, indicating a stronger contribution from other electronic transitions, which are discussed in greater detail in the “UV − Vis electronic analysis” section.

### Thermochemical analysis

**Fig. 4:**
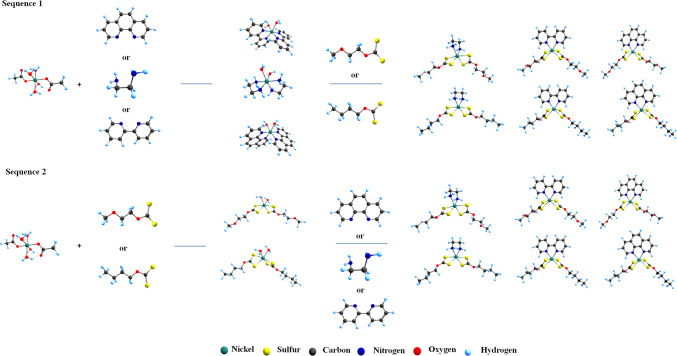
Optimized structures of all reactants and intermediates involved in the two thermochemical sequences considered

To understand the formation trends of heteroleptic complexes containing xanthate and nitrogen-donor ligands, the values of ΔG, ΔH, and ΔS were analyzed using CPCM (ethanol) (Tables S4 and S5). Since the literature reports two possible sequences for the addition of nitrogen-donor and xanthate ligands to the metal center, both coordination sequences were considered in this study (Fig. 4) [30, 48]. In the first, the nitrogen-donor ligand was assumed to coordinate initially to nickel, followed by the addition of the xanthate ligands, whereas in the second, the reverse order was considered. For both sequences, the thermochemical cycles were modeled using nickel(II) acetate tetrahydrate as the experimental precursor, leading to the formation of intermediate species employed in the thermodynamic analysis. The choice of the structure of Ni(CH_3_COO)_2_.4H_2_O was based on the literature, whose crystallographic data indicate the coordination of four water molecules and monodentate acetate groups to the metal center [49]. In the sequence in which the nitrogen-donor ligand is added first to the precursor Ni(CH_3_COO)_2_.4H_2_O, the ΔG and ΔH values were obtained from the difference between the thermodynamic parameters (Gibbs free energy and enthalpy) of the intermediates [Ni(en)_2_(H_2_O)_2_]^2+^, [Ni(bpy)_2_(H_2_O)_2_]^2+^, and [Ni(phen)_2_(H_2_O)_2_]^2+^ and the corresponding final heteroleptic complexes (Table S6). These intermediates arise from the prior interaction between Ni(CH_3_COO)_2_.4H_2_O and each nitrogen-donor ligand (en, bpy, or phen), preceding the addition of the xanthate ligand. Similarly, the analysis of the sequence in which the xanthate ligand was added first was carried out based on the thermodynamic parameters of the intermediates [Ni(L1)_2_(H_2_O)_2_] and [Ni(L2)_2_(H_2_O)_2_].

The choice to analyze the thermodynamic parameters based on possible intermediates was due to experimental observations. During the synthesis process of sequence 1, a change in the color of the initially green nickel acetate solution is observed after the addition of the nitrogen ligand. However, no precipitation of the material occurs. Therefore, the values of ΔG, ΔH, and ΔS were also calculated for the intermediates, as illustrated in Tables S4 and S5. For sequence 1, the formation of intermediate complexes prior to the heteroleptic xanthate complexes [Ni(en)_2_(H_2_O)_2_]^2+^, [Ni(bpy)_2_(H_2_O)_2_]^2+^, and [Ni(phen)_2_(H_2_O)_2_]^2+^ is proposed. Equations 1–3 illustrate the calculation of ΔG for these systems. ΔH values were calculated analogously to ΔG. The entropic contribution was reported as TΔS and calculated from TΔS = ΔH − ΔG.

1 $${\Delta G}_{[\mathrm{N}\mathrm{i}{\left(\mathrm{e}\mathrm{n}\right)}_{2}({H}_{2}\mathrm{O}{)}_{2}{]}^{2+}}={(G}_{[\mathrm{N}\mathrm{i}{\left(\mathrm{e}\mathrm{n}\right)}_{2}({H}_{2}\mathrm{O}{)}_{2}{]}^{2+ }+ }{2 G}_{\mathrm{A}\mathrm{c}\mathrm{e}\mathrm{t}\mathrm{a}\mathrm{t}\mathrm{e}}+{2G}_{{H}_{2}O})-({G}_{\left[\mathrm{N}\mathrm{i}{\left(C{H}_{3}COO\right)}_{2}4{H}_{2}\mathrm{O}\right]}+2{G}_{\mathrm{e}\mathrm{n}})$$

2 $${\Delta G}_{[\mathrm{N}\mathrm{i}{\left(bpy\right)}_{2}({H}_{2}\mathrm{O}{)}_{2}{]}^{2+}}={(G}_{[\mathrm{N}\mathrm{i}{\left(bpy\right)}_{2}({H}_{2}\mathrm{O}{)}_{2}{]}^{2+ }+ }{2 G}_{\mathrm{A}\mathrm{c}\mathrm{e}\mathrm{t}\mathrm{a}\mathrm{t}\mathrm{e}}+{2G}_{{H}_{2}O})-({G}_{\left[\mathrm{N}\mathrm{i}{\left(C{H}_{3}COO\right)}_{2}4{H}_{2}\mathrm{O}\right]}+2{G}_{bpy})$$

3 $${\Delta G}_{[\mathrm{N}\mathrm{i}{\left(\mathrm{p}\mathrm{h}\mathrm{e}\mathrm{n}\right)}_{2}({H}_{2}\mathrm{O}{)}_{2}{]}^{2+}}={(G}_{[\mathrm{N}\mathrm{i}{\left(\mathrm{p}\mathrm{h}\mathrm{e}\mathrm{n}\right)}_{2}({H}_{2}\mathrm{O}{)}_{2}{]}^{2+ }+ }{2 G}_{\mathrm{A}\mathrm{c}\mathrm{e}\mathrm{t}\mathrm{a}\mathrm{t}\mathrm{e}}+{2G}_{{H}_{2}O})-({G}_{\left[\mathrm{N}\mathrm{i}{\left(C{H}_{3}COO\right)}_{2}4{H}_{2}\mathrm{O}\right]}+2{G}_{\mathrm{p}\mathrm{h}\mathrm{e}\mathrm{n}})$$

The values of ΔG and ΔH indicate that the formation of these complexes is thermodynamically unfavorable, being an endothermic process, which suggests an energetic cost associated with structural reorganization (Table S4). However, the TΔS values show that they have a favorable entropic contribution, but that it does not compensate for the enthalpic term. These data agree with experimental observations, since no precipitation of any intermediate occurs after the addition of the nitrogen ligand to the nickel acetate solution. Although a color change is observed, suggesting the formation of coordination species in solution, the positive ΔG values obtained under CPCM solvation conditions indicate that these intermediates are not thermodynamically favored as stable species. For comparison purposes, the formation of an intermediate in the synthesis process in which the xanthate ligand is added first (sequence 2) was also considered. For this sequence, the thermodynamic parameters of the intermediate complexes [Ni(L1)_2_(H_2_O)_2_] and [Ni(L2)_2_(H_2_O)_2_] were calculated. Equations 4 and 5 illustrate the calculation of ΔG for these complexes, which was also applied to obtain the ΔH values. As observed for sequence 1, the formation of these intermediate complexes is also thermodynamically unfavorable and endothermic (Table S5).

4 $${\Delta G}_{[Ni(L{1)}_{2}({H}_{2}O{)}_{2}]}=\left({G}_{[Ni(L{1)}_{2}({H}_{2}O{)}_{2}]}+ 2{G}_{\mathrm{A}\mathrm{c}\mathrm{e}\mathrm{t}\mathrm{a}\mathrm{t}\mathrm{e}}+2{G}_{{H}_{2O}}\right)-({G}_{\left[\mathrm{N}\mathrm{i}{\left(C{H}_{3}COO\right)}_{2}4{H}_{2}\mathrm{O}\right]}+{G}_{L1})$$

5 $${\Delta G}_{[Ni(L{2)}_{2}({H}_{2}O{)}_{2}]}=\left({G}_{[Ni(L{2)}_{2}({H}_{2}O{)}_{2}]}+ 2{G}_{\mathrm{A}\mathrm{c}\mathrm{e}\mathrm{t}\mathrm{a}\mathrm{t}\mathrm{e}}+2{G}_{{H}_{2O}}\right)-({G}_{\left[\mathrm{N}\mathrm{i}{\left(C{H}_{3}COO\right)}_{2}4{H}_{2}\mathrm{O}\right]}+{G}_{L2})$$

**Fig. 5:**
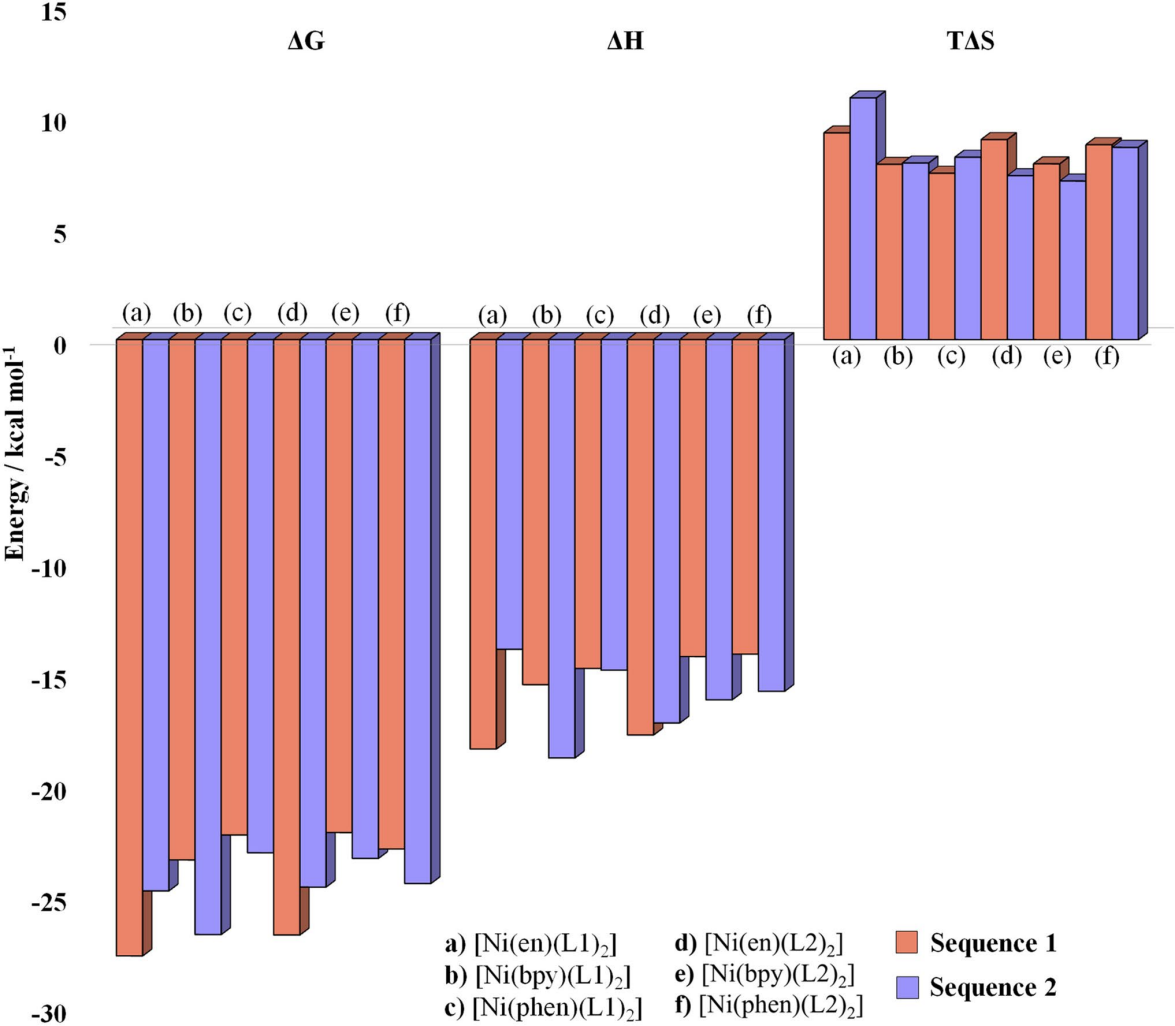
Thermochemical parameters (ΔG, ΔH, and TΔS) calculated for the two ligand-addition sequences. For each parameter, bars correspond to the six heteroleptic complexes in the following order: [Ni(en)(L1)_2_], [Ni(bpy)(L1)_2_], [Ni(phen)(L1)_2_], [Ni(en)(L2)_2_], [Ni(bpy)(L2)_2_], and [Ni(phen)(L2)_2_]. Sequence 1 corresponds to prior coordination of the N-donor ligand, followed by xanthate addition; sequence 2 corresponds to prior xanthate coordination, followed by N-donor ligand addition

In contrast to the intermediates, the results show that both sequences lead to thermodynamically favorable and exothermic formation of the final complexes. A comparison between the results obtained for sequence 1 and sequence 2 (Fig. 5) indicates that both coordination sequences lead to thermodynamically comparable heteroleptic complexes. Equations 6–11 show the calculations performed for sequence 1 (Eqs. 6–8) and sequence 2 (Eqs. 9–11). For simplicity, the xanthate ligands were expressed in a generic form as LX (X = 1 or 2).

6 $${\Delta G}_{[Ni(\mathrm{e}\mathrm{n})(L{X)}_{2}]}=\left({G}_{[Ni(\mathrm{e}\mathrm{n})(L{X)}_{2}]}+ {G}_{\mathrm{e}\mathrm{n}}+2{G}_{{H}_{2O}}\right)-({G}_{[Ni(\mathrm{e}\mathrm{n}{)}_{2}({H}_{2}O{)}_{2}]}+{2G}_{LX})$$

7 $${\Delta G}_{[Ni(bpy)(L{X)}_{2}]}=\left({G}_{[Ni(bpy)(L{X)}_{2}]}+ {G}_{bpy}+2{G}_{{H}_{2O}}\right)-({G}_{[Ni(bpy{)}_{2}({H}_{2}O{)}_{2}]}+{2G}_{LX})$$

8 $${\Delta G}_{[Ni(\mathrm{p}\mathrm{h}\mathrm{e}\mathrm{n})(L{X)}_{2}]}=\left({G}_{[Ni(\mathrm{p}\mathrm{h}\mathrm{e}\mathrm{n})(L{X)}_{2}]}+ {G}_{\mathrm{p}\mathrm{h}\mathrm{e}\mathrm{n}}+2{G}_{{H}_{2O}}\right)-({G}_{[Ni(\mathrm{p}\mathrm{h}\mathrm{e}\mathrm{n}{)}_{2}({H}_{2}O{)}_{2}]}+{2G}_{LX})$$

9 $${\Delta G}_{[Ni(\mathrm{e}\mathrm{n})(L{X)}_{2}]}=\left({G}_{[Ni(\mathrm{e}\mathrm{n})(L{X)}_{2}]}+ 2{G}_{{H}_{2O}}\right)-({G}_{[Ni(LX{)}_{2}({H}_{2}O{)}_{2}]}+{G}_{\mathrm{e}\mathrm{n}})$$

10 $${\Delta G}_{[Ni(bpy)(L{X)}_{2}]}=\left({G}_{[Ni(bpy)(L{X)}_{2}]}+ 2{G}_{{H}_{2O}}\right)-({G}_{[Ni(LX{)}_{2}({H}_{2}O{)}_{2}]}+{G}_{bpy})$$

11 $${\Delta G}_{[Ni(\mathrm{p}\mathrm{h}\mathrm{e}\mathrm{n})(L{X)}_{2}]}=\left({G}_{[Ni(\mathrm{p}\mathrm{h}\mathrm{e}\mathrm{n})(L{X)}_{2}]}+ 2{G}_{{H}_{2O}}\right)-({G}_{[Ni(LX{)}_{2}({H}_{2}O{)}_{2}]}+{G}_{\mathrm{p}\mathrm{h}\mathrm{e}\mathrm{n}})$$

Although sequence 1, in which the nitrogen-based ligand coordinates first to the metal center followed by the xanthate ligand, shows slightly more favorable Gibbs free energies for ethylenediamine systems, the differences are generally small, and no consistent trend is observed across all complexes. These findings suggest that the thermodynamic preference for complex formation depends on the ligand environment rather than on a specific coordination order. This result supports the formation of the complexes through both ligand addition orders, as reported in the literature [30, 48].

As the experimental data were obtained through sequence 1, the discussion of the differences between the complexes was based on the values calculated according to this ligand addition order. A comparison of the heteroleptic xanthate complexes containing the en, bpy, and phen ligands (sequence 1) showed that the ΔG values for the complexes containing the ethylenediamine ligand ([Ni(en)(L1)_2_] and [Ni(en)(L2)_2_]) were approximately 3.86 to 5.43 kcal mol^−1^ more negative than the ΔG values for the complexes containing aromatic ligands. This small difference was associated with the less electronically complex structure of ethylenediamine, which results in lower steric hindrance. This difference between the complexes containing the en ligand and those containing bpy and phen was not higher because the aromatic ligands bpy and phen can compensate through their π-acceptor character. Both bpy and phen possess low-energy π* orbitals, favoring metal-to-ligand charge transfer interactions [50]. As a result, these electronic effects help balance steric factors, leading to only small differences in the thermochemical parameters among the complexes. In addition, small differences in ΔS values were observed among the heteroleptic complexes containing the en, bpy, and phen ligands. Table S4 shows that the entropic term (TΔS) ranged from 7.47 to 9.28 kcal mol^−1^, evidencing only small variations in the entropic contribution among the investigated systems. Therefore, although entropic contributions are relevant to the stability of all complexes, variations in entropy do not constitute the primary factor responsible for the thermodynamic differences observed between them, since the favorable ΔH values and their variations among the complexes suggest that the relative stabilization of the system is predominantly governed by enthalpic effects.

**Fig. 6:**
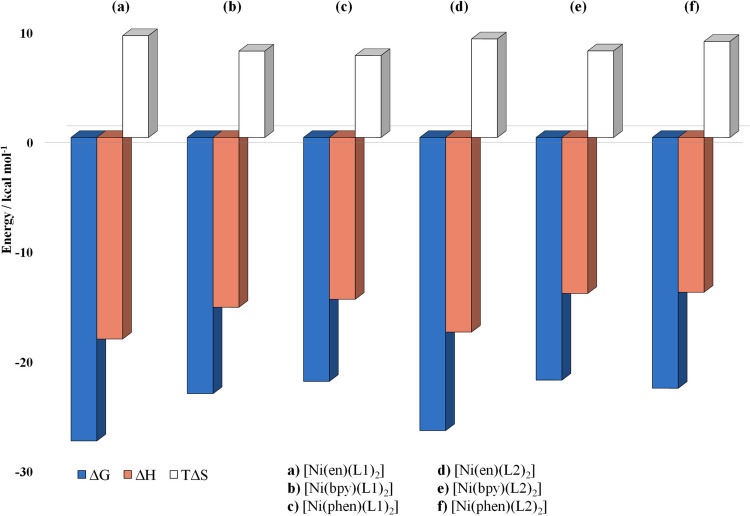
Thermochemical parameters (ΔG, ΔH, and TΔS) calculated for the heteroleptic complexes using sequence 1

In addition to the analysis varying the nitrogen ligand, a comparison was also made between complexes containing ligands L1 and L2. The results indicate that the substitution of L1 by L2 has a minimal effect on the thermodynamic parameters, with variations of approximately 1 kcal mol^−1^ in the ΔG values between the systems, with slightly more favorable values observed for the *n*-butyl containing complexes (L1). This slight difference in ΔG values between the systems is due to the presence of the oxygen heteroatom in the 2-methoxyethyl xanthate ligand chain. Due to its higher electronegativity, the oxygen partially reduces the electron density of the coordinating sulfur, resulting in less effective coordination to the metal center compared to the *n*-butyl xanthate ligand. However, this effect is moderate, as evidenced by the enthalpy values (ΔH), which show only minimal decreases (below 1 kcal mol^−1^). This indicates that, although the oxygen atom presents an electron-withdrawing inductive effect, the Ni–S bond strength is preserved, resulting in very similar thermodynamic stability (ΔG) for both systems. Figure 6 illustrates the ΔG, ΔH, and TΔS data for the heteroleptic complexes calculated using sequence 1.

###  UV − Vis electronic analysis

**Fig. 7:**
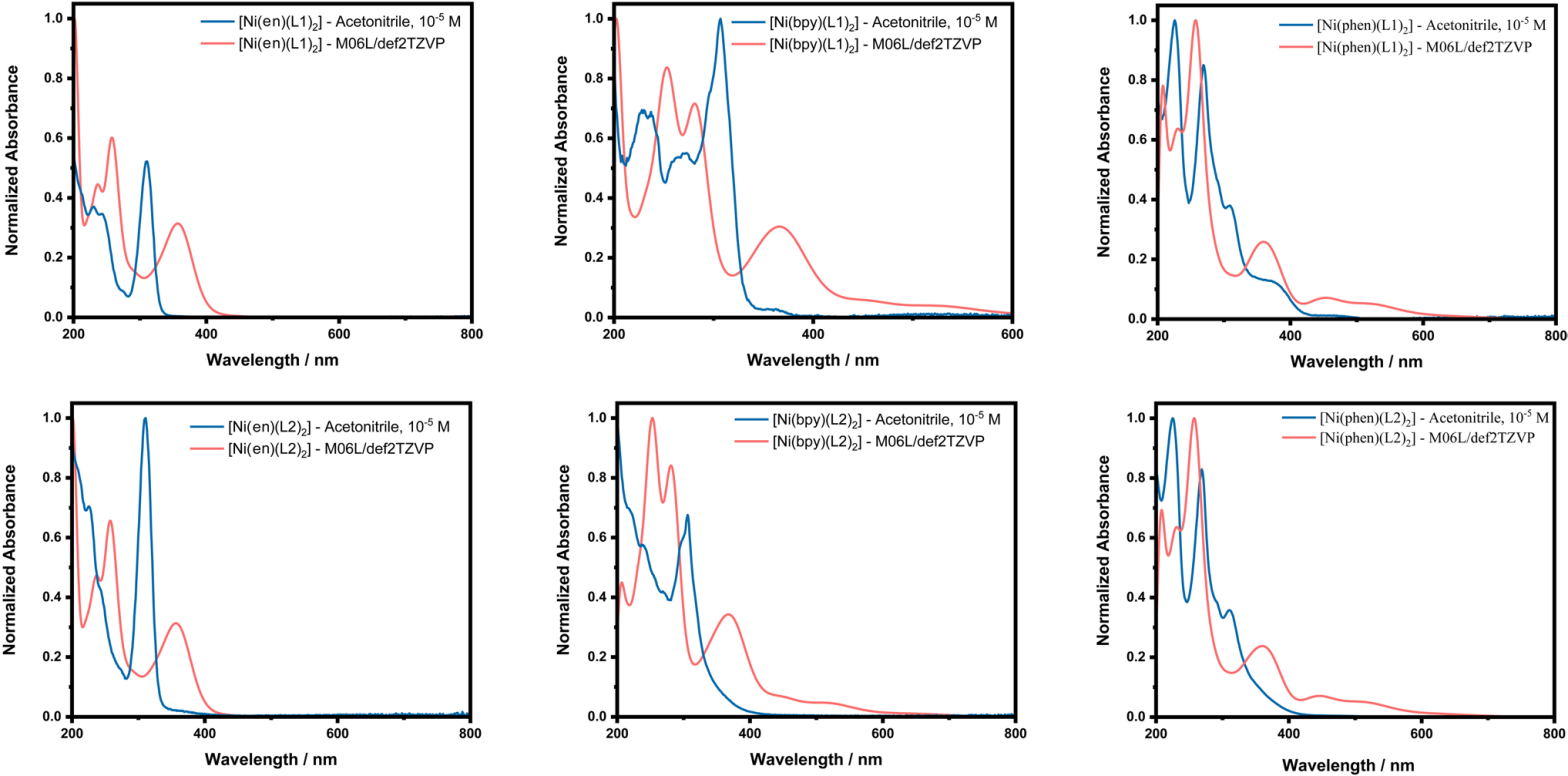
Experimental (blue) and TD-DFT calculated (red, M06L/def2-TZVP) normalized UV–Vis spectra of Ni(II) xanthate complexes in acetonitrile

The six synthesized heteroleptic complexes were characterized by UV–Vis spectroscopy in solution. Figures S4 and S5 show the spectra obtained in solution (acetonitrile), in concentrations of 10^–5^ and 10^–3^ M, respectively. In addition, a comparative analysis was performed between the experimental results and the theoretical data of electronic transitions, obtained by TD-DFT calculations, thereby providing a clearer interpretation of the excited states involved in these systems (Fig. 7).

**Fig. 8:**
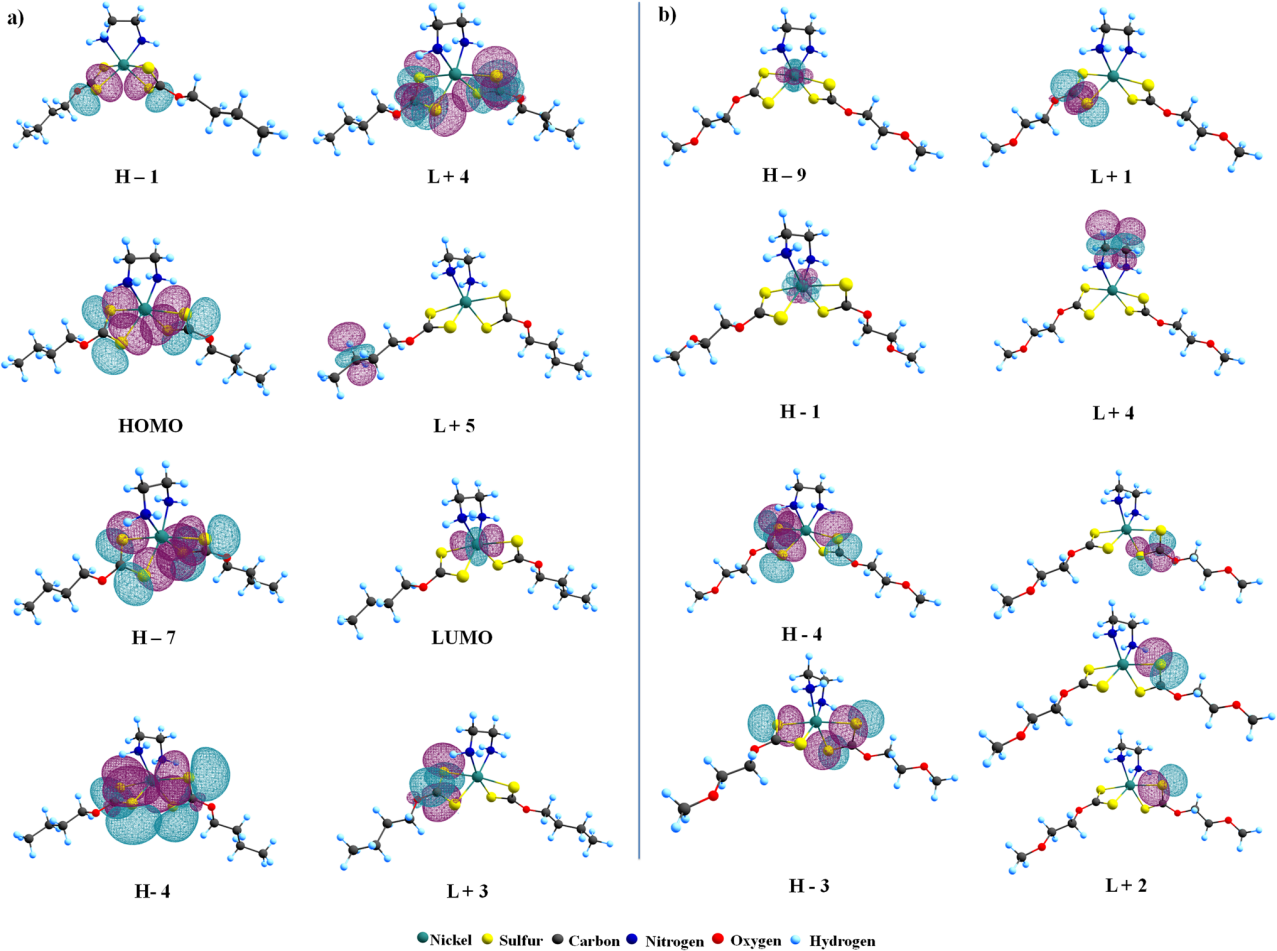
Selected molecular orbitals involved in the electronic transitions of the [Ni(en)(L1)_2_] (**a**) and [Ni(en)(L2)_2_] (**b**) complexes. Isosurface = 0.04 a.u

Tables S7–S15 summarize the main UV–Vis absorption bands, their assignments based on TD-DFT calculations, and the molecular orbital energies obtained from NBO analysis for the investigated complexes. These tables also provide the electronic transitions associated with each absorption band and the corresponding orbital character. Table S7 summarizes the molecular orbitals involved in the electronic transitions associated with each absorption band observed in the experimental spectra of the [Ni(en)(L1)_2_] and [Ni(en)(L2)_2_] complexes. The results show a good correlation between the experimental data and the TD-DFT calculations, with energy deviations in the range of 0.04–0.37 eV for the complexes containing ligand L1 and 0.00–0.99 eV for those containing ligand L2. In all cases, bands around 225 and 300 nm are observed, corresponding to n → π and π → π* electronic transitions associated with the xanthate ligand fragments, which is consistent with the presence of xanthate ligands in the complexes [51]. However, the TD-DFT and NBO analyses reveal that these absorptions are not purely localized intraligand excitations but rather involve a mixture of intraligand and charge transfer contributions, with multiconfigurational character, as indicated by the configuration interaction coefficients. The natural bond orbital (NBO) analysis, performed from canonical molecular orbitals (CMO), allows the identification of the orbitals involved in the electronic transitions, as illustrated in Fig. 8. The selected orbitals were decomposed into percentage contributions of the corresponding fragments, as presented in Tables S8 and S9 for the complexes containing the en ligand. For the experimental spectral band at 228 nm of the [Ni(en)(L1)_2_] complex, it was observed that the occupied molecular orbitals involved in these transitions are predominantly sulfur-based lone pair orbitals, indicating a strong donor character of the xanthate ligand. In contrast, the corresponding unoccupied orbitals display antibonding character delocalized over the ligand framework, particularly associated with C–S and C–H bonds. Although the TD-DFT and NBO results indicate that these absorptions are not purely intraligand in nature, the high energy band at 228 nm is predominantly ligand-centered, being mainly governed by intraligand transitions within the xanthate fragment. The minor contribution observed for this band involves participation of the nickel center (Tables S7 and S8). This difference relative to the isolated ligand indicates electronic restructuring after complex formation, since contributions from the metal center are observed in electronic transitions, even as a secondary component. This result indicates partial metal–ligand orbital mixing in excited states, arising from the energetic proximity and symmetry compatibility between the nickel orbitals and the ligand-based orbitals. The mixing of metal and ligand orbitals becomes more pronounced in the lower-energy band observed at 310 nm in the spectrum of [Ni(en)(L1)_2_]. In this case, the intraligand electronic transition character becomes minor, while ligand-to-metal charge-transfer (LMCT) becomes predominant, involving electron donation from the sulfur lone pair orbitals of the xanthate to the nickel metal center, as indicated by the TD-DFT and NBO analyses. The variation in the predominance of the electronic transition character observed for the bands at 228 and 310 nm arises from differences in the energetic stabilization associated with the nature of the virtual orbitals involved in metal–ligand interactions. The predominantly LMCT character of the 310 nm band is consistent with the lower energy of the LUMO (−2.00 eV), which favors stronger interaction with the nickel orbitals, resulting in more pronounced metal–ligand orbital mixing. In contrast, the LUMO + 4 orbital involved in the 228 nm transition has higher energy (0.869 eV), is less stabilized, and therefore remains predominantly ligand centered. In addition to the bands directly associated with the xanthate ligand fragments, other bands are observed in the spectrum of [Ni(en)(L1)_2_] that are characteristic of complex formation. The band at 274 nm, for example, shows a predominant contribution from the metal center in the occupied orbital, while the virtual orbital is centered on fragments of the xanthate ligand. Therefore, this band can be assigned to a metal to ligand charge transfer (MLCT) transition. The electronic transition observed at 274 nm arises from the nature and relative energies of the orbitals involved. In this case, the occupied orbital has an energy of −5.93 eV, centered on the metal, whereas the virtual orbital has a relatively low energy (−1.37 eV) and is predominantly localized on the xanthate ligand. When compared to the bands at 228 and 310 nm, which exhibit mixed intraligand and LMCT character, the energy gap between the occupied and virtual orbitals at 274 nm is smaller, favoring electron transfer from the nickel center to the ligand (Tables S7 and S8). In addition, the bands located in the higher energy UV region (180, 215, and 244 nm) exhibit a more pronounced multiconfigurational character, appearing as superpositions of multiple electronic transitions. This effect arises both from the complexity of the electronic coupling in the system and from the limitations of the functional used in the TD-DFT calculations.

Similarly, the same analysis was performed for the orbitals involved in the electronic transitions associated with the absorption bands at 229 and 310 nm in the experimental spectrum of the [Ni(en)(L2)_2_] complex. In both cases, the absence of a single dominant contribution indicates that these bands originate from excited states with multiconfigurational character. Differently from what was observed for the [Ni(en)(L1)_2_] complex, the absorption band at 229 nm in the complex containing ligand L2 is predominantly associated with a metal to ligand charge transfer (MLCT) transition, mainly involving the xanthate ligand, as illustrated in Fig. 8b. However, electronic transitions between ligand-centered orbitals are also observed as minor contributions. This result indicates that the xanthate substituent (*n*-butyl and 2-methoxyethyl) has a significant influence on the nature of the electronic transitions in the heteroleptic complexes, particularly in the assignment of LMCT and MLCT character. An analysis of the xanthate ligand structure shows that the *n*-butyl (L1) chain contains only an alkyl group, which provides a relatively weak electron-donating inductive effect. As a result, the sulfur atom retains a high electron density, favoring LMCT type transitions. In contrast, the 2-methoxyethyl (L2) substituent contains an oxygen heteroatom in the chain, which introduces an electron-withdrawing effect. Consequently, the electron density at the sulfur atom is reduced, weakening its donor character. As a result, LMCT is less favored, and metal to ligand charge transfer (MLCT) transitions become more significant.

**Fig. 9:**
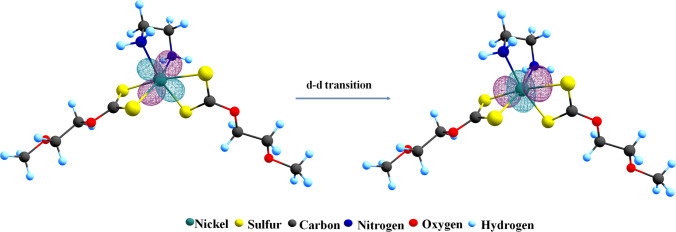
Natural bond orbital (NBO) analysis showing the molecular orbitals involved in the d-d transition of the [Ni(en)(L2)_2_] complex. Isosurface = 0.04 a.u

In addition to these electronic transitions, the complexes also exhibit excitations involving the nitrogen atoms of the ethylenediamine ligand. However, the transitions observed in the UV–Vis spectrum are predominantly centered on the orbitals of the nickel(II) center and on the more polarizable atoms, such as sulfur and oxygen. The predominance of sulfur and oxygen atoms observed in the spectra of heteroleptic complexes is attributed to the high polarizability of the conjugated π-system present in the xanthate. This increases the probability of charge-transfer transitions involving the xanthate group, resulting in higher intensities of electronic transitions compared to transitions involving the more localized bonds of the nitrogen-donor ligand. Consequently, electronic transitions involving these atomic orbitals, such as charge transfers, exhibit a higher probability of occurrence and are observed with higher intensity in the spectrum. The NBO analysis corroborates that the orbitals localized on the nitrogen atoms contribute only secondarily to the molecular orbitals involved in the electronic excitations. This can be observed through the occupied orbital of the [Ni(en)(L1)_2_] complex for the band at 228 nm, in which the nitrogen orbitals account for approximately 26% of the total orbital composition (Table S8). For the [Ni(en)(L2)_2_] complex, it was possible to observe a band at 364 nm that is not present in the analog with the *n*-butyl xanthate ligand (L1). The analysis reveals that this band arises from electronic transitions from occupied orbitals with xanthate and metal contributions to a virtual orbital predominantly centered on the nickel atom. This indicates the d-d character of the electronic transition in the [Ni(en)(L2)_2_] complex, corroborating the higher mixing between ligand and metal orbitals in the 2-methoxyethyl xanthate complex (Fig. 9).

The same analysis was performed for the complexes containing the bpy and phen ligands (Tables S10 and S13). The TD-DFT analysis also showed good agreement with the experimental data, with energy differences below 1 eV. Compared to complexes containing the ethylenediamine ligand, systems containing aromatic ligands exhibit a more pronounced multiconfigurational character, which is associated with the presence of low-energy π* orbitals and stronger electronic coupling between the nickel d orbitals and the ligand orbitals [52–54]. The complexes [Ni(bpy)(L1)_2_] and [Ni(bpy)(L2)_2_] also exhibited the characteristic xanthate bands, observed at 224 and 307 nm for the first complex and at 219 and 307 nm for the second. As in the analyzed heteroleptic complexes with ethylenediamine, these transitions correspond predominantly to intraligand electronic transitions between fragments of the xanthate molecules (Tables S11 and S12). The orbitals involved in the main electronic transitions for [Ni(bpy)(L1)_2_] and [Ni(bpy)(L2)_2_] complexes are shown in Figure S8. Unlike the en complexes, significantly higher contributions from the nitrogen ligand were observed in these systems. In the [Ni(bpy)(L1)_2_] complex, for example, the bpy fragment contributes predominantly through antibonding C–N and C–C orbitals, together with smaller contributions from the xanthate ligands (Table S11).

In addition to the characteristic bands around 225 and 300 nm, the [Ni(bpy)(L1)_2_] complex exhibited three additional bands in the experimental spectrum at 258, 290, and 343 nm, which were not observed for the [Ni(en)(L1)_2_] complex. Although these bands suggest multiconfigurational excited states, a more general analysis shows that they arise from a combination of metal–ligand charge transfer and intraligand transitions, reflecting stronger electronic coupling between the nickel center and the π-system of the bpy ligand. For the [Ni(bpy)(L2)_2_] complex, additional bands were observed that were not present in the experimental spectrum of the analog containing the en ligand. Therefore, the experimental bands at 238, 266, 292, and 305 nm were investigated using TD-DFT calculations in order to identify the corresponding theoretical electronic transitions (Tables S10). The higher energy bands at 238 and 266 nm exhibited a mixture of metal–ligand and intraligand electronic transitions, involving both the xanthate ligands and the bpy ligand (Table S10). However, the bands at 292 and 305 nm correspond to electronic transitions involving only the bpy ligand and the metal center (Table S10). This metal to ligand charge transfer (MLCT) electronic transition is expected for bpy-containing complexes, due to the presence of low-energy π* orbitals capable of stabilizing metal centers through MLCT interactions, as previously mentioned in the thermochemical analysis section. Figure S9 illustrates the orbitals involved in the main electronic transitions associated with the 292 nm band in the experimental spectrum of the [Ni(bpy)(L2)_2_] complex.

**Fig. 10:**
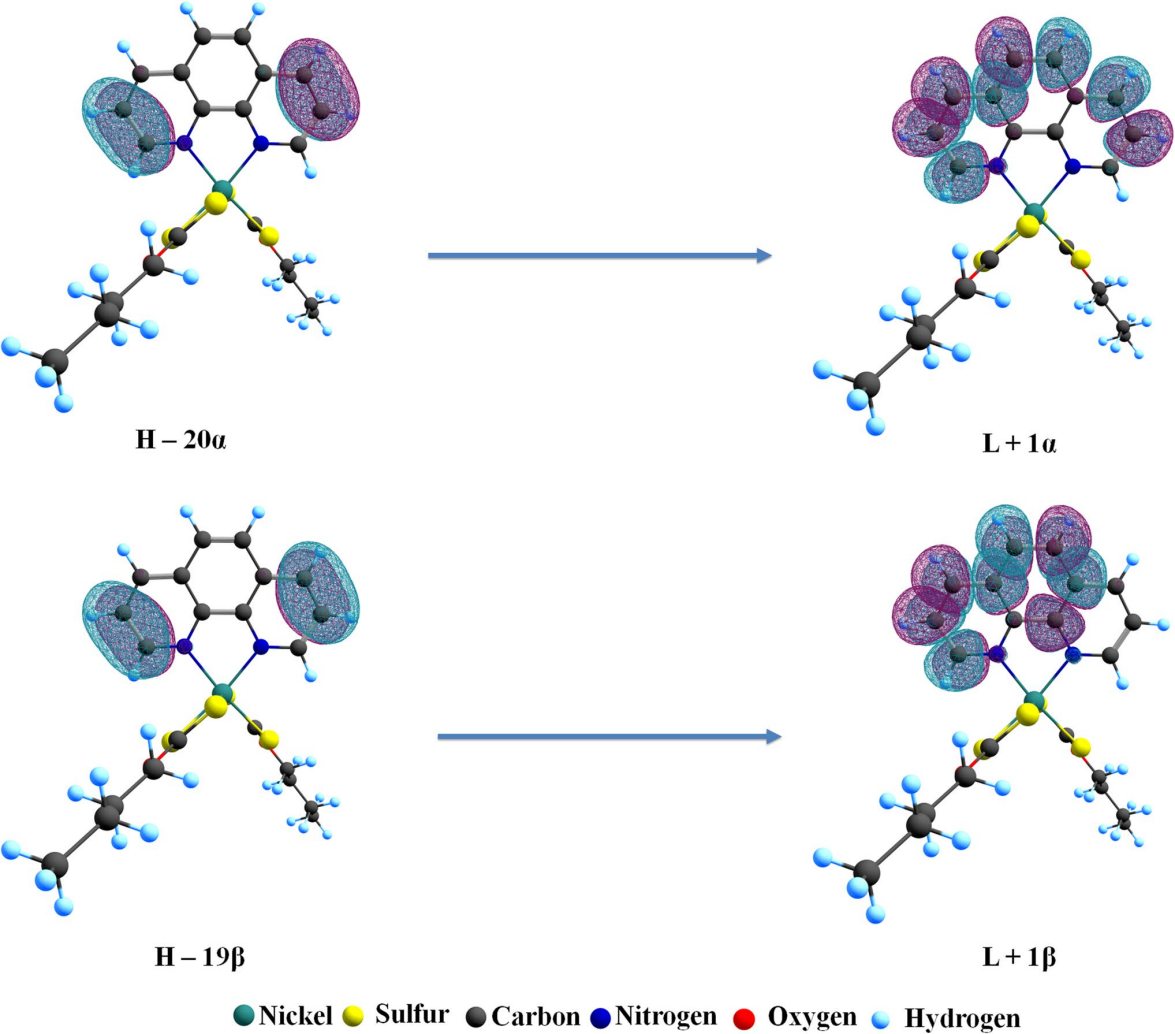
Natural bond orbital (NBO) analysis showing the molecular orbitals involved in the intraligand transition of the [Ni(phen)(L1)_2_] complex. Isosurface = 0.04 a.u

The final set analyzed comprises the phen complexes, [Ni(phen)(L1)_2_] and [Ni(phen)(L2)_2_]. The TD-DFT results presented in Table S13 show that these systems exhibit a stronger multiconfigurational character than the complexes with the en and bpy ligands. This is reflected in the closer energetic proximity between the low-lying excited states. The higher π conjugation and structural rigidity of phen lead to a stronger stabilization of the ligand π* orbitals, favoring excited states of predominantly metal–ligand charge transfer (MLCT) character. This trend is observed, for example, in the assignment of the experimental band at 225 nm. In the complexes with the en and bpy ligands, this band is mainly associated with electronic transitions involving the xanthate ligands. However, in the [Ni(phen)(L1)_2_] complex, this band can be mainly attributed to an intraligand electronic transition centered on the phenanthroline fragments. NBO analysis of the orbitals involved indicates that the occupied orbitals are predominantly associated with phenanthroline C–C bonding character, while the virtual orbitals correspond mainly to phenanthroline C–C π* antibonding character (Fig. 10).

Table S13 summarizes the assignments of the remaining bands observed in the experimental spectrum of the [Ni(phen)(L1)_2_] complex. In general, the excited state set of this system is predominantly composed of ligand-centered transitions and charge-transfer transitions involving both the xanthate ligand and phenanthroline. The higher-energy bands are associated with ligand-to-metal charge-transfer processes, with significant participation of the xanthate fragment, indicating the involvement of sulfur donor orbitals in the excitation process. In contrast to the analogous bpy-containing complex, the band around 268 nm does not show a significant contribution from metal-centered orbitals, and it exhibits a predominantly intraligand character involving fragments of the xanthate and phenanthroline ligands. This behavior suggests a lower degree of metal–ligand orbital mixing in this energy region for the phen system, which can be attributed to structural and electronic differences between phenanthroline and bpy. In particular, the more rigid aromatic system of phenanthroline modifies the spatial distribution and symmetry of its π* orbitals, affecting their effective superposition with the nickel orbitals and thus reducing metal–ligand mixing in this excitation region [50]. The band at 303 nm can be correlated with the bands around 310 nm observed for the en and bpy complexes. This occurs because this band corresponds to electronic transitions between fragments of the xanthate ligand (Figure S10). In addition, a band at 463 nm is observed in the experimental spectrum, corresponding to a metal–ligand electronic transition involving the phenanthroline group. This transition is mainly associated with contributions from metal-centered d orbitals and low-energy π* orbitals of phenanthroline (Figure S10). The same behavior is observed for the [Ni(phen)(L2)_2_] complex, with an even higher contribution from phenanthroline-derived orbitals (Table S15). Thus, the band at 225 nm in the experimental spectrum corresponds to an intraligand electronic transition from phenanthroline fragments to the xanthate ligand (Figure S10). Finally, the bands at 309 and 333 nm are assigned to charge-transfer electronic transitions involving the xanthate ligand, with a mixed multiconfigurational character arising from both metal to ligand and ligand-to-metal charge-transfer contributions in this lower energy region. This indicates a higher degree of electronic state mixing in this region, in contrast to the en and bpy containing systems.

### Natural bond orbital analysis

In addition to calculating the natural bond orbitals from the canonical molecular orbitals to identify the orbitals involved in electronic transitions, the second-order stabilization energies (E^2^) of the investigated complexes were also analyzed. This energy interaction corresponds to the stabilization resulting from the orbital overlap. Therefore, it was used to analyze the superposition between the sulfur atoms of the xanthate ligands and the nickel metal center, as well as between the nitrogen atoms of the nitrogen-containing ligands and nickel, in order to evaluate the covalent contribution to metal–ligand (M-L) interactions. In addition, the interaction energies obtained were compared with the energies of homoleptic xanthate complexes, enabling comparison of the degree of stabilization and the nature of interactions in the different systems studied. Table 2 illustrates the second-order energy values for heteroleptic and homoleptic complexes. A comparison between the systems indicates that the presence of the nitrogen ligand causes a significant decrease in the superposition energy between the sulfur atoms of the xanthates and the metal center. This result is expected, since the presence of the bulky ligand causes the sulfur atoms to be more distant from the metal center than in homoleptic complexes, as shown by the bond lengths in Table S2. Therefore, the superposition between the orbitals of the sulfur atoms and nickel is smaller than that observed in the homoleptic complex.

**Table 2:** Second-order perturbation energies from NBO analysis (kcal mol^−1^) for S–Ni and N–Ni donor–acceptor interactions in nickel(II) complexes containing nitrogen-based ligands (en, bpy, and phen) and sulfur-based ligands (L1 and L2)

Complexes	ΣE^2^ S–Ni	ΣE^2^ N–Ni
[Ni(en)(L1)_2_]	67.46	20.10
[Ni(en)(L2)_2_]	67.05	20.21
[Ni(bpy)(L1)_2_]	64.02	21.08
[Ni(bpy)(L2)_2_]	63.61	21.17
[Ni(phen)(L1)_2_]	63.91	20.88
[Ni(phen)(L2)_2_]	63.56	20.97
[Ni(L1)_2_]	276.65	-
[Ni(L2)_2_]	280.17	-

**Fig. 11:**
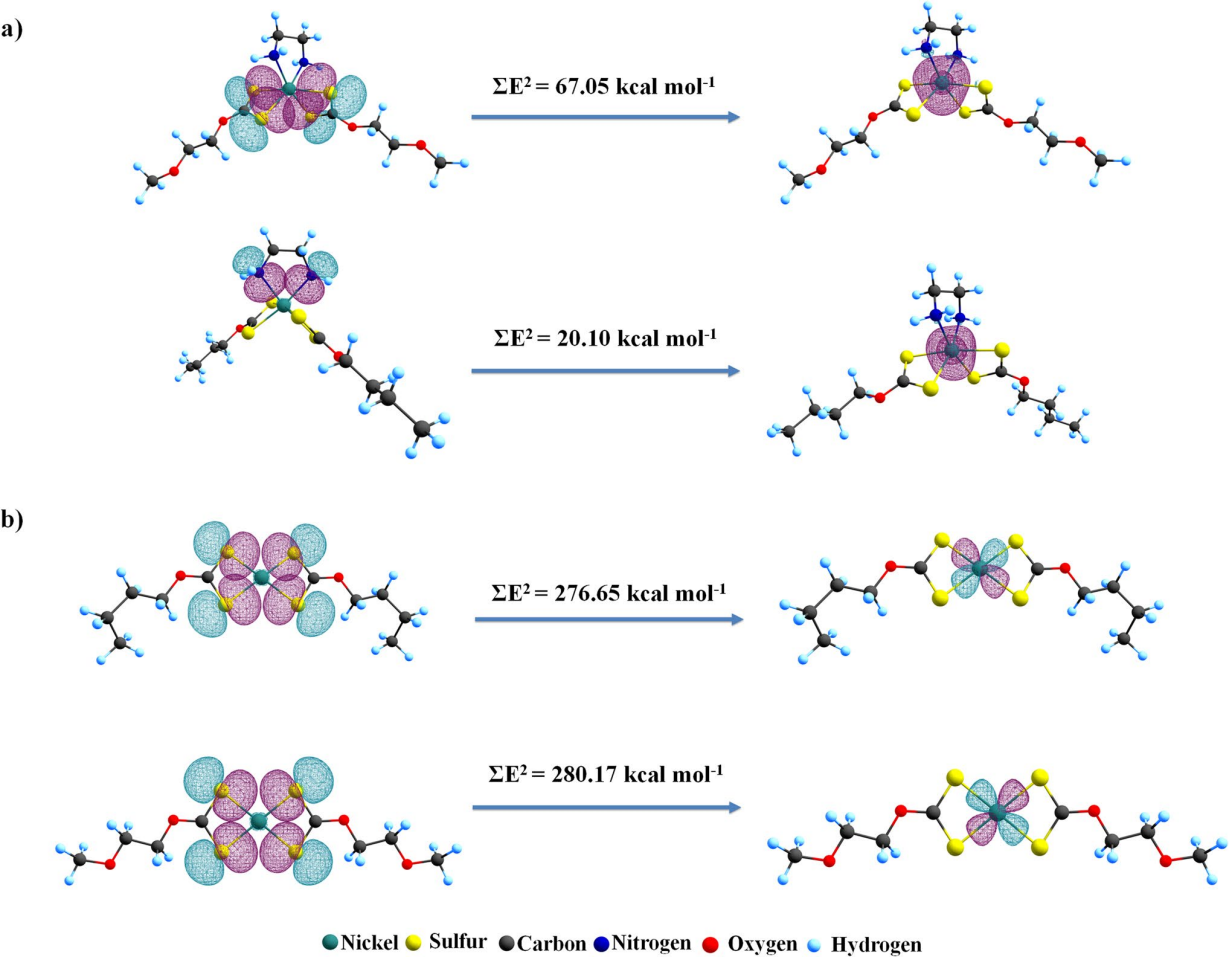
NBO donor–acceptor interactions in nickel complexes for the following: [Ni(en)(L1)_2_] (**a**); [Ni(L1)_2_] and [Ni(L2)_2_] (**b**). Isosurface = 0.04 a.u

The donor–acceptor interaction energy between the nitrogen atoms and the metal center is lower than that observed for the sulfur atoms, suggesting a better affinity of the xanthate ligand with nickel. Furthermore, when comparing the E^2^ N–Ni energies across complexes containing en, bpy, and phen ligands, it can be observed that, although the difference is small, complexes containing bpy and phen exhibit slightly larger E^2^ values. This slightly higher superposition between the orbitals of nitrogen and nickel atoms in aromatic complexes can be explained by the presence of π* orbitals, which allow metal–ligand transitions. However, the high electron density around the metal center, arising from the xanthate and nitrogen-containing ligands, reduces the apparent differences in donor–acceptor stabilization among the N-donor ligands. Furthermore, through NBO analysis it is possible to identify the orbitals involved in the electronic superposition that forms the bonds between the ligands and the metal center. For all heteroleptic complexes, these bonds occur through the p orbitals of the sulfur and nitrogen atoms interacting with the s orbital of the metal center, as illustrated in Fig. 11a for the complex [Ni(en)(L1)_2_]. In the case of the homoleptic complexes, the interaction between the xanthate ligands and the metal center occurs through the p orbitals of the sulfur atoms interacting with the d orbitals of the metal center (Fig. 11b). The natural bond orbitals for all heteroleptic complexes are illustrated in Figures S11–S13.

### Acid–base hardness/softness effects on metal–ligand coordination

From the calculated HOMO and LUMO energies, it is also possible to verify whether the investigated systems conform to Pearson’s concept of acidity and basicity. For this purpose, the values of the hardness index (η) and the softness index (S) were calculated according to Eqs. 12 and 13 [54].

12 $$\eta =\frac{1}{2} \left({\varepsilon}_{LUMO}-{\varepsilon}_{HOMO}\right)$$

13 $$S=\frac{1}{\eta }$$

Table 3 illustrates the values of the hardness and softness indices, as well as the HOMO and LUMO energies, for the heteroleptic and homoleptic complexes. A comparison between the heteroleptic complexes containing the en ligand and the homoleptic complexes shows that the heteroleptic complexes exhibit a higher hardness index (η). This result agrees with what is expected, since the addition of a harder species results in more localized/rigid interactions. On the other hand, an analysis of the complexes containing aromatic nitrogen ligands shows that these systems present a lower hardness index than both the complexes with the en ligand and the homoleptic complexes. This result corroborates what was observed in the electronic analysis, in which the effect of the presence of low-energy π* orbitals in the aromatic compounds was discussed (the “UV − Vis electronic analysis” section). Through electronic analysis using NBO, it is possible to verify that the HOMO and LUMO of the complexes with aromatic ligands correspond to orbitals centered on the sulfur atoms of the xanthates and on the C–C and C–N bonds of the nitrogen ligands (Tables S10–S15). The presence of a low-energy LUMO in these aromatic complexes leads to a smaller HOMO–LUMO gap and, consequently, to a lower hardness index. In the case of the complexes with the en ligand, the Hβ and Lβ orbitals correspond to orbitals centered on the xanthate ligands and on the metal center, respectively (Tables S7–S9). As a result, the trend predicted by Pearson’s theory is observed only for the heteroleptic complexes with the en ligand and for the homoleptic complexes. The other heteroleptic complexes do not follow Pearson’s trend due to their electronic nature.

**Table 3:** Frontier orbital energies (E_HOMO_, E_LUMO_), hardness index (η), and softness index (S) of the Ni(II) complexes

Complexes	E_LUMO_ (kcal mol^−1^)	E_HOMO_ (kcal mol^−1^)	η	S
[Ni(en)(L1)_2_]	−31.60	−94.80	31.60	0.03
[Ni(en)(L2)_2_]	−33.85	−96.79	31.47	0.03
[Ni(bpy)(L1)_2_]	−66.97	−90.89	11.96	0.08
[Ni(bpy)(L2)_2_]	−68.05	−92.87	12.41	0.08
[Ni(phen)(L1)_2_]	−66.21	−90.71	12.25	0.08
[Ni(phen)(L2)_2_]	−67.25	−92.70	12.73	0.08
[Ni(L1)_2_]	−68.21	−108.87	20.33	0.05
[Ni(L2)_2_]	−69.65	−109.94	20.14	0.05

## Conclusions

In this work, a theoretical study supported by experimental spectroscopic characterization of six heteroleptic nickel(II) complexes containing xanthate ligands (*n*-butyl and 2-methoxyethyl) and aliphatic and aromatic nitrogen-donor ligands (en, bpy, and phen) was carried out. The study focused on understanding the electronic nature of the metal–ligand interactions through thermochemistry, UV–Vis spectroscopy, and NBO analysis. Thermochemical data indicate that the formation of all heteroleptic complexes is predicted to be thermodynamically favorable and exothermic processes, regardless of the order in which the ligands are added to the nickel acetate solution. Although the formation of intermediates in both sequences is endothermic and thermodynamically unfavorable (which is consistent with the absence of experimental precipitation), the final coordination step compensates for this energetic cost. The comparative analysis indicates that both addition sequences lead to thermodynamically comparable complexes. Although small energetic fluctuations are observed, with a slight preference for sequence 1 in systems containing ethylenediamine, the overall differences are minor and do not establish a dominant trend, suggesting that the synthesis can support the feasibility of both sequences. In the comparison between ligands L1 and L2, steric and electronic factors were found to exert minor influences, primarily governed by enthalpic effects. Complexes containing ethylenediamine (en) exhibited slightly more favorable free energies due to reduced steric hindrance. On the other hand, this effect is partially counterbalanced in aromatic ligands (bpy and phen) due to their π-acceptor character, which favors interactions from the metal center to the π* orbitals. In the case of xanthates, the substitution of L1 by L2 resulted in variations of only approximately 1 kcal mol^−1^ in thermodynamic stability (ΔG). This behavior indicates that the electron-withdrawing inductive effect of the heteroatom oxygen in the 2-methoxyethyl xanthate ligand (L2) is moderate. In addition to the thermochemical analysis, the electronic UV–Vis spectra obtained experimentally were assigned through TD-DFT analysis and natural bond orbitals. The observed bands were attributed to intraligand, metal–ligand, and ligand–metal electronic transitions, with an increasing trend of multiconfigurational character in the order en < bpy < phen. In addition, a greater contribution from metal–ligand charge-transfer transitions was observed in the bpy and phen complexes, due to the presence of low-energy π* orbitals. However, for the complexes containing ethylenediamine, differences between ligands L1 and L2 were observed, since the system is non-aromatic and does not exhibit contributions from low-energy π* orbitals. Thus, the effect of the oxygen heteroatom in the structure of ligand L2 becomes more pronounced in the ethylenediamine complex. From these systems, it was observed that the 2-methoxyethyl xanthate ligand exhibits predominantly MLCT character, whereas the complex with ligand L1 shows a higher LMCT contribution. This behavior can be attributed to the electron-withdrawing nature of the oxygen atom, which reduces the electron density at the coordinating sulfur site, consequently decreasing ligand-to-metal donation and increasing the relative importance of metal-to-ligand charge-transfer contributions. Finally, the NBO analysis showed that, in the heteroleptic complexes, the interactions between the metal and the ligands occur through orbital overlap between the p orbitals of the sulfur/nitrogen and the s orbital of the metal center. In addition, the hardness and softness analysis based on Pearson’s theory was found to be valid for the homoleptic complexes when compared to the heteroleptic complexes containing the en ligand. However, the heteroleptic complexes containing aromatic nitrogen-donor ligands deviate from the trend expected from this theory. This behavior was justified by the presence of low-energy π* orbitals, which stabilize the LUMO and reduce the HOMO–LUMO gap. As a result, the applicability of the Pearson model in these systems is limited, reinforcing the importance of electronic effects in these complexes, rather than relying exclusively on hardness and softness arguments.

## Supplementary Information

Below is the link to the electronic supplementary material.

ESM 1 (DOCX 4.26 MB)

## Data Availability

All the data generated or analyzed during this study are included in this published article and its supplementary information file.

## References

[1] Polak AM, Karska K, Drozd M (2025) The discovery of medicines: drug testing on humans and the development of medical ethics before the 20th century. Drug Discov Today 30:104418. 10.1016/j.drudis.2025.104418 40571053 10.1016/j.drudis.2025.104418

[2] Fleming A (1929) On the antibacterial action of cultures of a penicillium, with special reference to their use in the isolation of B. influenza. Br J Exp Pathol 10:226–236 PMC2566493 11545337

[3] Wali AF, Talath S, Sridhar SB, Shareef J, Goud M, Rangraze IR, Alaani NN, Mohamed OI (2024) A comprehensive review on bioactive molecules and advanced microorganism management technologies. Curr Issues Mol Biol 46:13223.10.3390/cimb46110789 10.3390/cimb46110789 PMC11592628 39590383

[4] Jadimurthy R, Jagadish S, Nayak SC, Kumar S, Mohan CD, Rangappa KS (2023) Phytochemicals as invaluable sources of potent antimicrobial agents to combat antibiotic resistance. Life 13:948. 10.3390/life13040948 37109477 10.3390/life13040948 PMC10145550

[5] Andreani T, Cheng R, Elbadri K, Ferro C, Menezes T, dos Santos MR, Pereira CM, Santos HA (2024) Natural compounds-based nanomedicines for cancer treatment: future directions and challenges. Drug Deliv Transl Res 14:2845–2916. 10.1007/s13346-024-01649-z 39003425 10.1007/s13346-024-01649-z PMC11385056

[6] Matthews HK, Bertoli C, de Bruin RAM (2022) Cell cycle control in cancer. Nat Rev Mol Cell Biol 23:74-88. 10.1038/s41580-021-00404-3 10.1038/s41580-021-00404-3 34508254

[7] Zafar A, Khatoon S, Khan MJ, Abu J, Naeem A (2025) Advancements and limitations in traditional anti-cancer therapies: a comprehensive review of surgery, chemotherapy, radiation therapy, and hormonal therapy. Discov Oncol 16:607. 10.1007/s12672-025-02198-8 10.1007/s12672-025-02198-8 PMC12021777 40272602

[8] Mensforth EJ, Hill MR, Batten SR (2013) Coordination polymers of sulphur-donor ligands. Inorg Chim Acta 403:9-24. 10.1016/j.ica.2013.02.019

[9] Lv P, Yang Y, Li N, Zhang Y, Hu M, Huang B, Zhu Y, Wang Y, Pan J, Wang S, Zhang B, Huang F, Cheng YB, Lu J (2023) Hypervalent potassium xanthate modified SnO_2_ for highly efficient perovskite solar modules. Chem Eng J 456:140894. 10.1016/j.cej.2022.140894

[10] Wu W-J, Zheng QJ, Liang JW, Zhao HM, Liu BL, Li YW, Feng NX, Cai QY, Xiang L, Mo CH, Li QX (2024) Mining flotation reagents: quantitative and robust analysis of metal-xanthate complexes in water. J Hazard Mater 476:134873. 10.1016/j.jhazmat.2024.134873 38908182 10.1016/j.jhazmat.2024.134873

[11] Eensalu JS, Mandati S, Don CH, Finch H, Dhanak VR, Major JD, Grzibovskis R, Tamm A, Ritslaid P, Josepson R, Käämbre T, Vembris A, Spalatu N, Krunks M, Oja Acik I (2023) Sb2S3 thin-film solar cells fabricated from an antimony ethyl xanthate based precursor in air. ACS Appl Mater Interfaces 15:42622–42636. 10.1021/acsami.3c08547 37640298 10.1021/acsami.3c08547 PMC10510044

[12] Bakbardina OV, Pukhnyarskaya IYu, Gazalieva MA, Fazylov SD, Makarov EM (2006) Synthesis and fungicidal activity of S-amino derivatives of O-alkyldithiocarbonic acids. Russ J Appl Chem 79:1726–1728. 10.1134/S1070427206100375

[13] Torshizi MH, Zareian Jahromi S, Saeidifar M, Ghasemi A, Ghaemi H, Heydari A (2017) Synthesis, characterization and in vitro antimicrobial screening of the xanthate derivatives and their iron(II) complexes. Iran J Chem Chem Eng 36:43–48. 10.30492/ijcce.2017.25477

[14] Ragshaniya A, Asija S, Lal K, Deswal Y, Gupta NM, Barwa P, Poonia S (2025) Exploring the antimicrobial, antioxidant efficacies and computational studies of tridentate ligands derived from xanthene Schiff base and their mononuclear diorganotin(IV) complexes. Inorg Chem Commun 178:114543. 10.1016/j.inoche.2025.114543

[15] Drbas SA, Molla-Babaker MM, Hami MA (2026) Synthesis, characterization, and biological evaluation of novel xanthate ligands and their divalent metal complexes: DFT calculations and molecular docking studies Inorg Chim Acta 589:122894. 10.1016/j.ica.2025.122894

[16] Friebolin W, Schilling G, Zöller M, Amtmann E (2005) Antitumoral activity of non-platinum xanthate complexes. J Med Chem 48:7925–7931. 10.1021/jm040899l 16335917 10.1021/jm040899l

[17] Shen Z, Ma N, Hou C, Chen X, Chao S, Pei Y, Pei Z (2022) Tumor microenvironment dual-responsive nanovesicles from one functional group based on a water-soluble xanthate capped pillar[5]arene for enhancing the effect of chemotherapy. Colloids Surf A Physicochem Eng Asp 648:129262. 10.1016/j.colsurfa.2022.129262

[18] Singh VK, Kadu R, Roy H, Raghavaiah P, Mobin SM (2016) Phenolate based metallomacrocyclic xanthate complexes of CoII/CuII and their exclusive deployment in [2:2] binuclear N,O-Schiff base macrocycle formation and in vitro anticancer studies. Dalton Trans 45:1443-1454. 10.1039/c5dt03407h 10.1039/c5dt03407h 26674056

[19] Alam N, Ehsan MA, Zeller M, Mazhar M, Arifin Z (2011) Bis(O-n-butyl dithiocarbonato-κ^2^S,S’)-bis(pyridine-κN)manganese(II) Acta Crystallogr Sect E Struct Rep EE67:m1064. 10.1107/S1600536811026523 10.1107/S1600536811026523 PMC3212145 22090847

[20] Zhang W, Jiang X, Zhong Y, Tan M, Wang S (2002) Coordination cadmium compounds with 1,10-phenanthroline. Collect Czech Chem Commun 67:1623-1630. 10.1135/cccc20021623

[21] Kour G, Kumar A, Kour I, Kour G, Sachar R, Gupta VK, Rajnikant (2012) Crystal structure of bis(O-n-butyldithiocarbonato-κ^2^S,S′)(1,10-phenanthroline)manganese(II). X-ray Struct Anal Online 28:85–86. 10.2116/xraystruct.28.85

[22] Sharma N, Singh K, Sachar R, Gupta VK, Rajnikant (2013) Crystal structure of bis(O-propyldithiocarbonato-κ^2^S,S′)(3,5-lutidine-κN)nickel(II). X-ray Struct Anal Online 29:19–20. 10.2116/xraystruct.29.19

[23] Singh K, Neerupama, Kour I, Sachar R, Gupta VK, Rajnikant V (2012) Synthesis and characterization of the adducts of bis(O-amyldithiocarbonato)nickel(II) with nitrogen donors and X-ray structure of bis(O-amyldithiocarbonato)bis(3,5-dimethylpyridine)nickel(II). J Chem Crystallogr 42:1176-1181. 10.1007/s10870-012-0373-y

[24] Howlader P, Schmittel M (2022) Heteroleptic metallosupramolecular aggregates/complexation for supramolecular catalysis. Beilstein J Org Chem 18:597-630. 10.3762/bjoc.18.62 10.3762/bjoc.18.62 PMC9152274 35673407

[25] Mandal T, Ghosh M, Paps H, Mandal T, Reiser O (2025) A general photocatalytic platform for the regio- and stereoselective β-chloroacylation of alkenes and alkynes using a heteroleptic copper(I) complex. Nat Catal 8:607-622. 10.1038/s41929-025-01357-y

[26] Prusty S, Chan Y-T (2021) Terpyridine-based self-assembled heteroleptic coordination complexes. Chem Lett 50:1202–1212. 10.1246/cl.210048

[27] Jagadesh Babu K, Daravath S, Swathi M, Ayodhya D, Shivaraj (2023) Synthesis, anticancer, antibacterial, antifungal, DNA interactions, ADMET, molecular docking, and antioxidant evaluation of novel Schiff base and their Co(II), Ni(II) and Cu(II) complexes. Results Chem 6:101121. 10.1016/j.rechem.2023.101121

[28] Prashanthi Y, Kiranmai K, Ira, Kumar KS, Chityala VK, Shivaraj (2012) Spectroscopic characterization and biological activity of mixed ligand complexes of Ni(II) with 1,10-phenanthroline and heterocyclic Schiff bases. Bioinorg Chem Appl 2012:948534. 10.1155/2012/948534 10.1155/2012/948534 PMC3467759 23082074

[29] Mansour AM (2021) Pd(II) and Pt(II) complexes of tridentate ligands with selective toxicity against *Cryptococcus neoformans* and *Candida albicans*. RSC Adv 11:39748-39756. 10.1039/d1ra06559a 10.1039/d1ra06559a PMC9044551 35494132

[30] Qadir AM (2017) Synthesis, characterization and antibacterial activities of two nickel(II) complexes with xanthate derivatives and N,N,N’,N’-tetramethylethylenediamine as ligands. Transit Metal Chem 42:35–39. 10.1007/s11243-016-0103-y

[31] de Miranda DB, Quintal S, Ferreira GB (2024) Electronic analysis of n-propyl xanthate complexes with group 12 metals: a theoretical-experimental study. J Mol Model 30:163. 10.1007/s00894-024-05950-z 10.1007/s00894-024-05950-z 38730058

[32] Molla-Babaker MM, Khalid M, AL-Mukhtar SE (2024) Synthesis, characterization and DFT study of novel xanthate ligand complexes with manganese (II), iron (II), cobalt (II), nickel (II), copper (II), and zinc (II) and their adducts with nitrogen base ligands. AIP Conf Proc 2944:020018. 10.1063/5.0205255

[33] Wang Y, Jin X, Yu HS, Truhlar DG, He X (2017) Revised M06-L functional for improved accuracy on chemical reaction barrier heights, noncovalent interactions, and solid-state physics. Proc Natl Acad Sci USA 114:8487-8492. 10.1073/pnas.1705670114 10.1073/pnas.1705670114 PMC5559035 28739954

[34] Weigend F, Ahlrichs R (2005) Balanced basis sets of split valence, triple zeta valence and quadruple zeta valence quality for H to Rn: design and assessment of accuracy. Phys Chem Chem Phys 7:3297-3305. 10.1039/b508541a 10.1039/b508541a 16240044

[35] Duarte HA (2025) Fundamentos da teoria do funcional de densidade (DFT): uma revisão histórica e conceitual. Rev Virtual Quim, no prelo:1–12. 10.21577/1984-6835.20250068

[36] Zhao Y, Truhlar DG (2008) Density functionals with broad applicability in chemistry. Acc Chem Res 41:157–167. 10.1021/ar700111a 18186612 10.1021/ar700111a

[37] Tomasi J, Mennucci B, Cammi R (2005) Quantum mechanical continuum solvation models. Chem Rev 105:2999-3093. 10.1021/cr9904009 10.1021/cr9904009 16092826

[38] Casida ME, Huix-Rotllant M (2012) Progress in time-dependent density-functional theory. Annu Rev Phys Chem 63:287-323. 10.1146/annurev-physchem-032511-143803 10.1146/annurev-physchem-032511-143803 22242728

[39] Frisch MJ, Trucks GW, Schlegel HB, Scuseria GE, Robb MA, Cheeseman JR, Scalmani G, Barone V, Petersson GA, Nakatsuji H, Li X, Caricato M, Marenich AV, Bloino J, Janesko BG, Gomperts R, Mennucci B, Hratchian HP, Ortiz JV, Izmaylov AF, Sonnenberg JL, Williams-Young D, Ding F, Lipparini F, Egidi F, Goings J, Peng B, Petrone A, Henderson T, Ranasinghe D, Zakrzewski VG, Gao J, Rega N, Zheng G, Liang W, Hada M, Ehara M, Toyota K, Fukuda R, Hasegawa J, Ishida M, Nakajima T, Honda Y, Kitao O, Nakai H, Vreven T, Throssell K, Montgomery JA Jr, Peralta JE, Ogliaro F, Bearpark MJ, Heyd JJ, Brothers E, Kudin KN, Staroverov VN, Keith T, Kobayashi R, Normand J, Raghavachari K, Rendell A, Burant JC, Iyengar SS, Tomasi J, Cossi M, Millam JM, Klene M, Adamo C, Cammi R, Ochterski JW, Martin RL, Morokuma K, Farkas O, Foresman JB, Fox DJ (2013) Gaussian 09, Revision D.01. Gaussian, Inc., Wallingford CT

[40] O’Boyle NM, Tenderholt AL, Langner KM (2008) Cclib: a library for package-independent computational chemistry algorithms. J Comp Chem 29:839-845. 10.1002/jcc.20823 17849392

[41] Kočanová I, Kuchár J, Orendáč M, Černák J (2010) Cu-Ni heterobimetallic compounds. Part 2: study of the system Cu(II)-bpy-[Ni(CN)4](2−) (bpy = 2,2′-bipyridine). Polyhedron 29:3372–3379. 10.1016/j.poly.2010.09.018

[42] Alramadhan SA, Hammud HH, Ali BF, Ghabbour HA, Sarfaraz S, Ayub K (2023) Structures, characterization and DFT studies of four novel nickel phenanthroline complexes. Crystals 13:738. 10.3390/cryst13050738

[43] Franzini M (1963) The crystal structure of nickelous xanthate. Zeitschrift für Kristallographie 118: 393-403. 10.1524/zkri.1963.118.16.393

[44] Little LH, Poling GW, Leja J (1961) Infrared spectra of xanthate compounds: II. Assignment of vibrational frequencies. Can J Chem 39:745-754. 10.1139/v61-090

[45] Berg RW, Rasmussen K (1974) Infrared and far infrared spectra of bis(ethylenediamine)nickel(II)-tri- and tetra-iodomercurate(II) Spectrochim. Acta A Mol Spectrosc 30:1881-1887. 10.1016/0584-8539(74)80139-X

[46] Gerasimova TP, Katsyuba SA (2013) Bipyridine and phenanthroline IR-spectral bands as indicators of metal spin state in hexacoordinated complexes of Fe(II), Ni(II) and Co(II). Dalton Trans 42:1787–1797. 10.1039/C2DT31922E 10.1039/c2dt31922e 23165737

[47] Yang LF, Peng ZH, Wang SX, Fang CJ (2004) Spectroscopic and theoretical studies on nickel(II) complex of maleonitriledithiolate and 2,2′-bipyridine. Spectrochim Acta A Mol Biomol Spectrosc 60:481-487. 10.1016/S1386-1425(03)00253-1 10.1016/s1386-1425(03)00253-1 14670516

[48] Trávníček Z, Walla J, Kvítek L, Šindelář Z, Biler M (1999) Xanthate complexes of nickel with nitrogen donor ligands Part V Transition Met Chem 24:633-637. 10.1023/A:1006923722773

[49] Downie TC, Harrison W, Raper ES, Hepworth MA (1971) A three-dimensional study of the crystal structure of nickel acetate tetrahydrate. Acta Crystallogr B27:706-712. 10.1107/S0567740871002802

[50] Bencini A, Lippolis V (2010) 1,10-Phenanthroline: a versatile building block for the construction of ligands for various purposes Coord Chem Rev 254:2096-2180. 10.1016/j.ccr.2010.04.008

[51] Nelson KJ, Kazmierczak NP, Cagan DA, Follmer AH, Scott TR, Raj SL, Garratt D, Powers-Riggs N, Gaffney KJ, Hadt RG, Cordones AA (2025) Multiconfigurational electronic structure of nickel cross-coupling catalysts revealed by X-ray absorption spectroscopy. J Phys Chem Lett 16:87-94. 10.1021/acs.jpclett.4c02917 10.1021/acs.jpclett.4c02917 PMC11726796 39700059

[52] Cagan DA, Bím D, Kazmierczak NP, Hadt RG (2024) Mechanisms of photoredox catalysis featuring nickel-bipyridine complexes. ACS Catal 14:9055–9076. 10.1021/acscatal.4c02036 38868098 10.1021/acscatal.4c02036 PMC11165457

[53] Batista VHSC, Granato AC, Angelotti WFD (2016) Comparação entre funcionais de densidade no estudo de propriedades eletrônicas de derivados da artemisinina. Quim Nova 39(3). 10.5935/0100-4042.20160040

[54] Pearson RG (1968) Hard and soft acids and bases, HSAB, part I: fundamental principles. J Chem Educ 45:581–587. 10.1021/ed045p581

