# Supplementary material for: Electronic effects on the stability of heteroleptic nickel(II) complexes with aromatic and aliphatic ligands
Source: J Mol Model. 2026 Jul 8;32(8):265. doi: 10.1007/s00894-026-06833-1 (PMC13346210; doi:10.1007/s00894-026-06833-1)
Supplement: Supplementary file 1 — (DOCX 4.26 MB) [file 894_2026_6833_MOESM1_ESM.docx]

**Supplementary Information**

**Electronic effects on the stability of heteroleptic nickel(II) complexes with aromatic and aliphatic ligands**

Daniella B. de Miranda,^a^ Thalita R. André^a^, Glaucio B. Ferreira^a*^

^a^Instituto de Química, Universidade Federal Fluminense, Outeiro de S. João Batista s/n., Centro, 24210-130, Niterói, RJ, Brazil.

*glauciobf@id.uff.br

**Keywords:** Xanthate; Nickel; Heteroleptic; M06L; NBO; DFT.

**Table of Contents**

1. Syntheses **3**
2. Optimized geometry structures **8**
3. Thermochemical Analysis **10**
4. UV-Vis electronic analysis **12**
5. Natural bond orbital analysis **34**
6. **Syntheses**

***n*-butyl xanthate (L1)**: IR (ATR) ν / cm^-1^ 1263, 1227, 1176, 1147, 1104, 1057, 995, 958, 923, 835, 744, 671; Raman ν / cm^-1^ 1273, 1238, 1154, 1132, 1108, 1067, 1028, 884, 844, 676. Yield = 46%

**2-methoxyethyl xanthate (L2)**: IR (ATR) ν / cm^-1^ 1273, 1231, 1195, 1161, 1135, 1122, 1100, 1066, 1017, 975, 887, 845, 752, 676; Raman ν / cm^-1^ 1260, 1224, 1178, 1147, 1132, 1110, 1080, 1062, 1056, 1016, 961, 924, 840, 748, 672. Yield = 48%


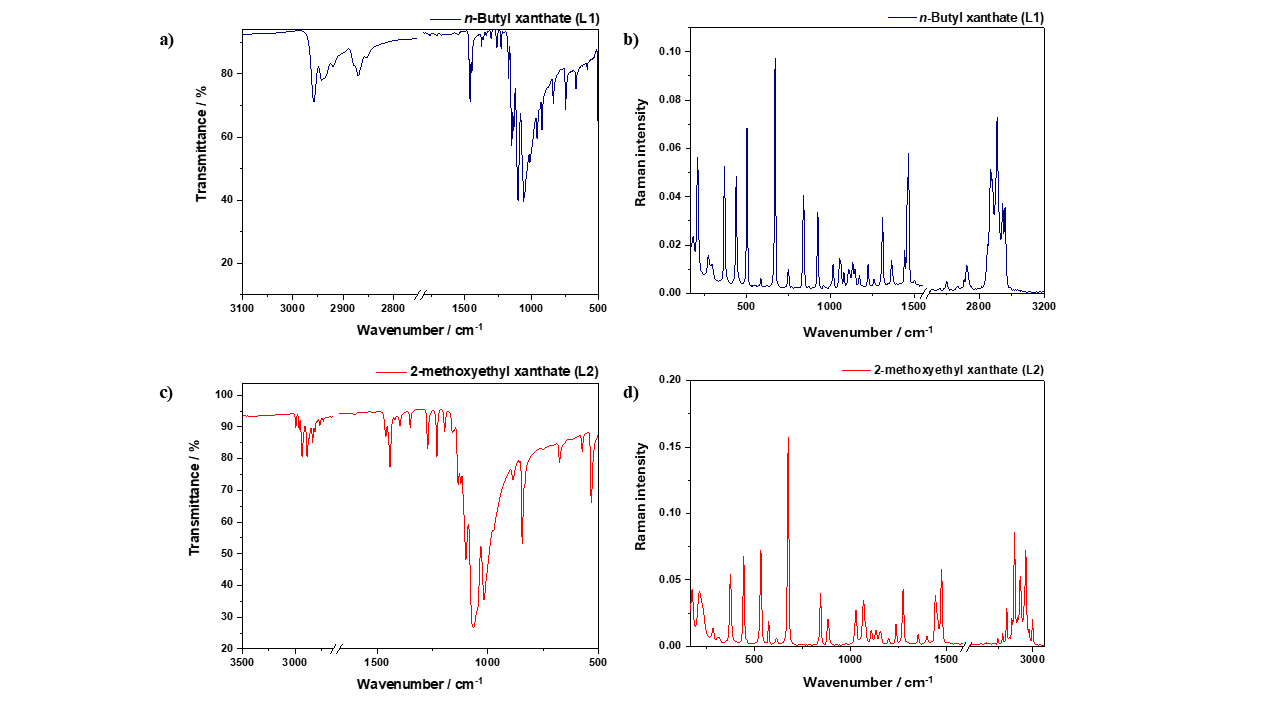


**Figure S1.** Infrared and Raman spectra of: **a** and **b)** n-butyl xanthate (L1); **c** and **d)** 2-methoxyethyl xanthate (L2).


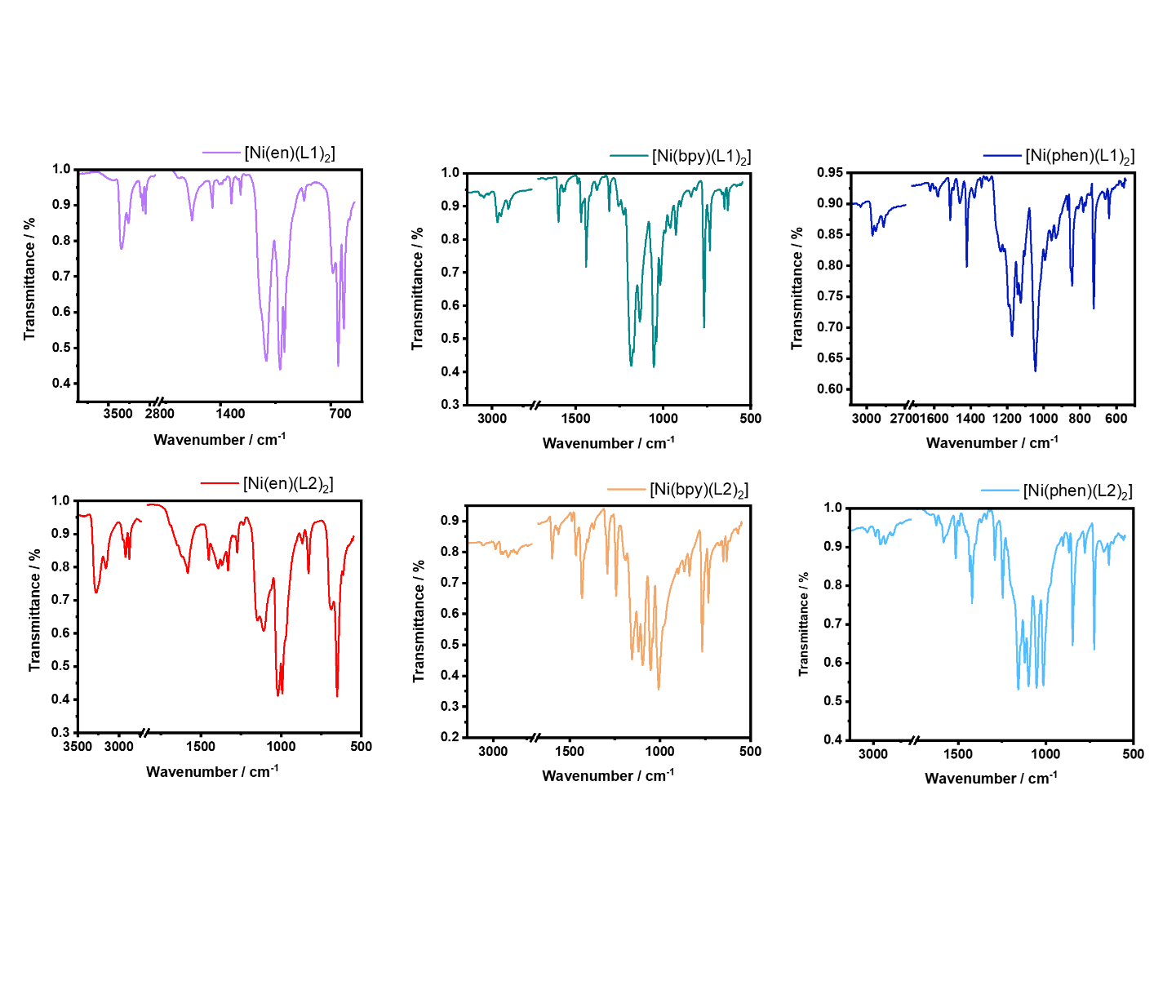


**Figure S2.** Experimental Infrared spectra of heteroleptic complexes.


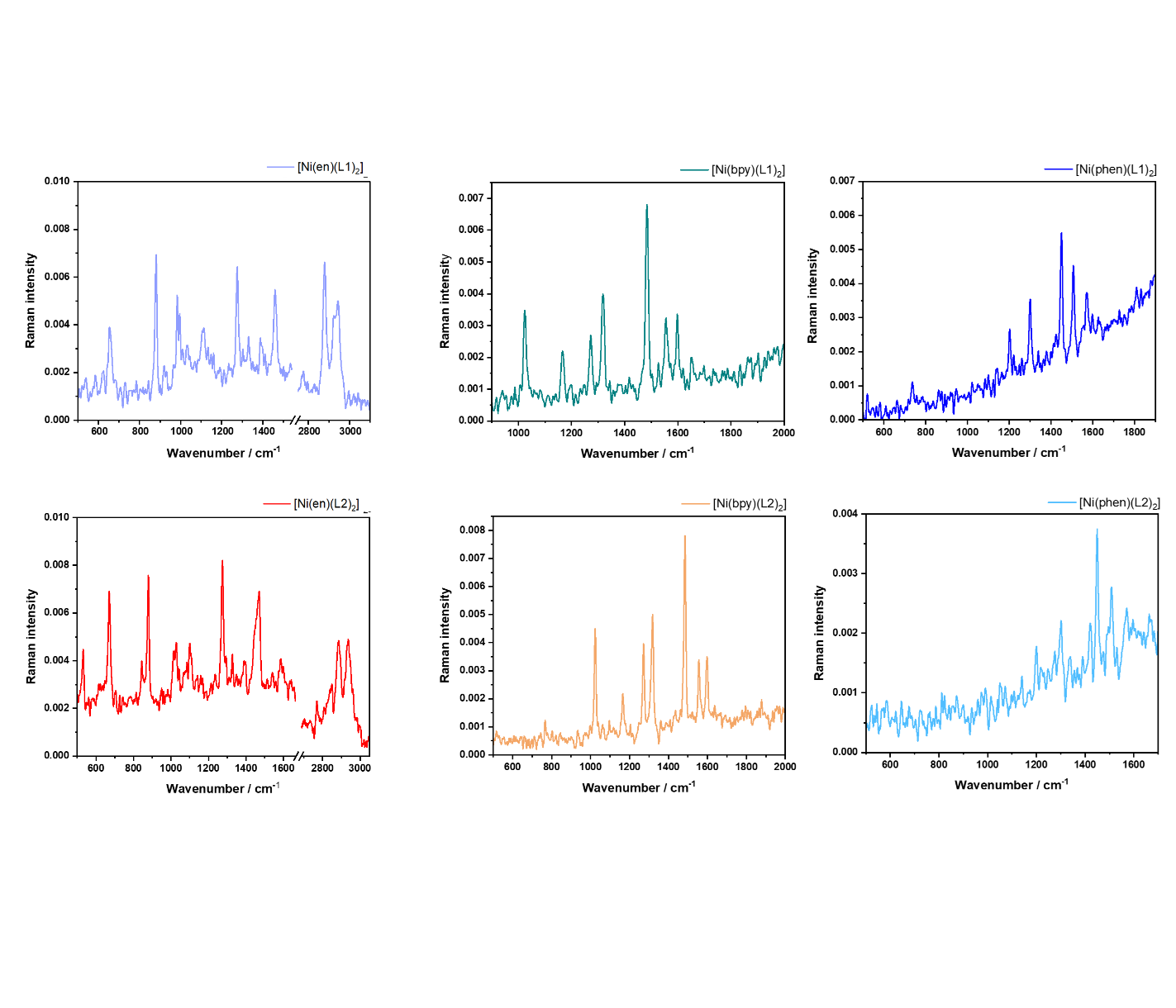


**Figure S3.** Experimental Raman spectra of heteroleptic complexes.


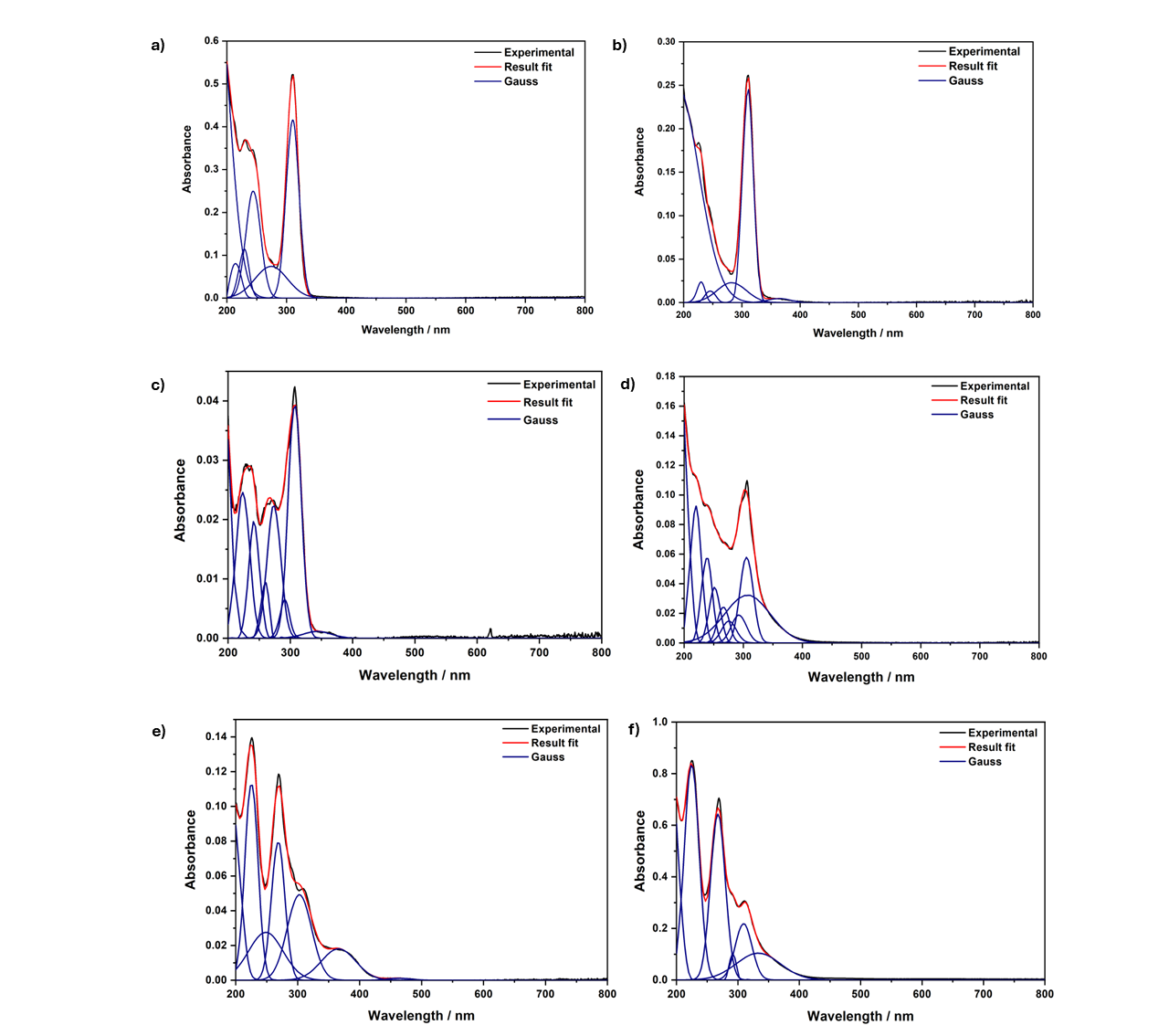


**Figure S4.** Experimental UV-Vis spectra of heteroleptic complexes (10^-5^ M): **a)** [Ni(en)(L1)_2_]; **b)** [Ni(en)(L2)_2_]; **c)** [Ni(bpy)(L1)_2_]; **d)** [Ni(bpy)(L2)_2_]; **e)** [Ni(phen)(L1)_2_]; **f)** [Ni(phen)(L2)_2_].


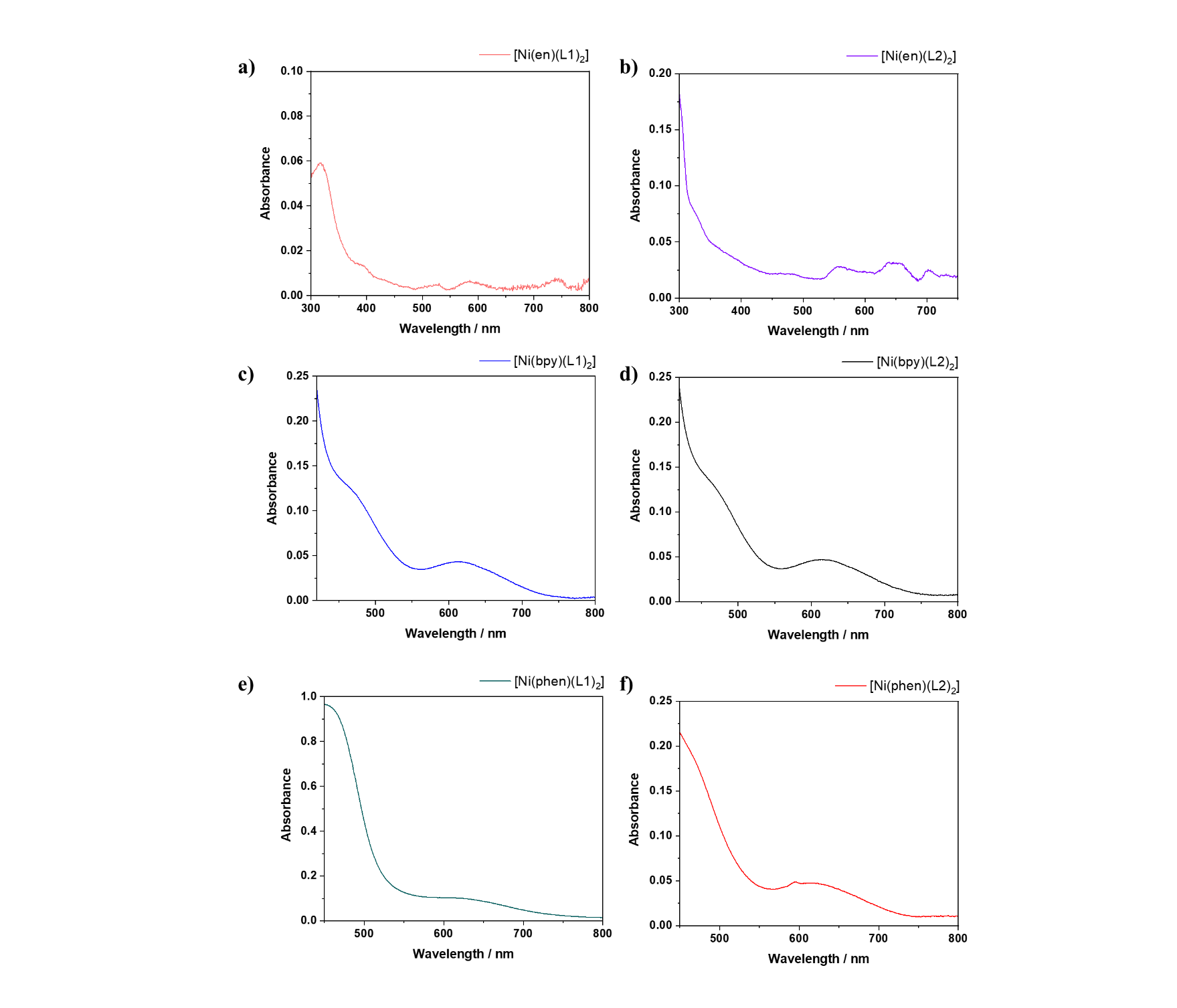


**Figure S5.** Experimental UV-Vis spectra of heteroleptic complexes (10^-3^ M): **a)** [Ni(en)(L1)_2_]; **b)** [Ni(en)(L2)_2_]; **c)** [Ni(bpy)(L1)_2_]; **d)** [Ni(bpy)(L2)_2_]; **e)** [Ni(phen)(L1)_2_]; **f)** [Ni(phen)(L2)_2_].

**Table S1.** Wavelength-dependent UV-Vis absorption data, absorbance values, and molar absorptivities (ε) for the investigated nickel(II) heteroleptic complexes in acetonitrile solution (10^-5^ M).

| **Wavelength (nm)** | **Absorbance** | **Molar absorptivity  (L mol^-1^ cm^-1^)** |
| --- | --- | --- |
| **[Ni(en)(L1)_2_]** | | |
| 180 | 0.79527 | 79527 |
| 215 | 0.11825 | 11825 |
| 228 | 0.15966 | 15966 |
| 244 | 0.28489 | 28489 |
| 274 | 0.07599 | 7599 |
| 310 | 0.49371 | 49371 |
| **[Ni(bpy)(L1)_2_]** | | |
| 185 | 0.06555 | 6555 |
| 224 | 0.02479 | 2479 |
| 242 | 0.0198 | 1980 |
| 258 | 0.01025 | 1025 |
| 273 | 0.02261 | 2261 |
| 290 | 0.00893 | 893 |
| 307 | 0.04001 | 4001 |
| 343 | 0.00115 | 115 |
| **[Ni(phen)(L1)_2_]** | | |
| 196 | 0.09368 | 9368 |
| 225 | 0.11513 | 11513 |
| 268 | 0.02754 | 2754 |
| 248 | 0.08028 | 8028 |
| 303 | 0.04918 | 4918 |
| 366 | 0.01804 | 1804 |
| 463 | 0.00118 | 118 |
| **[Ni(en)(L2)_2_]** | | |
| 189 | 0.2492 | 24920 |
| 229 | 0.03675 | 3675 |
| 246 | 0.01943 | 1943 |
| 282 | 0.02299 | 2299 |
| 310 | 0.24802 | 24802 |
| 364 | 0.00462 | 462 |
| **[Ni(bpy)(L2)_2_]** | | |
| 197 | 0.07889 | 7889 |
| 219 | 0.0925 | 9250 |
| 238 | 0.05871 | 5871 |
| 251 | 0.03766 | 3766 |
| 266 | 0.02489 | 2489 |
| 275 | 0.01484 | 1484 |
| 292 | 0.0189 | 1890 |
| 305 | 0.02927 | 2927 |
| 307 | 0.0324 | 3240 |
| **[Ni(phen)(L2)_2_]** | | |
| 195 | 0.33753 | 33753 |
| 225 | 0.42016 | 42016 |
| 267 | 0.64794 | 64794 |
| 291 | 0.10241 | 10241 |
| 309 | 0.21902 | 21902 |
| 333 | 0.10389 | 10389 |

1. **Optimized geometry structures**

**
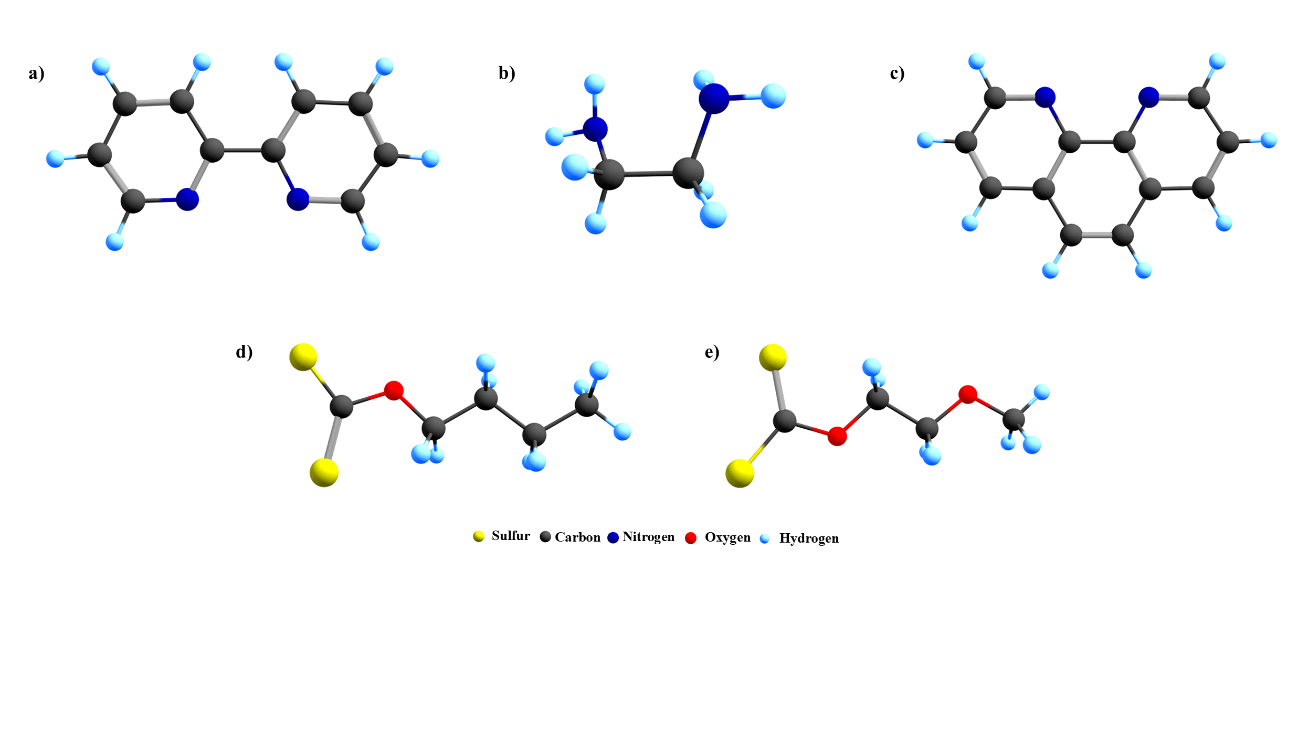
**

**Figure S6.** Optimized structures of ligands (CPCM = ethanol): **a)** 2,2’-bipyridine (bpy); **b)** ethylenediamine (en); **c)** 1,10-phenanthroline (phen); **d)** n-butyl xanthate; **e)** 2-methoxyethyl xanthate.

**
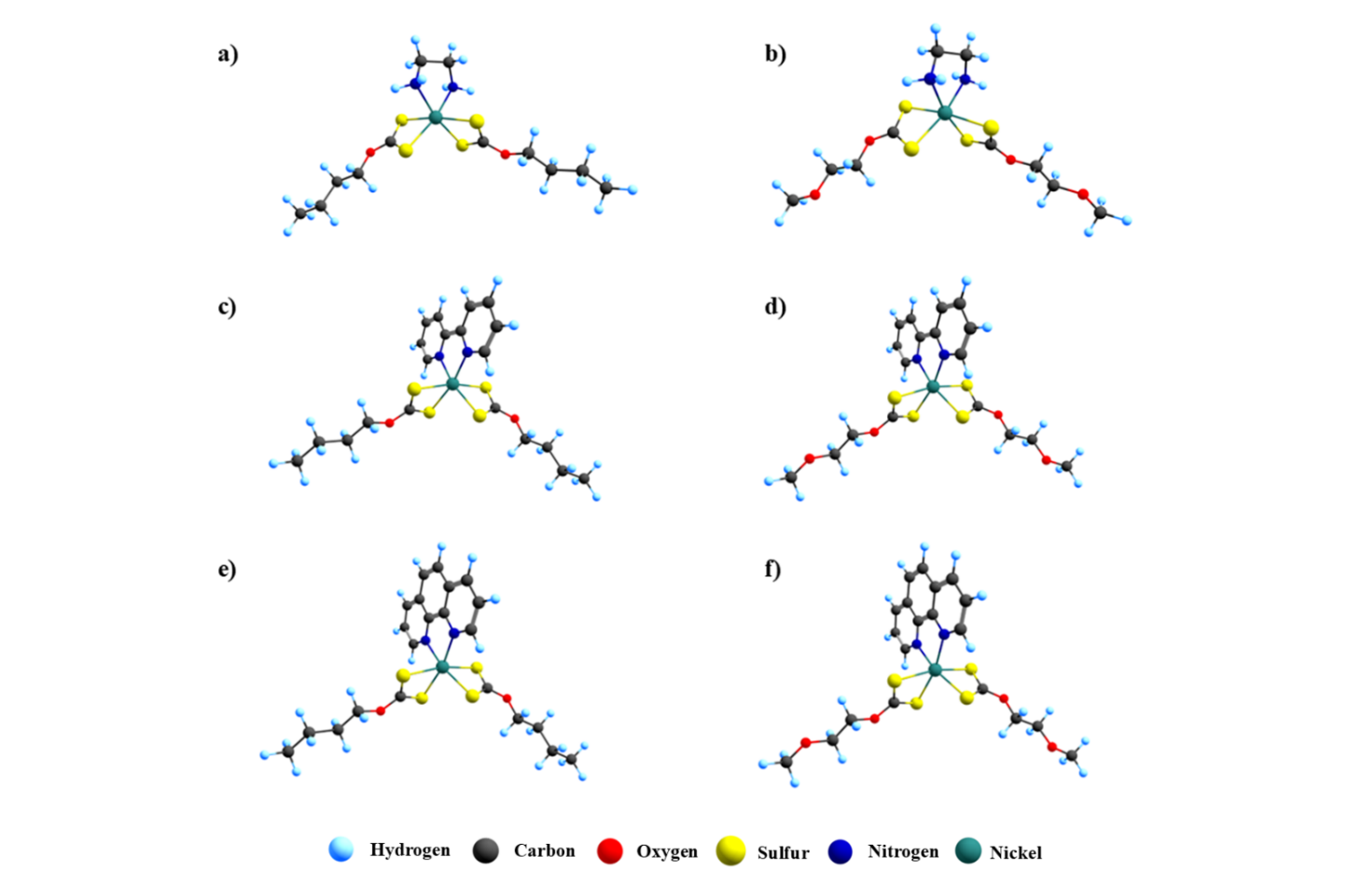
**

**Figure S7.** Optimized structures of complexes (CPCM = ethanol): **a)** [Ni(en)(L1)_2_]; **b)** [Ni(en)(L2)_2_]; **c)** [Ni(bpy)(L1)_2_]; **d)** [Ni(bpy)(L2)_2_]; **e)** [Ni(phen)(L1)_2_]; **f)** [Ni(phen)(L2)_2_].

**Table S2.** Ni-S and Ni-N bond lengths (Å) for homoleptic and heteroleptic Ni(II) complexes, in ethanol (CPCM).

| **Complexes** | **Bond length / Å** | |
| --- | --- | --- |
|  | **Ni-S** | **Ni-N** |
| [Ni(L1)_2_] | 2.234-2.236 | - |
| [Ni(L2)_2_] | 2.234-2.237 | - |
| [Ni(en)(L1)_2_] | 2.460-2.484 | 2.140-2.147 |
| [Ni(en)(L2)_2_] | 2.459-2.485 | 2.142-2.143 |
| [Ni(bpy)(L1)_2_] | 2.468-2.474 | 2.098-2.100 |
| [Ni(bpy)(L2)_2_] | 2.467-2.478 | 2.097-2.098 |
| [Ni(phen)(L1)_2_] | 2.461-2.477 | 2.109-2.121 |
| [Ni(phen)(L2)_2_] | 2.461-2.475 | 2.114-2.118 |

**Table S3.** S-Ni-S, N-Ni-N angles (°) for homoleptic and heteroleptic Ni(II) complexes, in ethanol (CPCM).

| **Complexes** | **Angles / °** | | |
| --- | --- | --- | --- |
|  | **S-Ni-S** | **N-Ni-N** | **N-Ni-S** |
| [Ni(L1)_2_] | 78.74-101.37 | - | - |
| [Ni(L2)_2_] | 78.79-101.37 | - | - |
| [Ni(en)(L1)_2_] | 73.00-98.75 | 81.36 | 91.05-92.47 |
| [Ni(en)(L2)_2_] | 73.03-98.92 | 81.45 | 90.97-92.63 |
| [Ni(bpy)(L1)_2_] | 72.89-98.25 | 77.65 | 94.56-96.82 |
| [Ni(bpy)(L2)_2_] | 72.90-97.85 | 77.76 | 94.73-96.93 |
| [Ni(phen)(L1)_2_] | 73.12-98.12 | 78.81 | 93.26-96.38 |
| [Ni(phen)(L2)_2_] | 72.95-99.55 | 78.95 | 91.84-96.05 |

1. **Thermochemical analysis**

**Table S4.** Gibbs free energy (ΔG), enthalpy (ΔH), and entropic contribution (TΔS) values (kcal mol^-1^) calculated for Ni(II) complexes in sequence 1, with BSSE corrections, in ethanol (CPCM).

| **Complexes** | **ΔG (kcal mol^-1^)** | **ΔH (kcal mol^-1^)** | **TΔS (kcal mol^-1^)** |
| --- | --- | --- | --- |
| **[Ni(en)(L1)_2_]** | -27.66 | -18.37 | 9.28 |
| **[Ni(bpy)(L1)_2_]** | -23.35 | -15.48 | 7.87 |
| **[Ni(phen)(L1)_2_]** | -22.23 | -14.75 | 7.47 |
| **[Ni(en)(L2)_2_]** | -26.72 | -17.74 | 8.98 |
| **[Ni(bpy)(L2)_2_]** | -22.12 | -14.22 | 7.89 |
| **[Ni(phen)(L2)_2_]** | -22.86 | -14.11 | 8.75 |
| **[Ni(en)_2_(H_2_O)_2_]^2+^** | 10.31 | 31.33 | 21.02 |
| **[Ni(bpy)_2_(H_2_O)_2_]^2+^** | 6.99 | 28.85 | 21.86 |
| **[Ni(phen)_2_(H_2_O)_2_]^2+^** | 6.60 | 29.11 | 22.51 |

**Table S5.** Gibbs free energy (ΔG), enthalpy (ΔH), and entropic contribution (TΔS) values (kcal mol^-1^) calculated for Ni(II) complexes in sequence 2, with BSSE corrections, in ethanol (CPCM).

| **Complexes** | **ΔG (kcal mol^-1^)** | **ΔH (kcal mol^-1^)** | **TΔS (kcal mol^-1^)** |
| --- | --- | --- | --- |
| **[Ni(en)(L1)_2_]** | -24.74 | -13.89 | 10.85 |
| **[Ni(bpy)(L1)_2_]** | -26.70 | -18.77 | 7.93 |
| **[Ni(phen)(L1)_2_]** | -23.03 | -14.83 | 8.19 |
| **[Ni(en)(L2)_2_]** | -24.57 | -17.20 | 7.36 |
| **[Ni(bpy)(L2)_2_]** | -23.28 | -16.16 | 7.12 |
| **[Ni(phen)(L2)_2_]** | -24.41 | -15.78 | 8.63 |
| **[Ni(L1)_2_(H_2_O)_2_]** | 7.40 | 29.19 | 21.79 |
| **[Ni(L2)_2_(H_2_O)_2_]** | 8.15 | 30.79 | 22.63 |

|  | **G (Eh)** | **H (Eh)** |
| --- | --- | --- |
| **[Ni(CH_3_COO)_2_.4H_2_O]** | -2271.21 | -2271.14 |
| **en** | -190.48 | -190.45 |
| **bpy** | -495.37 | -495.32 |
| **phen** | -571.61 | -571.56 |
| **L1** | -1067.68 | -1067.64 |
| **L2** | -1103.61 | -1103.56 |
| **[Ni(en)(L1)_2_]** | -3833.99 | -3833.89 |
| **[Ni(bpy)(L1)_2_]** | -4138.87 | -4138.77 |
| **[Ni(phen)(L1)_2_]** | -4215.11 | -4215.00 |
| **[Ni(en)(L2)_2_]** | -3905.83 | -3905.74 |
| **[Ni(bpy)(L2)_2_]** | -4210.72 | -4210.62 |
| **[Ni(phen)(L2)_2_]** | -4286.96 | -4286.85 |
| **[Ni(en)_2_(H_2_O)_2_]^2+^** | -2041.96 | -2041.89 |
| **[Ni(bpy)_2_(H_2_O)_2_]^2+^** | -2651.73 | -2651.65 |
| **[Ni(phen)_2_(H_2_O)_2_]^2+^** | -2804.20 | -2804.12 |
| **[Ni(L1)_2_(H_2_O)_2_]** | -3796.36 | -3796.27 |
| **[Ni(L2)_2_(H_2_O)_2_]** | -3868.21 | -3868.12 |

**Table S6.** Computed Gibbs free energies (G) and enthalpies (H) for all species at the M06-L/def2-TZVP/CPCM (ethanol) level of theory, including BSSE correction, reported in Hartree (Eh). Conversion to kcal mol^-1^ was performed using a factor of 627.503.

1. **UV-Vis electronic analysis**

**Table S7.** Experimental and DFT calculated absorption wavelengths and energies for the [Ni(en)(L1)_2_] and [Ni(en)(L2)_2_] complexes.

| **Experimental** | | **DFT** | | |
| --- | --- | --- | --- | --- |
| **Wavelength (nm)** | **Energy (eV)** | **Wavelength (nm)** | **Energy (eV)** | **Electronic transitions** |
| **[Ni(en)(L1)_2_]** | | | | |
| 180 | 6.89 | 176 | 7.04 | H-9α → L+2α (18%), H-7β → L+6β (30%), HOMOα → L+21α (8%), H-6α → L+4α (5%) |
| 215 | 5.77 | 202 | 6.14 | H-11α → LUMOα (17%), H-1β → L+8β (13%), H-12α → LUMOα (5%), HOMOα → L+11α (8%), HOMOα → L+12α (8%), HOMOα → L+13α (7%), H-10β → L+2β (8%), H-9β → L+2β (7%), H-8β → L+2β (5%) |
| 228 | 5.48 | 236 | 5.25 | H-1α → L+4α (38%), HOMOα → L+5α (28%), H-1β → L+4β (14%) |
| 244 | 5.08 | 256 | 4.84 | H-9β → L+1β (13%), H-7β → L+2β (10%), H-6β → L+3β (11%), H-3α → L+1α (5%) |
| 274 | 4.52 | 272 | 4.56 | H-5α → L+1α (49%), H-8β → L+3β (10%), H-6α → L+1α (5%), H-4β → L+3β (5%) |
| 310 | 3.99 | 303 | 4.09 | H-7β → LUMOβ (47%), H-4β → L+2β (11%), H-4β → L+3β (24%) |
| **[Ni(en)(L2)_2_]** | | | | |
| 189 | 6.56 | 190 | 6.52 | H-18β → L+1β (54%), H-4α → L+4α (8%), H-2α → L+7α (5%), H-7β → L+4β (5%), H-3β → L+7β (6%) |
| 229 | 6.28 | 234 | 5.29 | H-10α → L+1α (12%), H-9α → L+1α (35%), H-1β → L+4β (17%), H-11β → L+3β (5%) |
| 246 | 5.04 | 253 | 4.90 | H-9β → L+2β (59%), H-8α → LUMOα (7%), H-8β → L+2β (5%) |
| 282 | 4.39 | 284 | 4.36 | H-9β → L+1β (50%), H-6β → L+2β (17%), H-11β → LUMOβ (8%), H-8β → L+1β (5%) |
| 310 | 3.99 | 312 | 3.97 | H-4β → L+2β (25%), H-3β → L+2β (47%), H-3β → L+3β (6%) |
| 364 | 3.41 | 364 | 3.41 | H-4β → LUMOβ (36%), H-3β → LUMOβ (37%), HOMOβ → L+3β (8%) |

H = Homo; L = Lumo.

**Table S8.** Molecular orbital energies (eV) from NBO analysis for the [Ni(en)(L1)_2_] complex.

| **Orbital** | **Energy**  **(eV)** | **NBO Analysis of Molecular Orbitals** |
| --- | --- | --- |
| Lα+5 | 1.172 | 100%: BD* C28- H29* |
| Lα+4 | 0.869 | 23%: LV Ni35(lv) 20%: BD* C12- S13* 20%: BD* C8- S9* 19%: BD* S11- C12* 18%: BD* C8- S10* |
| Lα+2 | 0.419 | 35%: BD* C38- H40* 34%: BD* C36- H43* 31%: RY H44(ry) |
| Lα+1 | -1.356 | 55%: BD* C12- S13* 30%: LP S11(lp) 15%: LP O14(lp) |
| Lα | -1.370 | 55%: BD* C8- S9* 30%: LP S10(lp) 15%: LP O7(lp) |
| Hα | -4.111 | 22%: LP S13(lp) 21%: LP S9(lp) 17%: LP Ni35(lp) 16%: LP S10(lp) 14%: LP S11(lp) |
| Hα-1 | -4.411 | 26%: LP S11(lp) 25%: LP S10(lp) 23%: LP Ni35(lp) 13%: LP N39(lp) 13%: LP N37(lp) |
| Hα-5 | -5.928 | 30%: LP Ni35(lp) 16%: BD C8- S9 12%: BD C12- S13 11%: LP S11(lp) 11%: LP O14(lp) 10%: LP S11(lp) |
| Hα-9 | -6.765 | 34%: LP Ni35(lp) 16%: BD C8- S9 13%: LP O7(lp) 13%: LP Ni35(lp) 12%: LP O14(lp) |
| Hα-11 | -7.032 | 25%: LP Ni35(lp) 16%: LP O7(lp) 16%: LP O14(lp) 16%: LP S11(lp) 13%: LP S10(lp) 11%: BD C8- S9 10%: LP Ni35(lp) |
| Lβ+8 | 1.251 | 100%: BD* C21- H23* |
| Lβ+6 | 0.966 | 23%: LV Ni35(lv) 22%: BD* C8- S9* 21%: BD* C12- S13* 21%: BD* S11- C12* 13%: BD* C8- S10* |
| Lβ+3 | -1.334 | 56%: BD* C12- S13* 25%: LP S11(lp) 19%: LP O14(lp) |
| Lβ+1 | -1.915 | 53%: LV Ni35(lv) 16%: LP S10(lp) 15%: LP S11(lp) 16%: LP N37(lp) |
| Lβ | -2.001 | 55%: LV Ni35(lv) 24%: LP S13(lp) 21%: LP S9(lp) |
| Hβ | -4.816 | 29%: LP Ni35(lp) 16%: LP S10(lp) 14%: LP Ni35(lp) 14%: LP S11(lp) 13%: BD C12- S13 |
| Hβ-1 | -4.938 | 39%: LP Ni35(lp) 26%: BD C12- S13 20%: LP S10(lp) 15%: LP Ni35(lp) |
| Hβ-4 | -5.465 | 27%: LP Ni35(lp) 19%: LP S9(lp) 18%: LP S10(lp) 18%: LP S13(lp) 18%: LP S11(lp) |
| Hβ-6 | -5.789 | 31%: LP Ni35(lp) 20%: BD C12- S13 17%: LP S10(lp) 14%: LP S9(lp) 12%: BD C8- S9 |
| Hβ-7 | -6.239 | 17%: LP S13(lp) 15%: LP S11(lp) 15%: LP S10(lp) 15%: BD C8- S9 14%: LP S11(lp) 13%: LP S9(lp) 12%: LV Ni35(lv) 11%: LP Ni35(lp) |
| Hβ-9 | -6.668 | 17%: LP Ni35(lp) 16%: LP S9(lp) 16%: LP S11(lp) 14%: LP S10(lp) 14%: LP O7(lp) 13%: LP O14(lp) 10%: BD C12- S13 |

BD* = antibonding orbital; BD = bonding orbital; LP = Lone pair; LV = lone vacancy; Ry = Rydberg orbital; H = HOMO; L = LUMO.

**Table S9.** Molecular orbital energies (eV) from NBO analysis for the [Ni(en)(L2)_2_] complex.

| **Orbital** | **Energy**  **(eV)** | **NBO Analysis of Molecular Orbitals** |
| --- | --- | --- |
| Lα+1 | -1.455 | 53%: BD* C29- S40* 27%: LP S39(lp) 20%: LP O34(lp) |
| Lα | -1.468 | 53%: BD* C25- S38* 27%: LP S37(lp) 20%: LP O35(lp) |
| Hα | -4.197 | 25%: LP S39(lp) 24%: LP S37(lp) 19%: LP Ni43(lp) 18%: LP S38(lp) 14%: LP S40(lp) |
| Hα-9 | -6.638 | 65%: LP Ni43(lp) 18%: LP Ni43(lp) 17%: LP S39(lp) |
| Lβ+4 | 0.377 | 35%: BD* H13- C31* 34%: BD* H14- C30* 31%: RY H9(ry) |
| Lβ+2 | -1.447 | 39%: BD* C25- S38* 17%: LP S37(lp) 16%: LP O35(lp) 15%: LP S38(lp) 13%: LP S37(lp) |
| Lβ+1 | -2.006 | 77%: LV Ni43(lv) 23%: LP S40(lp) |
| Lβ | -2.083 | 57%: LV Ni43(lv) 24%: LP S39(lp) 19%: LP S37(lp) |
| Hβ | -4.908 | 39%: LP Ni43(lp) 18%: BD C25- S38 16%: LP S39(lp) 13%: LP Ni43(lp) 14%: LP S40(lp) |
| Hβ-1 | -5.021 | 39%: LP Ni43(lp) 27%: LP S39(lp) 18%: BD C25- S38 16%: LP Ni43(lp) |
| Hβ-3 | -5.429 | 25%: LP S38(lp) 22%: BD C29- S40 19%: LP S39(lp) 17%: LP S37(lp) 17%: LP Ni43(lp) |
| Hβ-4 | -5.546 | 25%: LP Ni43(lp) 18%: LP S37(lp) 15%: LP S39(lp) 15%: LP S40(lp) 14%: BD C25- S38 13%: LP S39(lp) |
| Hβ-9 | -6.345 | 18%: LP S39(lp) 17%: LP S37(lp) 17%: LP S40(lp) 14%: LP S38(lp) 12%: BD C29- S40 12%: LV Ni43(lv) 10%: LP S37(lp) |
| Hβ-18 | -8.362 | 22%: LP N42(lp) 21%: LP N41(lp) 15%: BD H14- C30 15%: BD C30- C31 15%: BD H13- C31 12%: LV Ni43(lv) |

BD* = antibonding orbital; BD = bonding orbital; LP = Lone pair; LV = lone vacancy; Ry = Rydberg orbital; H = HOMO; L = LUMO.

**Table S10.** Experimental and DFT calculated absorption wavelengths and energies for the [Ni(bpy)(L1)_2_] and [Ni(bpy)(L2)_2_] complexes.

| **Experimental** | | **DFT** | | |
| --- | --- | --- | --- | --- |
| **Wavelength (nm)** | **Energy (eV)** | **Wavelength (nm)** | **Energy (eV)** | **Electronic transitions** |
| **[Ni(bpy)(L1)_2_]** | | | | |
| 185 | 6.70 | 200 | 6.19 | H-20β → L+1β (40%), H-20α → L+2α (9%), H-12α → L+5α (8%), H-19β → L+3β (5%), H-2β → L+10β (7%) |
| 224 | 5.53 | 229 | 5.41 | H-9β → L+5β (27%), H-9β → L+6β (18%), H-8β → L+6β (19%), H-8α → L+3α (9%), H-8α → L+4α (8%) |
| 242 | 5.12 | 249 | 4.98 | H-15α → LUMOα (37%), H-4α → L+5α (28%), H-7β → L+5β (5%) |
| 258 | 4.80 | 255 | 4.86 | H-6α → L+3α (10%), H-6β → L+6β (12%), H-15α → LUMOα (6%), H-3α → L+4α (5%), H-11β → L+4β (7%) |
| 273 | 4.54 | 279 | 4.44 | H-3α → L+5α (23%), H-9β → L+1β (12%), H-3β → L+7β (11%), H-11α → LUMOα (7%), H-8α → L+1α (7%), H-10β → LUMOβ (9%) |
| 290 | 4.27 | 283 | 4.38 | H-11α → LUMOα (18%), H-8α → L+1α (20%), H-10β → LUMOβ (19%), H-9β → L+1β (14%) |
| 307 | 4.04 | 309 | 4.01 | H-3α → L+4α (26%), H-4β → L+6β (11%), H-3β → L+6β (18%), H-7β → L+2β (8%), HOMOβ → L+6β (5%), HOMOβ → L+7β (5%) |
| 343 | 3.61 | 340 | 3.64 | H-9α → LUMOα (36%), H-1β → L+6β (26%) |
| **[Ni(bpy)(L2)_2_]** | | | | |
| 197 | 6.29 | 205 | 6.05 | H-21α → L+1α (10%), H-20β → L+2β (21%), H-17β → L+3β (16%), H-18α → L+2α (7%), H-17α → L+2α (9%), H-20β → L+1β (8%) |
| 219 | 5.66 | 229 | 5.41 | H-1α → L+7α (13%), HOMOα → L+11α (71%), H-10α → L+3α (7%) |
| 238 | 5.21 | 236 | 5.25 | H-11α → L+3α (17%), H-10β → L+5β (31%), H-6α → L+3α (9%) |
| 251 | 4.94 | 253 | 4.90 | H-16α → LUMOα (13%), H-12α → L+1α (17%), H-15β → LUMOβ (14%), H-12β → L+1β (16%), H-4α → L+5α (5%), H-16β → LUMOβ (9%) |
| 266 | 4.66 | 267 | 4.64 | H-5α → L+3α (13%), H-5α → L+4α (31%), H-6α → L+4α (5%), H-10β → L+3β (8%) , H-10β → L+6β (9%) |
| 275 | 4.51 | 277 | 4.47 | H-10α → L+1α (13%), H-11β → L+1β (29%), H-3β → L+7β (27%), H-12β → LUMOβ (6%) |
| 292 | 4.24 | 290 | 4.27 | H-9α → L+1α (23%), H-8α → L+2α (12%), H-6β → L+6β (18%), H-9β → L+3β (6%) |
| 305 | 4.06 | 309 | 4.01 | H-5α → L+2α (42%), H-3α → L+4α (6%), H-7β → L+3β (9%), H-4β → L+5β (5%), H-4β → L+6β (6%), H-3β → L+5β (6%) |
| 307 | 4.04 | 307 | 4.04 | H-13β → LUMOβ (77%), H-14α → LUMOα (5%) |

H = Homo; L = Lumo.

**Table S11.** Molecular orbital energies (eV) from NBO analysis for the [Ni(bpy)(L1)_2_] complex.

| **Orbital** | **Energy**  **(eV)** | **NBO Analysis of Molecular Orbitals** |
| --- | --- | --- |
| Lα+5 | -0.665 | 26%: BD* C12- C13* 23%: BD* C1- C2* 19%: BD* C3- C5* 17%: BD* C7- N17* 15%: BD C6- C11 |
| Lα+4 | -1.158 | 52%: BD* C26- S27* 27%: LP S28(lp) 21%: LP O25(lp) |
| Lα+3 | -1.174 | 52%: BD* C30- S31* 28%: LP S29(lp) 20%: LP O32(lp) |
| Lα+1 | -2.159 | 21%: BD* C6- C11* 20%: BD* C4- N18* 17%: BD* C12- C13* 16%: BD* C3- C5* 14%: BD C1- C2 12%: BD C3- C5 |
| Lα | -2.904 | 20%: BD* C7- N17* 18%: BD* C1- C2* 16%: BD* C3- C5* 13%: BD C6- C11 12%: BD C12- C13 11%: BD C4- N18 10%: BD* C4- N18* |
| Hα | -3.941 | 25%: LP S28(lp) 24%: LP S29(lp) 18%: LP S31(lp) 18%: LP Ni53(lp) 15%: LP S27(lp) |
| Hα-3 | -5.168 | 22%: LP S28(lp) 21%: LP S29(lp) 20%: BD C30- S31 14%: BD C26- S27 12%: BD* C30- S31* 11%: LP Ni53(lp) 10%: BD* C26- S27* |
| Hα-4 | -5.672 | 31%: LP S29(lp) 31%: LP S28(lp) 13%: LP S28(lp) 13%: LP S29(lp) 12%: LP Ni53(lp) |
| Hα-6 | -6.032 | 20%: LP Ni53(lp) 20%: LP Ni53(lp) 18%: BD C26- S27 14%: LP O32(lp) 14%: BD C30- S31 13%: LP O25(lp) 13%: LP S29(lp) |
| Hα-8 | -6.625 | 19%: LP S31(lp) 18%: LP Ni53(lp) 16%: LP Ni53(lp) 15%: LP S31(lp) 15%: LP O25(lp) 14%: LP Ni53(lp) 13%: LP N18(lp) |
| Hα-9 | -6.656 | 22%: LP Ni53(lp) 13%: LP S29(lp) 11%: LP O32(lp) 10%: LP S31(lp) 10%: LP Ni53(lp)  9%: LP S27(lp)  9%: LP S27(lp) |
| Hα-11 | -6.742 | 31%: LP Ni53(lp) 28%: LP S27(lp) 24%: LP N17(lp) 17%: LP N18(lp) |
| Hα-15 | -7.904 | 28%: LP Ni53(lp) 19%: LP O25(lp) 17%: LP O32(lp) 16%: LP Ni53(lp) 15%: LP S28(lp) |
| Lβ+7 | -0.675 | 26%: BD* C12- C13* 23%: BD* C1- C2* 19%: BD* C3- C5* 17%: BD* C7- N17* 15%: BD C6- C11 |
| Lβ+6 | -1.131 | 33%: BD* C26- S27* 19%: LP S28(lp) 15%: BD* C30- S31* 14%: LP S27(lp) 14%: LP O25(lp) |
| Lβ+5 | -1.155 | 42%: BD* C30- S31* 23%: LP S29(lp) 19%: BD* C26- S27* 16%: LP O32(lp) |
| Lβ+4 | -1.158 | 52%: BD* C26- S27* 28%: LP S28(lp)  20%: LP O25(lp) |
| Lβ+1 | -2.153 | 20%: BD* C6- C11* 19%: BD* C4- N18* 16%: BD* C12- C13* 15%: BD* C3- C5* 14%: BD C1- C2 10%: BD C3- C5 |
| Lβ | -2.865 | 20%: BD* C7- N17* 18%: BD* C1- C2* 16%: BD* C3- C5* 13%: BD C6- C11 12%: BD C12- C13 11%: BD C4- N18 10%: BD* C12- C13* |
| Hβ | -4.751 | 30%: LP Ni53(lp) 29%: LP S28(lp) 17%: BD C26- S27 14%: LP Ni53(lp) 13%: LP Ni53(lp) 12%: LP S27(lp) |
| Hβ-1 | -4.778 | 21%: BD C30- S31 20%: LP S29(lp) 20%: LP Ni53(lp) 15%: LP Ni53(lp) 13%: LP S28(lp) 11%: BD* C30- S31* |
| Hβ-3 | -5.177 | 20%: LP S31(lp) 20%: LP S27(lp) 17%: LP S28(lp) 17%: LP S29(lp) 13%: LP Ni53(lp) 13%: LV Ni53(lv) |
| Hβ-6 | -5.619 | 36%: LP Ni53(lp) 25%: LP S28(lp) 21%: LP S29(lp) 18%: LP Ni53(lp) |
| Hβ-8 | -6.420 | 20%: LP S29(lp) 18%: BD C26- S27 17%: LP O32(lp) 17%: LP O25(lp) 14%: BD C30- S31 14%: LP S28(lp) |
| Hβ-9 | -6.615 | 18%: BD C26- S27 16%: LP O25(lp) 15%: LP O32(lp) 15%: BD C30- S31 12%: LP Ni53(lp) 12%: LP Ni53(lp) 12%: LP S29(lp) |
| Hβ-10 | -6.719 | 19%: BD C12- C13 18%: BD C3- C5 12%: BD* C4- N18* 11%: BD C1- C2 11%: BD C7- N17 10%: BD* C6- C11* 10%: BD* C1- C2*  9%: BD C6- C11 |
| Hβ-11 | -6.742 | 31%: LP Ni53(lp)  28%: LP S27(lp)  24%: LP N17(lp)  17%: LP N18(lp) |
| Hβ-20 | -8.382 | 30%: BD C22- C46 27%: BD C49- H52 22%: BD C36- C39 21%: BD C42- H45 |

BD* = antibonding orbital; BD = bonding orbital; LP = Lone pair; LV = lone vacancy; Ry = Rydberg orbital; H = HOMO; L = LUMO.

**Table S12.** Molecular orbital energies (eV) from NBO analysis for the [Ni(bpy)(L2)_2_] complex.

| **Orbital** | **Energy**  **(eV)** | **NBO Analysis of Molecular Orbitals** |
| --- | --- | --- |
| Lα+11 | 1.404 | 26%: BD* C30- S31* 25%: BD* S29- C30* 25%: BD* C26- S28* 24%: BD* C26- S27* |
| Lα+4 | -1.256 | 45%: BD* C26- S27* 24%: LP S28(lp) 17%: LP O25(lp) 14%: BD* C30- S31* |
| Lα+3 | -1.270 | 44%: BD* C30- S31* 24%: LP S29(lp) 17%: LP O32(lp) 15%: BD* C26- S27* |
| Lα+2 | -1.916 | 20%: BD* C6- C11* 18%: BD* C4- N18* 12%: BD C3- C5 11%: BD* C1- C2* 10%: BD* C7- N17* 10%: BD C12- C13 10%: BD* C12- C13*  9%: BD C1- C2 |
| Lα+1 | -2.204 | 24%: BD* C6- C11* 22%: BD* C4- N18* 20%: BD* C12- C13* 18%: BD* C3- C5* 16%: BD C1- C2 |
| Lα | -2.951 | 20%: BD* C7- N17* 18%: BD* C1- C2* 16%: BD* C3- C5* 13%: BD C6- C11 12%: BD C12- C13 11%: BD C4- N18 10%: BD* C4- N18* |
| Hα | -4.027 | 25%: LP S28(lp) 24%: LP S29(lp) 18%: LP Ni47(lp) 18%: LP S31(lp) 15%: LP S27(lp) |
| Hα-5 | -5.933 | 33%: LP Ni47(lp) 20%: BD C26- S27 18%: LP Ni47(lp) 15%: LP S29(lp) 14%: LP O25(lp) |
| Hα-8 | -6.207 | 43%: LP O51(lp)  24%: LP O50(lp)  17%: LP Ni47(lp)  17%: LP Ni47(lp) |
| Hα-9 | -6.523 | 51%: LP Ni47(lp) 33%: LP Ni47(lp) 16%: LP Ni47(lp) |
| Hα-11 | -6.746 | 40%: LP Ni47(lp) 23%: LP S29(lp) 19%: LP O32(lp) 18%: LP Ni47(lp) |
| Hα-12 | -6.780 | 18%: BD C12- C13 16%: BD C3- C5 11%: BD* C4- N18* 10%: BD C1- C2  9%: BD C6- C11  9%: LP Ni47(lp)  9%: BD* C1- C2*  9%: BD C7- N17  9%: BD* C6- C11* |
| Hα-16 | -7.861 | 34%: LP O32(lp) 25%: BD S29- C30 23%: LP O25(lp) 18%: BD C4- N18 |
| Lβ+7 | 0.966 | 25%: BD* S29- C30* 22%: BD* C26- S27* 20%: BD* C26- S28* 18%: BD* C30- S31* 15%: LV Ni47(lv) |
| Lβ+6 | 0.691 | 21%: BD* C3- H48* 21%: BD* C6- H49* 16%: RY H48(ry) 15%: RY H49(ry) 14%: BD* C1- H8* 13%: BD* C11- H14* |
| Lβ+5 | -0.709 | 26%: BD* C12- C13* 24%: BD* C1- C2* 19%: BD* C3- C5* 18%: BD* C7- N17* 13%: BD C6- C11 |
| Lβ+3 | -1.270 | 44%: BD* C30- S31* 24%: LP S29(lp) 17%: LP O32(lp) 15%: BD* C26- S27* |
| Lβ+2 | -1.916 | 20%: BD* C6- C11* 18%: BD* C4- N18* 12%: BD C3- C5 11%: BD* C1- C2* 10%: BD* C7- N17* 10%: BD C12- C13 10%: BD* C12- C13*  9%: BD C1- C2 |
| Lβ+1 | -2.204 | 24%: BD* C6- C11* 22%: BD* C4- N18* 20%: BD* C12- C13* 18%: BD* C3- C5* 16%: BD C1- C2 |
| Lβ | -2.951 | 20%: BD* C7- N17* 18%: BD* C1- C2* 16%: BD* C3- C5* 13%: BD C6- C11 12%: BD C12- C13 10%: BD C4- N18 11%: BD* C4- N18* |
| Hβ | -4.027 | 25%: LP S28(lp) 24%: LP S29(lp) 18%: LP Ni47(lp) 18%: LP S31(lp) 15%: LP S27(lp) |
| Hβ-3 | -5.254 | 22%: LP S28(lp) 21%: LP S29(lp) 20%: BD C30- S31 14%: BD C26- S27 12%: BD* C30- S31* 11%: LP Ni47(lp) 10%: BD* C26- S27* |
| Hβ-6 | -6.093 | 14%: LP Ni47(lp) 13%: LP O50(lp) 13%: LP Ni47(lp) 13%: BD C26- S27 12%: LP O51(lp) 11%: LP O32(lp) 10%: BD C30- S31  9%: LP S29(lp)  9%: LP O25(lp) |
| Hβ-10 | -6.712 | 21%: LP S31(lp) 18%: LP Ni47(lp) 17%: LP S31(lp) 17%: LP Ni47(lp) 15%: LP O25(lp) 12%: LP Ni47(lp) |
| Hβ-11 | -6.746 | 40%: LP Ni47(lp) 23%: LP S29(lp) 19%: LP O32(lp) 18%: LP Ni47(lp) |
| Hβ-12 | -6.780 | 18%: BD C12- C13 17%: BD C3- C5 11%: BD* C4- N18* 10%: BD C1- C2 10%: BD C6- C11 10%: LP Ni47(lp) 10%: BD* C1- C2*  9%: BD C7- N17  9%: BD* C6- C11* |
| Hβ-13 | -6.828 | 30%: LP Ni47(lp) 28%: LP S27(lp) 23%: LP N17(lp) 19%: LP N18(lp) |
| Hβ-15 | -7.627 | 22%: LP Ni47(lp) 16%: LP S28(lp) 15%: LP S29(lp) 14%: LP O25(lp) 13%: LP S27(lp) 13%: LP S29(lp) 12%: BD C26- S27 11%: LP S31(lp) |
| Hβ-17 | -7.970 | 39%: LP O50(lp) 16%: BD C22- O50 15%: BD C43- H46 15%: BD C43- O50 14%: BD C19- C22 11%: BD C22- H24 |
| Hβ-20 | -8.063 | 20%: BD C3- C5 18%: BD C12- C13 18%: BD C4- N18 16%: LP O25(lp) 14%: BD* C7- N17* 13%: LP Ni47(lp) 11%: LP O50(lp) |

BD* = antibonding orbital; BD = bonding orbital; LP = Lone pair; LV = lone vacancy; Ry = Rydberg orbital; H = HOMO; L = LUMO.

**
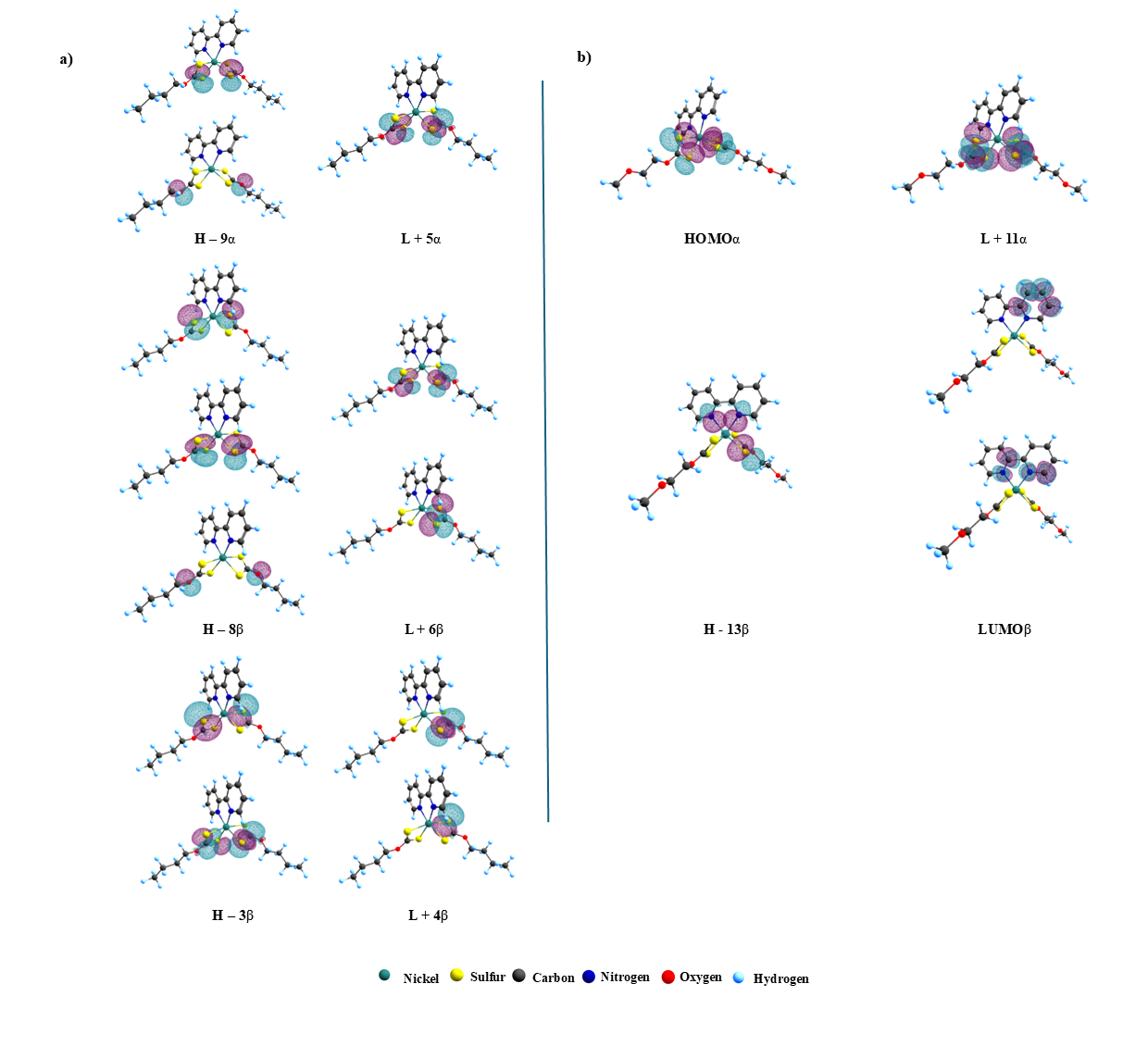
**

**Figure S8.** Selected molecular orbitals involved in the electronic transitions of the **(a)** [Ni(bpy)(L1)_2_] and **(b)** [Ni(bpy)(L2)_2_] complexes. Isosurface = 0.04 a.u.

**
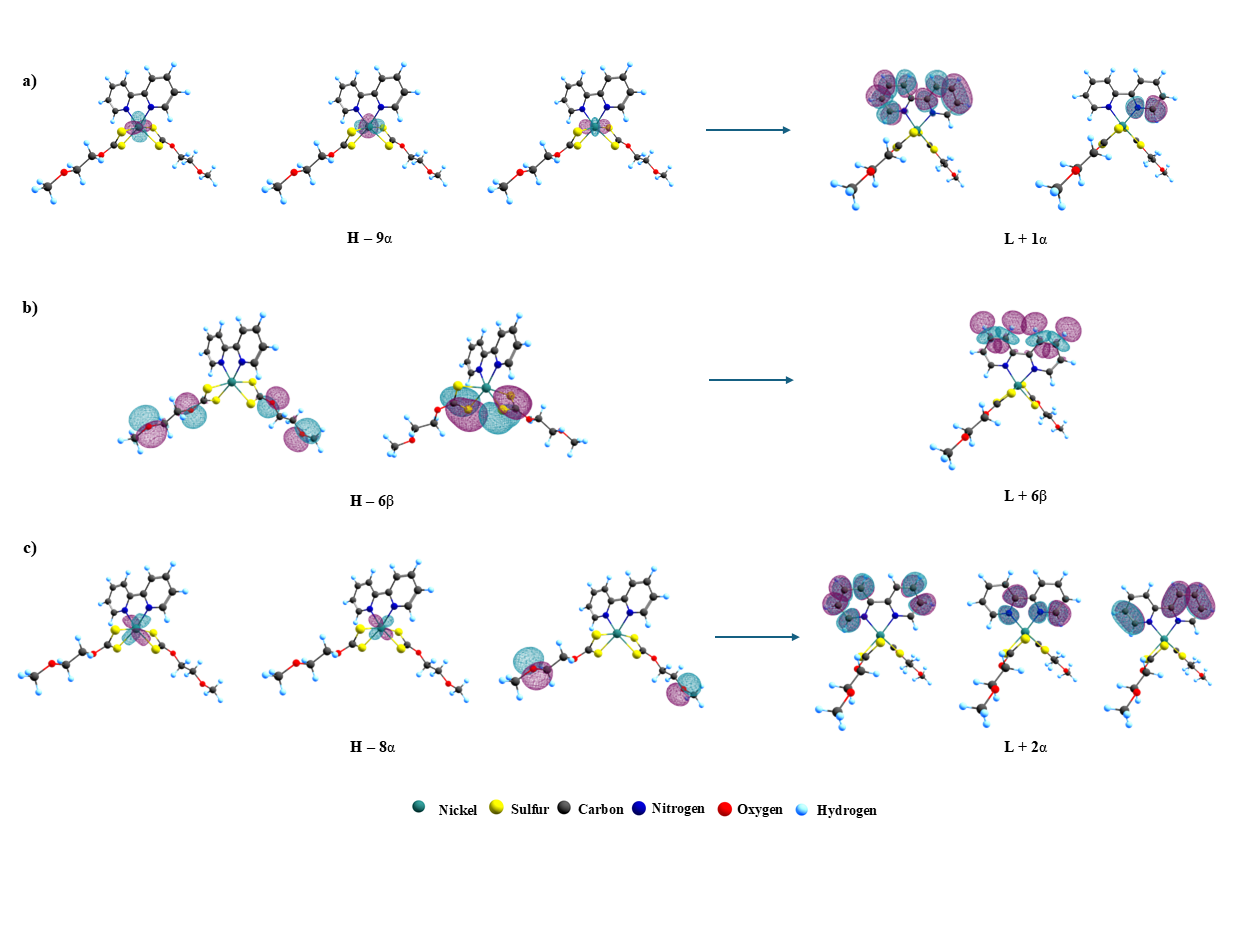
**

**Figure S9.** Molecular orbitals involved in the main electronic transitions associated with the 292 nm absorption band in the experimental UV-Vis spectrum of the [Ni(bpy)(L2)_2_] complex. Isosurface = 0.04 a.u.

**Table S13.** Experimental and DFT-calculated absorption wavelengths and energies for the [Ni(phen)(L1)_2_] and [Ni(phen)(L2)_2_] complexes.

| **Experimental** | | **DFT** | | |
| --- | --- | --- | --- | --- |
| **Wavelength (nm)** | **Energy (eV)** | **Wavelength (nm)** | **Energy (eV)** | **Electronic transitions** |
| **[Ni(phen)(L1)_2_]** | | | | |
| 196 | 6.32 | 206 | 6.02 | H-11α → L+5α (17%), HOMOα → L+17α (19%), H-10β → L+7β (13%), H-1β → L+11β (10%) |
| 225 | 5.51 | 223 | 5.56 | H-20α → L+1α (23%), H-19β → L+1β (21%), H-21α → L+1α (5%), H-13α → L+2α (6%), H-8α → L+5α (5%), H-21β → LUMOβ (5%), H-10β → L+7β (8%), H-12β → L+4β (8%) |
| 268 | 4.63 | 262 | 4.73 | H-13β → LUMOβ (24%), H-8β → L+4β (15%), H-10β → L+2β (7%) |
| 248 | 4.99 | 245 | 5.06 | H-12β → L+3β (39%), H-11β → L+3β (24%) |
| 303 | 4.09 | 309 | 4.01 | H-3α → L+4α (17%), H-11β → LUMOβ (16%), H-4β → L+6β (11%), H-3β → L+6β (17%), H-12α → LUMOα (5%) |
| 366 | 3.39 | 374 | 3.31 | H-4β → L+2β (12%), H-3β → L+2β (64%) |
| 463 | 2.68 | 456 | 2.72 | H-4α → LUMOα (42%), H-6β → L+1β (49%) |
| **[Ni(phen)(L2)_2_]** | | | | |
| 195 | 6.36 | 206 | 6.02 | H-13α → L+5α (12%), H-12α → L+5α (15%), H-12β → L+7β (15%), H-15α → L+5α (8%), H-14α → L+5α (7%) |
| 225 | 5.51 | 231 | 5.37 | H-10α → L+3α (16%), H-10α → L+4α (28%), HOMOα → L+11α (11%), H-9α → L+4α (8%), H-11β → L+6β (8%), H-10β → L+5β (6%), H-10β → L+6β (5%) |
| 267 | 4.64 | 267 | 4.64 | H-4α → L+5α (11%), H-11β → L+3β (26%), H-5α → L+4α (7%) |
| 291 | 4.26 | 279 | 4.44 | H-14β → L+1β (10%), H-7β → L+4β (53%), H-15α → L+1α (8%), H-14α → L+1α (6%) |
| 309 | 4.01 | 289 | 4.29 | H-5α → L+2α (13%), H-6β → L+6β (23%), H-5β → L+5β (12%), H-7β → L+2β (5%), H-5β → L+6β (7%) |
| 333 | 3.72 | 373 | 3.32 | H-4β → L+2β (12%), H-3β → L+2β (64%) |

H = Homo; L = Lumo.

**Table S14.** Molecular orbital energies (eV) from NBO analysis for the [Ni(phen)(L1)_2_] complex.

| **Orbital** | **Energy eV** | **NBO Analysis of Molecular Orbitals** |
| --- | --- | --- |
| Lα+17 | 2.121 | 27%: RY H13(ry) 25%: RY H11(ry) 24%: RY H14(ry) 24%: BD* C 2- H11* |
| Lα+5 | -0.912 | 20%: BD* C 9- N21* 18%: BD* C 1- C 2* 16%: BD C 3- C 5 14%: BD* C 6- C 7* 12%: BD* C 8- C15*  10%: BD* C16- C17* 10%: BD* C 3- C 5* |
| Lα+4 | -1.137 | 52%: BD* C30- S32* 28%: LP S31(lp)  20%: LP O29(lp) |
| Lα+1 | -2.807 | 19%: BD* C 6- C 7*  14%: BD* C 4- N22*  14%: BD* C 8- C15*  10%: BD C16- C17  9%: BD* C16- C17* 9%: BD* C 3- C 5*  9%: BD C 9- N21 8%: BD* C 1- C 2* 8%: BD C 1- C 2 |
| Lα | -2.871 | 22%: BD* C 1- C 2* 21%: BD* C 9- N21*  15%: BD* C 4- N22* 15%: BD C16- C17 14%: BD C 8- C15  14%: BD* C 3- C 5* |
| Hα | -3.933 | 23%: LP S32(lp) 22%: LP S33(lp)  16%: LP S35(lp)  15%: LP Ni57(lp)  14%: LP S31(lp)  10%: LP Ni57(lp) |
| Hα-3 | -5.147 | 25%: LP S33(lp) 24%: BD C34- S35 20%: BD C30- S32  17%: LP S31(lp)  14%: BD* C34- S35* |
| Hα-4 | -5.652 | 31%: LP S33(lp) 30%: LP S32(lp)  13%: LP Ni57(lp)  13%: LP S32(lp) 13%: LP S33(lp) |
| Hα-11 | -6.780 | 21%: LP Ni57(lp) 19%: LP S31(lp)  16%: LP N21(lp) 15%: BD C 6- C 7 15%: LP N22(lp)  14%: LP S35(lp) |
| Hα-20 | -8.309 | 29%: BD C 1- C 2  23%: BD C16- C17 13%: BD* C 4- N22*  12%: BD* C 3- C 5* 12%: BD* C 8- C15*  11%: BD C 9- N21 |
| Lβ+7 | -0.906 | 20%: BD* C 9- N21*  18%: BD* C 1- C 2* 15%: BD C 3- C 5  14%: BD* C 6- C 7* 12%: BD* C 8- C15*  11%: BD* C16- C17* 10%: BD* C 3- C 5* |
| Lβ+6 | -1.107 | 46%: BD* C30- S32* 18%: LP S31(lp)  18%: LP O29(lp)  18%: LP S31(lp) |
| Lβ+4 | -1.602 | 20%: BD* C16- C17*  19%: BD* C 8- C15* 19%: BD* C 4- N22*  17%: BD* C 3- C 5* 13%: BD C 6- C 7 13%: BD C 1- C 2 |
| Lβ+3 | -1.865 | 75%: LV Ni57(lv) 25%: LP S35(lp) |
| Lβ+2 | -1.903 | 54%: LV Ni57(lv)  24%: LP S32(lp)  22%: LP S33(lp) |
| Lβ+1 | -2.808 | 20%: BD* C 6- C 7* 15%: BD* C 8- C15* 14%: BD* C 4- N22* 12%: BD C16- C17  11%: BD* C 3- C 5* 10%: BD C 9- N21 9%: BD C 1- C 2 9%: BD* C16- C17* |
| Lβ | -2.828 | 23%: BD* C 1- C 2* 21%: BD* C 9- N21*  17%: BD* C 4- N22*  13%: BD C 8- C15 13%: BD C16- C17  12%: BD* C 3- C 5* |
| Hβ | -4.720 | 25%: LP Ni57(lp)  22%: BD C30- S32 18%: LP S31(lp) 12%: LP Ni57(lp) 12%: LP Ni57(lp)  11%: BD* C30- S32* |
| Hβ-3 | -5.179 | 18%: LP S35(lp)  17%: LP S32(lp)  17%: LP S31(lp)  14%: LP S33(lp)  12%: LV Ni57(lv)  10%: LP S33(lp)  10%: LP Ni57(lp) |
| Hβ-6 | -5.610 | 33%: LP Ni57(lp) 20%: BD C30- S32 17%: LP Ni57(lp) 16%: LP S33(lp) 13%: LP S33(lp) |
| Hβ-8 | -6.355 | 16%: LP O29(lp) 16%: LP S31(lp) 15%: LP O36(lp) 15%: BD C30- S32 15%: LP S33(lp) 12%: BD C6- C7 11%: BD C34- S35 |
| Hβ-10 | -6.634 | 27%: BD C 6- C 7 15%: BD* C 9- N21*  13%: BD C 4- N22  12%: BD C 3- C 5  12%: LP O36(lp) 11%: BD C 8- C15 11%: LP S33(lp) |
| Hβ-11 | -6.891 | 21%: LP S31(lp) 19%: LP S35(lp) 18%: LP S32(lp) 16%: LP S33(lp) 13%: LP O29(lp) 13%: LP S33(lp) |
| Hβ-12 | -6.906 | 22%: BD C16- C17 19%: BD C3- C5 18%: BD C1- C2 14%: BD* C8- C15 14%: BD C9- N21 13%: BD* C4- N22 |
| Hβ-13 | -7.644 | 29%: LP O29(lp) 22%: BD C30- S31 18%: LP O36(lp) 17%: BD C30- S32 14%: BD C23- O29 |
| Hβ-19 | -8.314 | 28%: BD C1- C2 23%: BD C16- C17 12%: BD* C4- N22* 12%: BD C9- N21 13%: BD* C3- C5* 12%: BD* C8- C15* |

BD* = antibonding orbital; BD = bonding orbital; LP = Lone pair; LV = lone vacancy; Ry = Rydberg orbital; H = HOMO; L = LUMO.

**
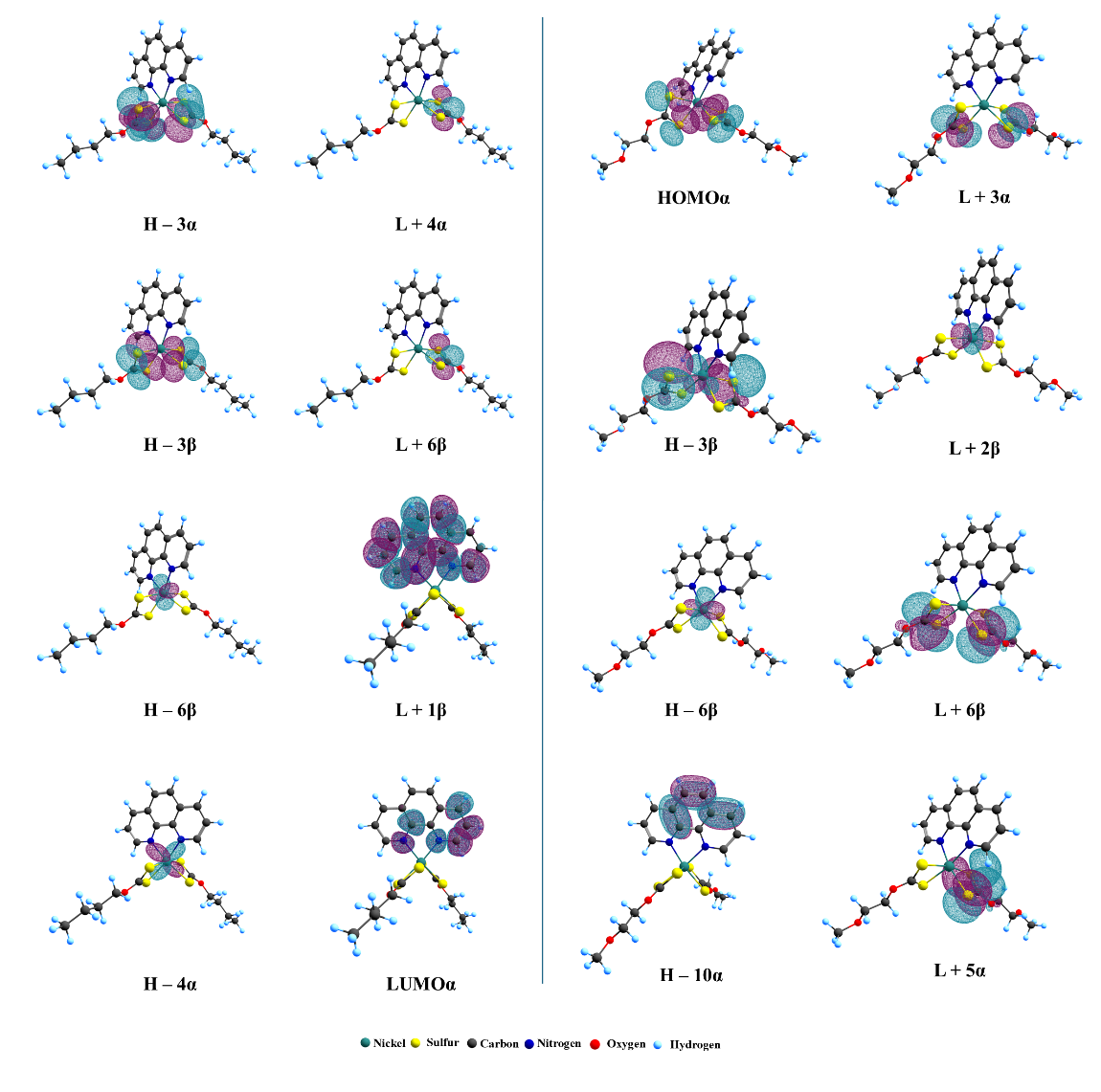
**

**Figure S10.** Selected molecular orbitals involved in the electronic transitions of the **(a)** [Ni(phen)(L1)_2_] and **(b)** [Ni(phen)(L2)_2_] complexes. Isosurface = 0.04 a.u.

**Table S15.** Molecular orbital energies (eV) from NBO analysis for the [Ni(phen)(L2)_2_] complex.

| **Orbital** | **Energy**  **eV** | **NBO Analysis of Molecular Orbitals** |
| --- | --- | --- |
| Lα+5 | -1.239 | 45% BD* C30- S31  21% LP S29  18% LP O32 16% BD* C26- S27 |
| Lα+4 | -1.648 | 44% BD* C26- S27  23% LP S28  17% LP O25 16% BD* C30- S31 |
| Lα+3 | -1.648 | 43%: BD* C30- S31*  23%: LP S29(lp)  17%: BD* C26- S27*  17%: LP O32(lp) |
| Lα | -2.873 | 22% BD*( 2) C 1- C 2*  21% BD*( 2) C 7- N17*  15% BD ( 2) C12- C13 14% BD*( 2) C 4- N18*  14% BD ( 2) C 6- C11 14% BD*( 2) C 3- C 5* |
| Hα | -4.805 | 26% LP S28(lp)  24% LP S29(lp)  17% LP ( 2) S31(lp)  16% LP ( 1)Ni47(lp)  16% LP ( 2) S27(lp) |
| Hα-10 | -6.455 | 24%: BD C50- C51 13% BD C 3- C 5  13% BD* C 7- N17* 13% BD C 6- C11  12% LP S29 12% BD C 4- N18 12% LP Ni47 |
| Hα-12 | -6.813 | 69%: LP Ni47  31% : BD C50- C51 |
| Hα-13 | -6.865 | 24% : LP Ni47  23%: LP S27  19%: LP N17  17%: LP N18  17%: LP S31 |
| Lβ+7 | -0.948 | 20%: BD* C 7- N17*  18%: BD* C 1- C 2* 15%: BD C 3- C 5  14%: BD* C50- C51* 12%: BD* C 6- C11*  11%: BD* C12- C13* 10%: BD* C 3- C 5* |
| Lβ+6 | -1.204 | 42% BD*C26- S27*  23% LP S28(lp) 19% LP S27(lp)  17% LP O25(lp) 15% BD*C30- S31* |
| Lβ+4 | -1.648 | 20%: BD* C12- C13* 19%: BD* C 4- N18*  19%: BD* C 6- C11*  16%: BD* C 3- C 5* 13%: BD C50- C51 13%: BD C 1- C 2 |
| Lβ+3 | -1.940 | 51% LV ( 2)Ni47(lv) 17% LP ( 2) S31(lp) |
| Lβ+2 | -1.985 | 55% LV Ni47  24% LP S28  21% LP S29 |
| Lβ | -2.873 | 31% BD*( 2) C 1- C 2* 28% BD*( 2) C 7- N17*  21% BD*( 2) C 4- N18*  19% BD ( 2) C12- C13  19% BD ( 2) C 6- C11  18% BD*( 2) C 3- C 5* |
| Hβ | -4.805 | 34% LP ( 1)Ni47(lp) 32% LP ( 2) S28(lp) 20% BD ( 2) C26- S27 16% LP ( 2)Ni47(lp) 16% LP ( 3)Ni47(lp) |
| Hβ-3 | -5.268 | 10% LP S31 19% LP S27  17% LP S28 17% LP S29  13% LV Ni47 12% LP S29  11% LP Ni47 |
| Hβ-6 | -5.693 | 33% LP ( 1)Ni47(lp) 21% LP ( 2) S28(lp) 17% LP ( 2)Ni47(lp)  15% LP ( 2) S29(lp) 14% LP ( 3) S29(lp) |
| Hβ-7 | -6.130 | 30%: LP O48(lp) 15%: LP S29(lp)  14%: LP S28(lp)  14%: LP Ni47(lp)  14%: LP S27(lp) 13%: LP S31(lp) |
| Hβ-11 | -6.674 | 30% BD C26- S27  27% BD C50- C51 25% LP O25  18% LP S28 |
| Hβ-12 | -6.703 | 24%: BD C50- C51 22%: LP O32(lp)  21%: LP S29(lp) 20%: BD C30- S31  13%: BD* C 7- N17* |

BD* = antibonding orbital; BD = bonding orbital; LP = Lone pair; LV = lone vacancy; Ry = Rydberg orbital; H = HOMO; L = LUMO.

1. **Natural Bond Orbital analysis**

**
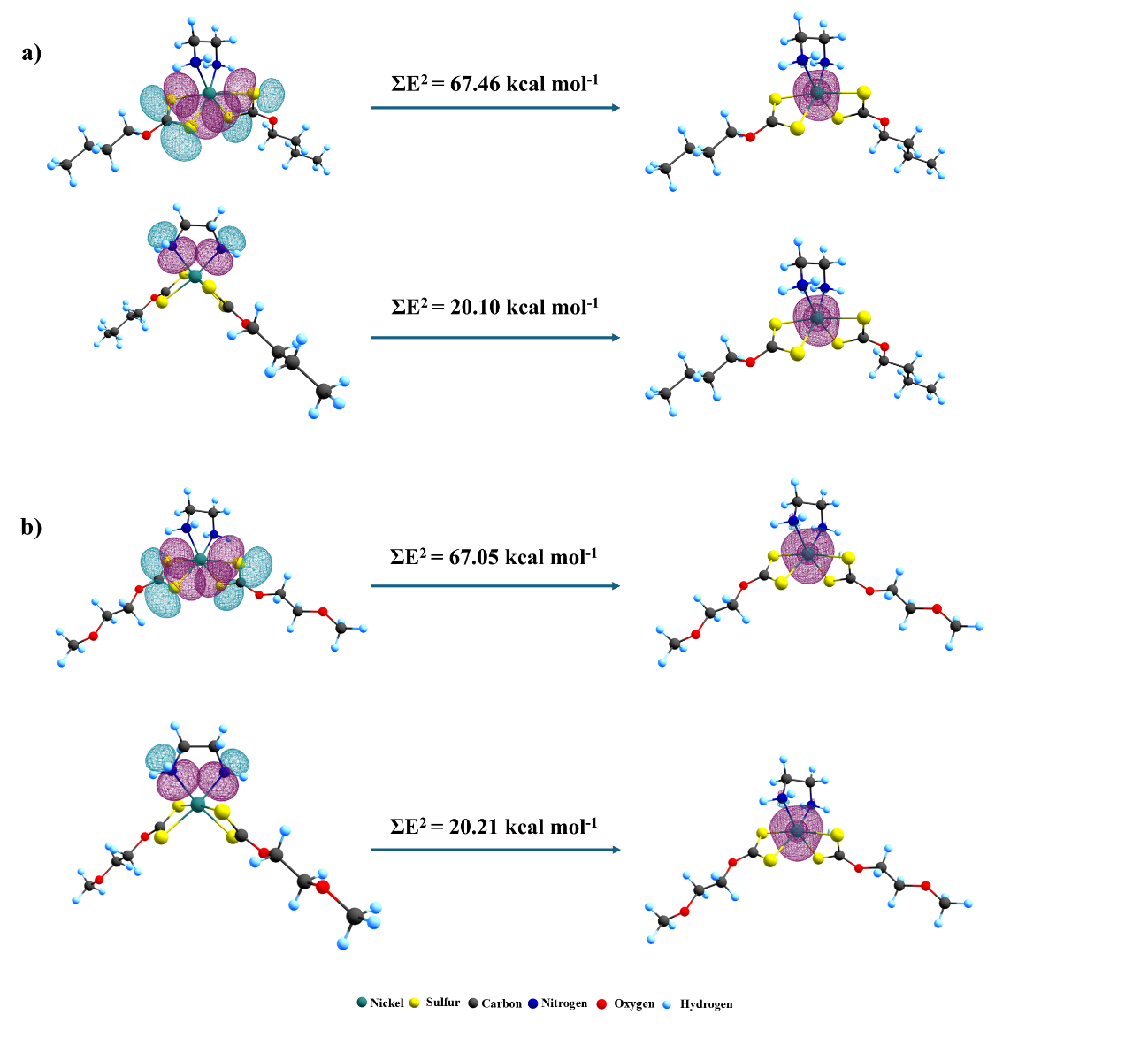
**

**Figure S11.** NBO donor-acceptor interactions in nickel complexes for: **a)** [Ni(en)(L1)_2_]; **b)** [Ni(en)(L2)_2_]. Isosurface = 0.04 a.u.

**
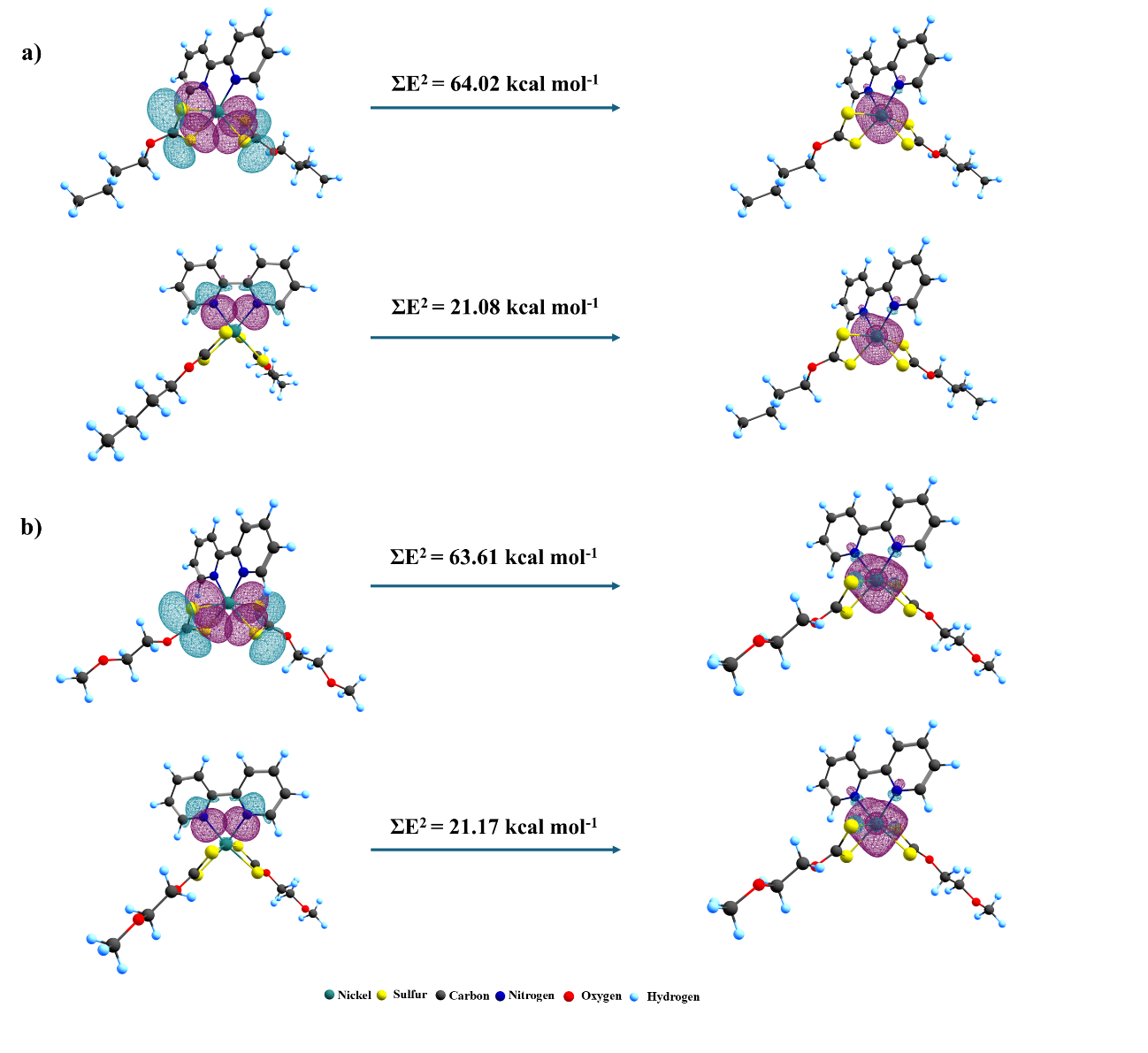
**

**Figure S12.** NBO donor-acceptor interactions in nickel complexes for: **a)** [Ni(bpy)(L1)_2_]; **b)** [Ni(bpy)(L2)_2_]. Isosurface = 0.04 a.u.

**
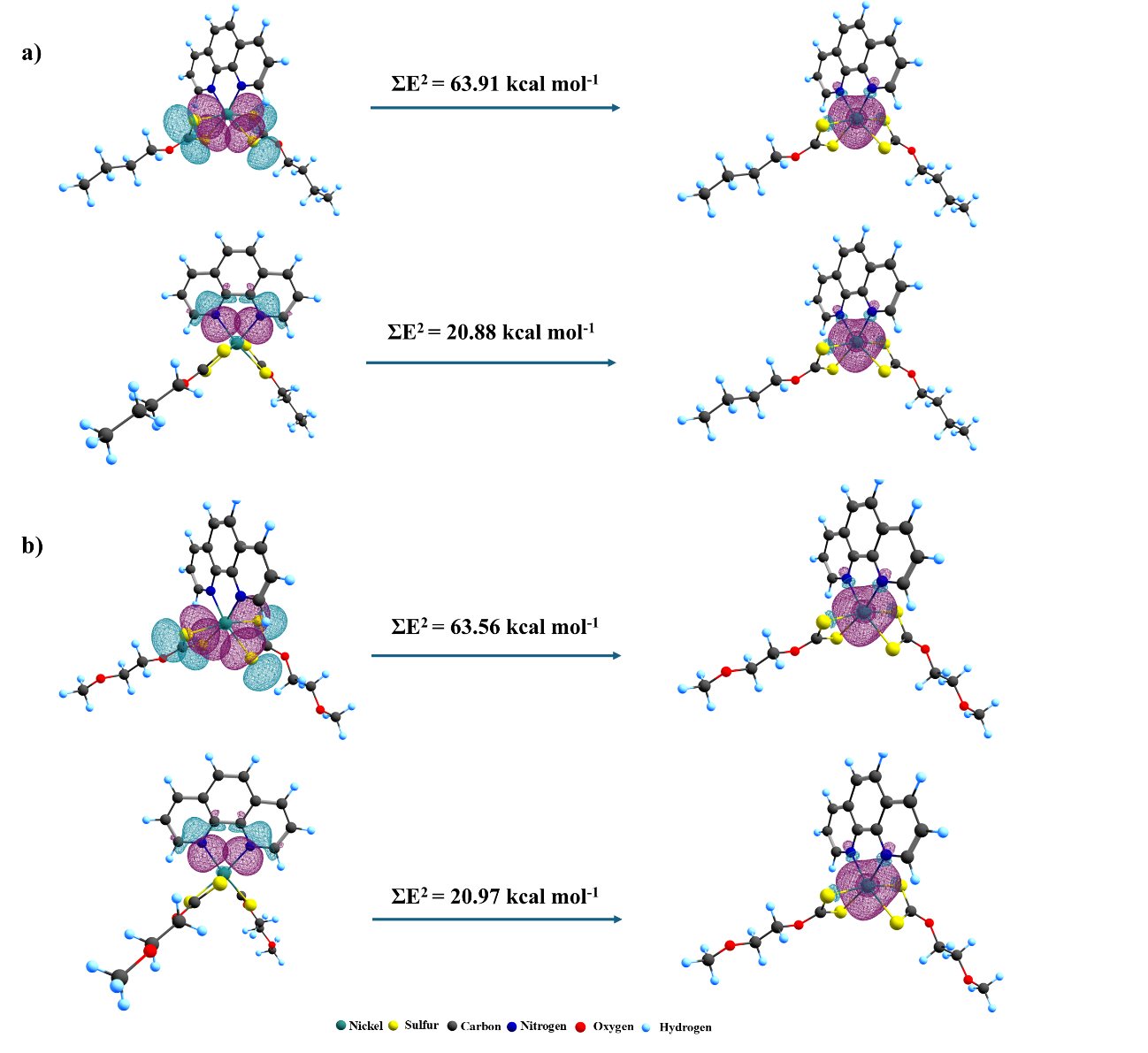
**

**Figure S13.** NBO donor-acceptor interactions in nickel complexes for: **a)** [Ni(phen)(L1)_2_]; **b)** [Ni(phen)(L2)_2_]. Isosurface = 0.04 a.u.
